# Ultrafast metal-to-ligand electron transfer driven by bond shortening revealed through dual-edge computational X-ray spectroscopy

**DOI:** 10.1038/s42004-026-02024-4

**Published:** 2026-04-24

**Authors:** Sheng-Yu Wang, Jun-Rong Zhang, Guoyan Ge, Weijie Hua

**Affiliations:** 1 School of Physics and Electronic Engineering, Research Institute of Optoelectronic Functional Materials, Jining University, Qufu, Shandong China; 2 MIIT Key Laboratory of Semiconductor Microstructure and Quantum Sensing, Department of Applied Physics, School of Physics, Nanjing University of Science and Technology, Nanjing, China

**Keywords:** Quantum chemistry, Chemical bonding

## Abstract

Understanding electron flow during chemical reactions is fundamental to ultrafast chemistry, particularly in transition-metal complexes where redox processes involve intricate coupling between electronic and nuclear dynamics. While time-resolved X-ray spectroscopy offers insight into these dynamics, interpreting spectral data to identify transient intermediates and electron transfer mechanisms remains challenging. We employ a dual-edge strategy that simultaneously simulates O K-edge and Cu L-edge X-ray absorption spectra for the paradigmatic ${{{{\rm{CuO}}}}}_{2}^{+}$ system. We show that symmetric Cu–O bond shortening drives metal-to-ligand electron transfer, converting Cu(I):O_2_ to Cu(II):${{{{\rm{O}}}}}_{2}^{\bullet -}$. Peak-by-peak analysis along the binding coordinate resolves concurrent dioxygen reduction and copper oxidation, leveraging the interpretable ligand K-edge to decode the complex metal L-edge spectrum. A Born-Oppenheimer molecular dynamics simulation further captures thermally-driven transitions between side-on and end-on configurations, showing distinct spectral signatures, and identifies the O K-edge as a sensitive probe for Cu-O bond fluctuations. We establish a dual-edge protocol for decoding metal L-edge spectra and demonstrate the complementary power of static and dynamical simulation: the former offers a practical route to statistically averaged spectral trends, while the latter delivers explicit time-resolved insight into stochastic events. They provide a robust framework for mapping atomic-level electron flow in ultrafast X-ray studies of catalysis and energy science.

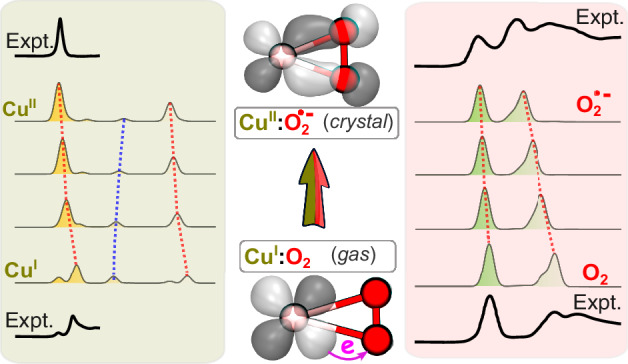

## Abstract (web summary)

While time-resolved X-ray spectroscopy offers insight into electron dynamics in chemical reactions, interpreting spectral data to identify transient intermediates and electron transfer mechanisms remains challenging. Here, the authors report a dual-edge strategy that simultaneously simulates O K-edge and Cu L-edge X-ray absorption spectra for the paradigmatic CuO2+ system, reporting a protocol for decoding metal L-edge spectra and mapping atomic-level electron flow in ultrafast X-ray studies.

## Introduction

Tracking the dynamic evolution of oxidation states (OS) during chemical reactions represents a central challenge in ultrafast chemistry. As a fundamental descriptor of electron distribution, the OS framework provides a rigorous basis for understanding redox processes, bridging formal chemical bookkeeping with the underlying quantum mechanical electron density^1–4^. This interplay between electronic structure and nuclear geometry manifests in the bidirectional coupling where “structural changes-induced OS changes”^5,6^ and “oxidation-induced structural changes”^7,8^ are two sides of the same coin.

In transition metal complexes, a particularly subtle yet vital process occurs on the electronic ground state: structurally-driven electron transfer. Unlike the instantaneous vertical valence transitions probed by optical spectroscopy (e.g., metal-to-ligand and ligand-to-metal charge transfer), this ground-state electron flow represents a continuous reorganization of the chemical bond. Examples include metal-to-ligand electron transfer (MLET) triggered by vibrational coherences or thermal fluctuations. Deciphering these correlated electronic-nuclear motions on the ground-state potential energy surface (PES) is essential for understanding the intrinsic mechanisms of catalysis, energy conversion, and metalloenzyme function.

Modern time-resolved X-ray spectroscopy, enabled by X-ray free electron lasers (XFELs)^9–12^ and high harmonic generation (HHG) table-top systems^13–18^, provides powerful access to these dynamics via techniques like transient X-ray absorption (TXAS)^19^ and photoelectron (TXPS) spectroscopy^20^, and time-resolved resonant inelastic X-ray scattering^21^. However, interpreting these complex spectral measurements requires theoretical frameworks that can accurately and efficiently capture the ultrafast dynamics, which can be modeled via static or dynamical approaches^22^.

In the static framework, computing theoretical TXAS spectra against a reaction coordinate *ξ*, *S*_TXAS_(*ω*, *ξ*), has proven to be an economical and effective strategy. By synthesizing these static maps with experimental delay-time data, *S*_TXAS_(*ω*, *t*), one can resolve the structural drivers of spectral evolution–a method successfully applied to K-edge studies of photochemical^22–24^ and thermal^25,26^ reactions. However, extending this rigor to transition metal L-edges is substantially more demanding, as accurate treatment requires accounting for spin-orbit (SO) coupling, multiplet effects, and dynamic correlation^27–30^. This presents unique challenges for constructing smooth PESs and tracking continuous spectral evolution.

This challenge is further magnified in dynamical approaches, which must balance the dual burden of trajectory simulations and high-fidelity X-ray spectral calculations. This requirement exemplifies the intrinsic trade-off between spectroscopic accuracy and dynamical feasibility in transition metal complexes^31^. Indeed, while non-adiabatic dynamics (e.g., surface hopping) have been coupled with X-ray scattering to elucidate ultrafast spin-crossover^32^, the analogous task for L-edge spectra remains an open challenge. Accurately simulating continuous, peak-resolved L-edge spectra along trajectories requires treating SO coupling and multiplet effects across a vast ensemble of electronic states–a computational cost far exceeding that for scattering signals, which are primarily determined by nuclear pair distribution functions. Consequently, most time-resolved studies of metal L-edge spectra have been limited to one or a few discrete, representative geometries^21,33^. To date, this discrete-geometry approach has also been the predominant strategy for interpreting time-resolved metal K-edge spectra^34^. Even for K-edge data, despite methodological advances–including spectra computed from static geometries displaced along normal modes^35^ and multi-configurational time-dependent Hartree (MCTDH) approaches^36^ coupled with vibronic Hamiltonians^37,38^–a continuous, peak-resolved analysis that tracks the evolution of electronic transitions and their underlying orbital character across a sequence of snapshots remains unavailable.

**Fig. 1:**
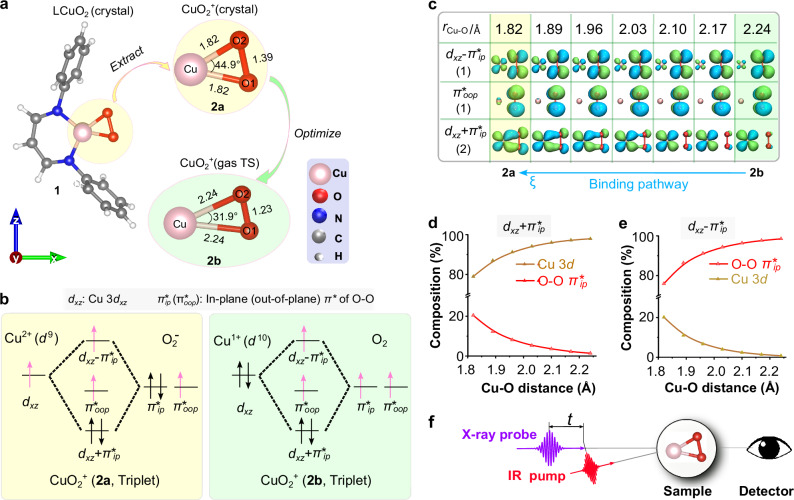
Molecular structures, electronic structure, and proposed TXAS experimental scheme. **a** Structures of Cu-O_2_ complexes under study: **1** (LCuO_2_), **2a** and **2b** (${{{{\rm{CuO}}}}}_{2}^{+}$). Coordinates of **1** were taken from the crystal structure^64^ with distant groups truncated by hydrogens. **2a,** ${{{{\rm{CuO}}}}}_{2}^{+}$ unit extracted from **1** with ligand L^−^ removed. **2b**, gas-phase optimized transition state geometry of **2a** by B3LYP (triplet state). Bond lengths (in Å) and the O-Cu-O angle (in ^∘^) are labeled. **b** Schematic energy level diagram for the three key orbitals^53,54^ of ${{{{\rm{CuO}}}}}_{2}^{+}$ at its triplet ground electronic state with Cu(II):${{{{\rm{O}}}}}_{2}^{-}$ (left) or Cu(I):O_2_ (right) configurations. **c** Evolution of the three key canonical MOs (Cu 3${d}_{xz}+{{{{\rm{O}}}}}_{2}\,{\pi }_{{{{\rm{ip}}}}}^{* }$, ${\pi }_{{{{\rm{oop}}}}}^{* }$, and Cu 3*d*_*xz*_--${{{{\rm{O}}}}}_{2}\,{\pi }_{{{{\rm{ip}}}}}^{* }$) at varying *r*_Cu-O_ distances (in Å). Integer occupation numbers are included in parentheses (detailed real numbers at each geometry provided in Table S3). Compositions of Cu 3*d* and ${{{{\rm{O}}}}}_{2}{\pi }_{{{{\rm{ip}}}}}^{* }$ in (**d**) Cu 3${d}_{xz}+{{{{\rm{O}}}}}_{2}\,{\pi }_{{{{\rm{ip}}}}}^{* }$ and (**e**) Cu 3*d*_*xz*_--${{{{\rm{O}}}}}_{2}\,{\pi }_{{{{\rm{ip}}}}}^{* }$ in RASSCF calculations with varying *r*_Cu-O_. **f** IR pump--X-ray probe setup proposed to detect the binding: a resonant IR pulse initiates vibrational coherences (*t* = 0) on the ground-state PES, while subsequent X-ray pulses track the transient electronic and structural reorganization.

Herein, we address these challenges through a theoretical investigation of the side-on ${{{{\rm{CuO}}}}}_{2}^{+}$ cation (Fig. 1a)–a paradigmatic system exhibiting variable valences (Cu^I/II/III^) without spin-state complications common in iron systems. As the fundamental Cu-O_2_ motif in key copper oxygenases (e.g., PHM, D*β*M)^39,40^ and synthetic catalysts^41,42^, this cation provides an ideal platform to isolate intrinsic metal-dioxygen interactions from ligand-specific effects. While enzymatic environments stabilize the side-on geometry via steric constraints^43^, the dynamic interconversion between coordination modes and its manifestation in transient spectra remain poorly understood. Crucially, the captured dynamics depend on the pump wavelength. While ultraviolet-visible pumps initiate dynamics on excited electronic states^19,44^, infrared (IR) pumps resonantly excite vibrational coherences on the electronic ground state^45–47^. Given that thermally driven ET in ${{{{\rm{CuO}}}}}_{2}^{+}$ occurs on the ground state, we propose an IR pump–X-ray probe setup (Fig. 1f), where an IR pulse initiates vibrational coherences on the PES. While the X-ray probe inherently involves transitions to core-excited manifolds, the observed spectral evolution acts as a sensitive mapping of the underlying ground-state orbital reorganization and covalent mixing (Fig. 1b–e).

The goal of this study is to investigate the OS transition and spectral responses to different Cu-O_2_ interactions by computing soft TXAS spectra of ${{{{\rm{CuO}}}}}_{2}^{+}$ at both the Cu L_2,3_- and O K-edges. This dual-edge framework leverages the metal L-edge’s sensitivity to 3*d* features and the ligand K-edge’s direct probe of ligand environments. The synergistic power of such dual-edge (or multi-edge) X-ray spectroscopy has been well established for elucidating complex electronic and structural dynamics^23,34,48,49^. The central advance of this work is the strategic use of the more interpretable O K-edge as a key to decode the intricate features of the Cu L_2,3_-edge. We combine two complementary approaches: (1) snapshots along static PESs to provide an efficient, well-defined mapping of statistically averaged spectral evolution; and (2) room-temperature Born-Oppenheimer molecular dynamics (BOMD) simulations to capture explicit time-dependence and stochastic structural fluctuations inherent to real systems. This integrated framework allows us to decode how geometric changes drive electronic state evolution–specifically, whether and how symmetric Cu-O bond shortening triggers MLET. Furthermore, it enables us to resolve the unique signatures of the side-on motif and its dynamic transition to end-on configurations, providing essential benchmarks for future ultrafast X-ray experiments in bioinorganic chemistry and energy science.

## Results and discussion

### Ground-state chemical and electronic structures

The ${{{{\rm{CuO}}}}}_{2}^{+}$ cation (**2**) structures are derived from the crystal structure of the enzyme model complex [Cu(O_2_)(*β*-diketiminate)]^50^ (LCuO_2_, **1**) by removing its nitrogen-containing ligand L^−^ (Fig. 1a). **1** was identified as having Cu(III):peroxide (Cu(III):${{{{\rm{O}}}}}_{2}^{2-}$) character combining Cu K- and L-edge XAS, valence bond configuration interaction simulations, and broken-symmetry density functional theory (DFT) calculations^51^. Our recent restricted active-space second-order perturbation theory (RASPT2) study^30^ successfully reproduced the experimental Cu L-edge XAS, and demonstrated that minor Cu-O_2_ distance fluctuations ( ~ 0.03 Å) sensitively influence the spectral features^30^. However, extending such high-level analysis across a broader configuration space to comprehensively characterize the binding dynamics faces technical challenges: maintaining a consistent and sufficiently large active space across all geometries to ensure smooth PESs and spectral evolution. To isolate the intrinsic nature of Cu-O_2_ bonding, we utilize the simplified model **2**. This reduction in computational complexity enables a more extensive exploration of the configuration space, providing fundamental insights that will inform future studies of biologically relevant systems featuring diverse ligand environments.

The configuration space of ${{{{\rm{CuO}}}}}_{2}^{+}$ spans from the crystal-phase geometry (**2a,** *r*_Cu-O_=1.82 Å, *r*_O-O_=1.39 Å) and its gas-phase optimized structure (**2b,** *r*_Cu-O_=2.24 Å, *r*_O-O_=1.23 Å) (Fig. 1a). The significant 0.42 Å Cu-O elongation and 0.16 Å O-O contraction upon relaxation reflect significant electronic reorganization. Internal reaction coordinate (IRC) calculations confirm **2b** as a transition state (TS) connecting two equivalent end-on minima on the intrinsic gas-phase PES (Supplementary Fig. S1). However, as the side-on motif is frequently stabilized in enzymatic active sites by steric constraints^27,43^, our study focuses on this enzyme-constrained pathway, modeling the symmetric Cu-O shortening from **2b** to **2a** via an interpolated path. Based on experimental O-O bond lengths (${{{{\rm{O}}}}}_{2}^{2-}$:1.49 Å, ${{{{\rm{O}}}}}_{2}^{-}$:1.34 Å, O_2_:1.21 Å, ${{{{\rm{O}}}}}_{2}^{+}$:1.12 Å)^52^, we preliminarily assign **2a** as Cu(II):${{{{\rm{O}}}}}_{2}^{-}$ and **2b** as Cu(I):O_2_, assignments to be rigorously tested via XAS.

A triplet ground state was identified at both geometries (Supplementary Note 1, Tables S1-S2). The singlet-triplet splitting (Δ*E*_ST_ ≡ *E*_S_ − *E*_T_) increases from 0.66 eV (**2a**) to 0.92 eV (**2b**) at the RASPT2 level, with B3LYP-DFT showing a similar trend (Supplementary Table S2). The enhanced splitting at longer Cu-O distances reflect the system’s approach toward isolated O_2_ character. Three key molecular orbitals (MOs) govern the electronic structure^53,54^: the bonding (${d}_{xz}+{\pi }_{{{{\rm{ip}}}}}^{* }$) and antibonding (${d}_{xz}-{\pi }_{{{{\rm{ip}}}}}^{* }$) combinations of Cu 3*d*_*xz*_ and ${{{{\rm{O}}}}}_{2}{\pi }_{{{{\rm{ip}}}}}^{* }$ (in-plane) orbitals, and the primarily O_2_-based ${\pi }_{{{{\rm{oop}}}}}^{* }$ (out-of-plane) orbital, with minor Cu 3*d*_*yz*_ contribution (Fig. 1b). Restricted active space self-consistent field (RASSCF) calculations confirm the expected single occupation (1.04-1.05) of both ${d}_{xz}-{\pi }_{{{{\rm{ip}}}}}^{* }$ and ${\pi }_{{{{\rm{oop}}}}}^{* }$ orbitals (Supplementary Note 2B, Table S3), consistent with DFT frontier orbitals (Supplementary Fig. S2a).

Using *r*_Cu-O_ as an effective reaction coordinate (*ξ*), we track orbital evolution along this binding pathway (**2b**  → **2a**). While orbital occupations remain stable (Supplementary Table S3), significant covalent mixing develops in the ${d}_{xz}\pm {\pi }_{{{{\rm{ip}}}}}^{* }$ pair (Fig. 1c-e): the Cu contribution to ${d}_{xz}+{\pi }_{{{{\rm{ip}}}}}^{* }$ decreases from  ~ 100% to 80%; while its contribution to ${d}_{xz}-{\pi }_{{{{\rm{ip}}}}}^{* }$ increases from  ~ 0% to 20%. This reveals competing electron transfer processes: (1) MLET via ${d}_{xz}+{\pi }_{{{{\rm{ip}}}}}^{* }$ stabilization; (2) Ligand-to-metal ET (LMET) through ${d}_{xz}-{\pi }_{{{{\rm{ip}}}}}^{* }$ destabilization. Net atomic charge analysis confirms dominant MLET character, with Cu charge increasing from +1.0*e* (**2b**) to +1.2*e* (**2a**) (Supplementary Note 2C, Fig. S3). These systematic electronic structure changes consistently support an OS transition from Cu(I) in **2b** to Cu(II) in **2a**, setting a clear target for validation by time-resolved X-ray spectroscopy. (See Supplementary Note 2D for vibrational analysis relevant to the proposed IR-pump scheme.)

### Cu 2*p* XAS spectral fingerprints of Cu(I), Cu(II), and Cu(III)

**Fig. 2:**
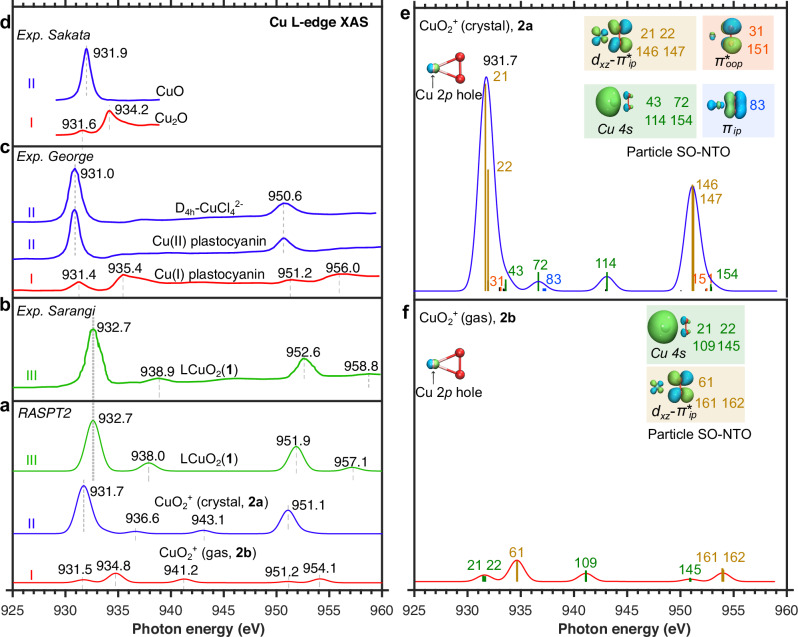
Simulated and experimental Cu L_2,3_-edge XAS of Cu-O_2_ complexes. **a** Cu L_2,3_-edge XAS spectra of LCuO_2_ (**1**) and two ${{{{\rm{CuO}}}}}_{2}^{+}$ structures (**2a** and **2b**) simulated by RASPT2. **b** Experimental spectrum^51^ of **1** is recaptured on the top for comparison. **c**, **d** Recapture of experimental spectra of other Cu complexes with known OSs^55,56^. Stick spectra of (**e**) **2a** and (**f**) **2b** are shown with dominant transitions marked by indexes of final SO states (all initially from SO state 1). Stick spectra of the same type are marked with the same color. The inset gives the SO-NTO analysis for labeled peaks. All particle NTOs are shown. The hole orbitals are all Cu 2p orbitals with different orientations; only one representative is displayed for clarity.

Interpreting future transient signals necessitates a firm understanding of the static endpoints, **2a** and **2b**. As a first step, we compute steady-state Cu L_2,3_-edge spectra for LCuO_2_ (**1**) and ${{{{\rm{CuO}}}}}_{2}^{+}$ (**2a, 2b**) and benchmark them against experimental references with well-known OSs (Fig. 2a-d). These references include Cu(III) (**1**^51^, Cu(II) (CuO^55^, D_4h_-${{{{\rm{CuCl}}}}}_{4}^{2-}$^56^, and Cu(II) plastocyanin^56^), and Cu(I) (Cu_2_O^55^ and Cu(I) plastocyanin^56^). Spectra of complexes with the same OS show similar energy and profile. Especially, the pronounced Cu(I) signature allows immediate identification in **2b**, whereas Cu(II)/Cu(III) spectra have similar shapes but differ mainly in energy positions.

While multiconfigurational methods exhibit systematic deviations of several eV in absolute soft X-ray transition energies^23,30,57^, we established a robust calibration protocol. By aligning the first peak of **2b** to the experimental Cu(I) reference (931.5 eV; averaged from 931.4 eV of Cu(I) plastocyanin^56^ and 931.6 eV of Cu_2_O^55^), we determined a -5.9 eV energy correction. We assume the same shift for all ${{{{\rm{CuO}}}}}_{2}^{+}$ structures. As a result, the first peak of **2a** appears at 931.7 eV, intermediate between 931.9 eV (CuO) and 931.0 eV (D_4h_-${{{{\rm{CuCl}}}}}_{4}^{2-}$ and Cu(II) plastocyanin), indicating a Cu(II) assignment. The 0.2 eV relative shift between **2a** and **2b** matches the 0.3 eV difference between CuO and Cu_2_O references^55^, further validating our OS assignments.

### Decoding the XAS spectrum of ${{{{\rm{CuO}}}}}_{2}^{+}$: gas- vs. crystal-phase assignments

To elucidate the electronic origins of the Cu *L*_2,3_-edge features, we performed spin-orbit natural transition orbital (SO-NTO)^58^ calculations for **2a** and **2b** (Fig. 1e). Atomic orbital decomposition of the final states (Supplementary Note 2E, Table S4) reveals four distinct transition classes in **2a**. The dominant features arise from (i) *d*_*xz*_-${\pi }_{{{{\rm{ip}}}}}^{* }$ antibonding orbitals, producing main transitions at 931.7 eV (L_3_, states 21-22) and 951.1 eV (L_2_, states 146-147). These are accompanied by three types of minor transitions: (ii) Cu 4*s*-hybridized states (with minor contributions from Cu 3*d* and O 2*p*) most evident at 936.6 eV (state 72) and 943.1 eV (state 114); (iii) O-O ${\pi }_{{{{\rm{oop}}}}}^{* }$ contributions at 933.0 eV (state 31) and 952.4 eV (state 151) embedded within main peaks; and (iv) a 3*d*-*π*_ip_ antibonding transition at 937.1 eV (state 83) that overlaps with primary features.

The spectral signature simplifies dramatically in **2b** (Fig. 2f), where we observe only two transition types: (i) the characteristic ${d}_{xz}-{\pi }_{{{{\rm{ip}}}}}^{* }$ orbitals generating peaks at 934.8 and 954.0 eV, and (ii) Cu 4*s* contributions at 931.5, 941.1, and 951.0 eV. This simplification correlates with the Cu(I) character identified in our spectra, where the disappearance of ${\pi }_{{{{\rm{oop}}}}}^{* }$ features reflects weakened Cu 3*d*_*yz*_–O_2_ interactions at longer bond lengths. These findings establish that the side-on binding process not only shifts transition energies but fundamentally alters the density of states available for X-ray excitation.

### Tracking the dioxygen reduction: O 1*s* TXAS along the Cu–O_2_ binding pathway

**Fig. 3:**
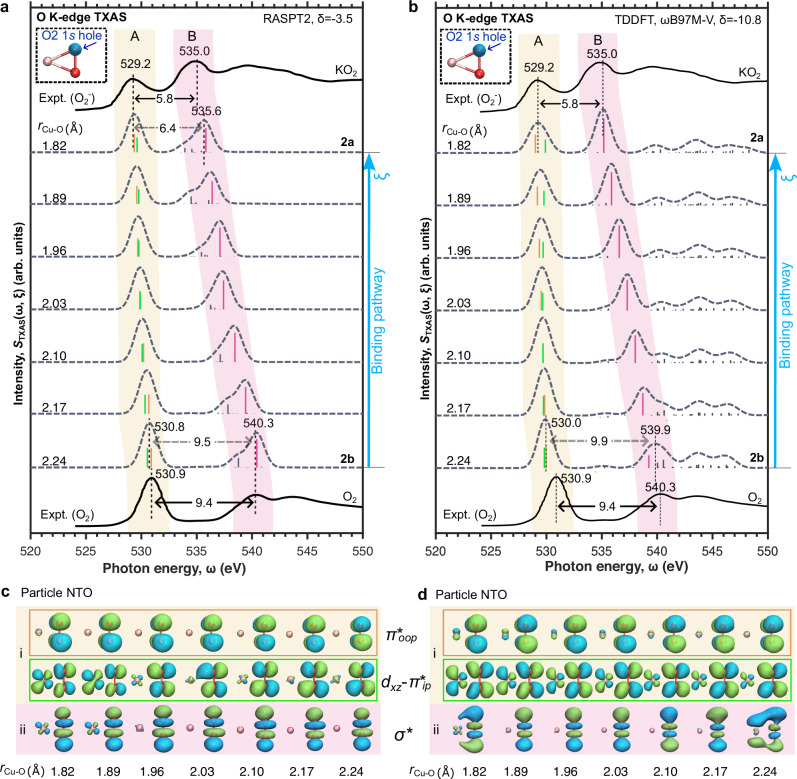
O K-edge TXAS and NTO evolution along the Cu-O bond stretching pathway. Simulated O 1*s* TXAS spectra of ${{{{\rm{CuO}}}}}_{2}^{+}$ along the Cu-O bond stretching pathway by using the (**a**) RASPT2 and (**b**) TDDFT/TDA methods. Comparisons were made to experiments^61^ of KO_2_ and O_2_ crystals. Inset, (**a**) hole SO-NTO and (**b**) hole NTO at an ordinary snapshot showing our calculation based on a localized core hole picture (green color indicates the MO is localized on O2). Evolution of the (**c**) particle SO-NTOs by RASPT2 and (**d**) particle NTOs by TDDFT/TDA corresponding to peaks A and B. Note that peak A in panels a-b is assigned from two stick transitions, distinguished by colors (green and orange), and the corresponding particle NTOs were enclosed in frames with consistent colors.

Figure 3 a-b displays the simulated O 1*s* TXAS spectra of ${{{{\rm{CuO}}}}}_{2}^{+}$ along the binding pathway, obtained by RASPT2^59^ and time-dependent DFT with the Tamm-Dancoff approximation^60^ (TDDFT/TDA). Both methods yield consistent spectral evolution. At structures **2b** and **2a**, the ${{{{\rm{CuO}}}}}_{2}^{+}$ spectra match experimental spectra of O_2_ and KO_2_ crystals^61^ (with clearly identified oxygen OS of 0 and  − 1), confirming their oxygen OS of 0 and  − 1, respectively. The O_2_ spectrum exhibits a strong π^*^ peak A at 530.9 eV, a medium σ^*^ peak B at 540.3 eV, and a broad σ^*^ peak at 543.9 eV; while KO_2_ shows analogous peaks redshifted to 529.2, 535.0, and 539.9 eV. The experimental π^*^-σ^*^ separation between peaks A and B (Δ) decreases by 3.6 eV (9.4  → 5.8 eV) upon OS change (0  →  − 1). RASPT2 produced a smaller reduction of 3.1 eV (9.5  → 6.4 eV), while TDDFT/TDA produced a larger change of 4.1 eV (9.9  → 5.8 eV). The complementary under- and overestimation by the two methods provides a comprehensive perspective that bounds the experimental Δ evolution. The comparison with O_2_/KO_2_ experiments indicates an OS change of oxygen from 0 to -1 in ${{{{\rm{CuO}}}}}_{2}^{+}$ along the Cu-O_2_ binding pathway, with a corresponding change in the OS of copper from +1 to +2. The redshifts of peaks A (1.7 eV) and B (5.3 eV) in the experiment are reproduced by RASPT2 (1.6 and 4.7 eV) and TDDFT/TDA (0.8 and 4.9 eV). These shifts reflect MLET^62^ and the increasing Cu OS.

Figure 3 c–d analyzes transitions via NTOs. Peak A originates from ${d}_{xz}-{\pi }_{{{{\rm{ip}}}}}^{* }$ (green) and ${\pi }_{{{{\rm{oop}}}}}^{* }$ (orange) states with comparable intensities at **2b**, indicating the degeneracy in ${\pi }_{{{{\rm{ip}}}}}^{* }$ (with minimal *d*_*xz*_ contributions) and ${\pi }_{{{{\rm{oop}}}}}^{* }$ orbitals. Progressing along the binding pathway, the intensity of the ${\pi }_{{{{\rm{oop}}}}}^{* }$ transition gradually increases over the intensity of the ${d}_{xz}-{\pi }_{{{{\rm{ip}}}}}^{* }$ transition, due to the increasing *d*_*xz*_ components. Both methods exhibit a reversal in the energy ordering of the two states that constitute peak A. The physical origin of this reversal is core-hole relaxation, which we examine via DFT-based core-hole analysis (Supplementary Note 2A, Fig. S4). TDDFT/TDA yields a larger splitting than RASPT2 at **2a**. For peak B (O1*s* → σ^*^), RASPT2 reveals clearer (i.e., without the entanglement of numerous weak transitions) evolution but lacks high-energy states due to limited state coverage; including more states in the RASPT2 calculation would require careful balancing of state weights^63^. TDDFT/TDA, including more states, better reproduces the broad high-energy features.

### Tracking the copper oxidation: Cu 2*p* TXAS along the Cu–O_2_ binding pathway

**Fig. 4:**
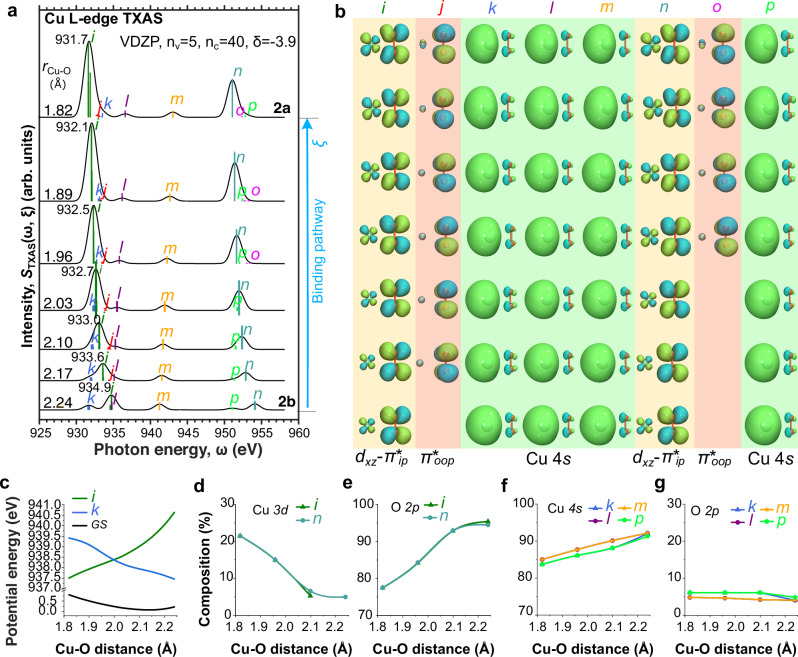
Cu L_2,3_-edge TXAS and NTO evolution along the Cu-O bond stretching pathway. **a** Simulated Cu L_2,3_-edge TXAS spectra of ${{{{\rm{CuO}}}}}_{2}^{+}$ by RASPT2 along the Cu-O bond stretching pathway. Snapshot structures are interpolated between **2a** and **2b** (see structures in Fig. 1a). Each curve is labeled by the Cu-O distance in Å. Major stick peaks are labeled by *i*, *j*, ⋯  , *n*. **b** Evolution of individual peaks interpreted by SO-NTOs (only particle orbitals are shown). Colored shading distinguishes three orbital types: ${d}_{xz}-{\pi }_{{{{\rm{ip}}}}}^{* }$ (orange), ${\pi }_{{{{\rm{oop}}}}}^{* }$ (pink), and Cu 4*s* (green). **c** Potential energy curves for the ground state and low-lying Cu 2*p* core-excited states corresponding to peaks *i* and *k*. Composition of particle SO-NTOs for transitions *i* and *n* as a function of Cu--O distance: (**d**) Cu 3*d* and (**e**) O 2*p* orbital contributions. SO-NTO particle orbital compositions for transitions *k*, *l*, *m*, and *p*: (**f**) Cu 4*s* and (**g**) O 2*p* contributions. Numerical orbital composition data are provided in Supplementary Table S5.

The Cu L_2,3_-edge spectral evolution along the binding pathway (Fig. 4a) directly evidences the Cu oxidation state transition, complementing the O K-edge analysis. As *r*_Cu-O_ shortens from 2.24 to 1.82 Å, the L_3_ and L_2_ main peaks (*i* and *n*) intensify. In the molecular orbital picture, this enhancement arises from increased Cu 3*d* character in the particle SO-NTOs (Fig. 4d), which strengthens the 2*p* → 3*d* transition dipoles. This description aligns with the atomic orbital view: the system evolves from a Cu(I) 3*d*^10^ configuration at **2b** toward a Cu(II) 3*d*^9^ configuration at **2a**. The transition from a fully filled to a partially filled 3*d* shell enhances excitation intensity because the 3*d*^9^ configuration allows the excited electron to occupy an orbital vacancy, creating a stable 3*d*^10^ final state with favorable electron correlation. The dominant transitions *i* (934.9  → 931.6 eV) and *n* (954.2  → 951.0 eV) exhibit significant redshifts. Figure 4b traces this progression through particle SO-NTOs, revealing the redshift mechanism: increased Cu 3*d* and diminished O 2*p* character in the excited states (Fig. 4d-e); quantitative data in Supplementary Table S5. Similar trends of redshift are found for weak peaks *j* and *o*.

Conversely, peaks *k*, *l*, *m*, and *p* exhibit noticeable blueshifts while maintaining stable intensities. Their features (Fig. 4b) correlate with a progressive decline in Cu 4*s* proportions (92%  → 85%, Fig. 4f), alongside minor changes in O 2*p* (4.0–6.1%, Fig. 4g) and Cu 3*d* contributions (<0.6%, Supplementary Table S5). Shoulder peaks *k* and *p* blueshift from the low- to high-energy sides of their main peaks *i* and *n*, intersecting at 2.01 Å along the pathway (Fig. 4c). Despite this energy shift, their intensities remain constant across all configurations, resulting in comparable shoulder-to-main peak ratios at **2b** that merge into the main peak at **2a**. This NTO analysis collectively elucidates the electronic structural origins of spectral evolution.

### Tracking the energy and structural evolution along a BOMD trajectory

Complementary to the static analysis, we performed BOMD simulations at the RASSCF level. This single-trajectory approach is designed to capture spectral responses to specific stochastic events, providing a dynamic counterpoint to the symmetric static method. The system was propagated for 1.45 ps at 300 K using a Nos’e-Hoover thermostat. While the MD simulation required only 18 hours (dual Intel Xeon Platinum 8171M CPUs, 52 cores), each subsequent X-ray spectral calculation per snapshot demanded roughly 6 hours (8 cores), highlighting the computational investment required for high-fidelity time-resolved spectroscopy.

**Fig. 5:**
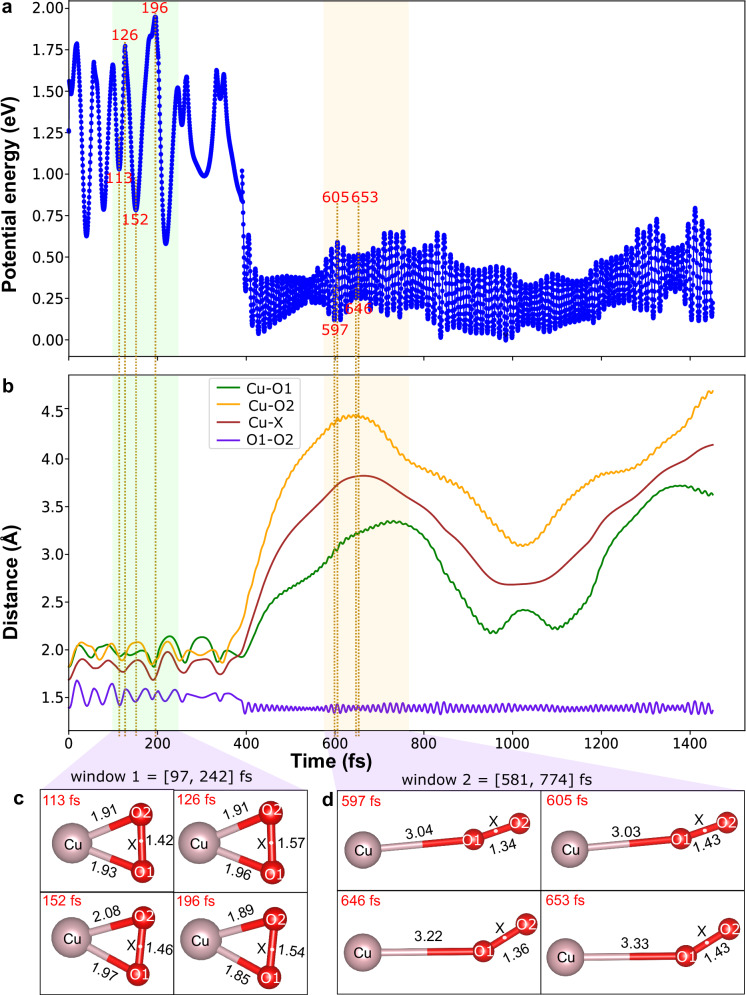
Energetic and structural evolution for the BOMD trajectory. **a** Potential energy (RASPT2) along the 1.45-ps trajectory. **b** Key interatomic distances: Cu-O1, Cu-O2, and O1-O2. The simulation separates into two dynamical regimes around 400 fs, characterized by dominant side-on and end-on geometries. The shaded regions (97-242 fs and 581-774 fs) denote the time windows selected for spectral analysis in Fig. 6a and 6b. **c**, **d** Representative snapshots from each regime with key geometric parameters labeled.

Figure 5 a,b depicts the energetic and structural evolution. Notably, the two Cu-O distances exhibit distinct dynamic behavior. A marked decrease in total energy after  ~ 400 fs correlates with an overall elongation of the Cu-O bonds, signaling a structural transition from a predominantly side-on to the thermodynamically preferred gas-phase end-on configuration. The initial phase (0-400 fs) exhibits pronounced oscillations where Cu-O1 and Cu-O2 distances recurrently exchange relative magnitudes, accompanied by significant O1-O2 vibrations (Supplementary Movie 1). Beyond this point, the geometry stabilizes into a highly asymmetric state where one Cu-O distance (Cu-O2) remains consistently larger, reflecting the end-on binding preference in the absence of environmental constraints. Correspondingly, the O1-O2 distance exhibits markedly reduced oscillatory amplitude.

This observed evolution carries an important implication: the symmetric side-on geometry, while central to enzymatic function in constrained environments such as the active sites of copper oxygenases, is metastable for the isolated cation. Its spontaneous interconversion to the end-on configuration yields distinct spectroscopic signatures that we now examine in detail.

### Tracking the spectral evolution along the BOMD trajectory

**Fig. 6:**
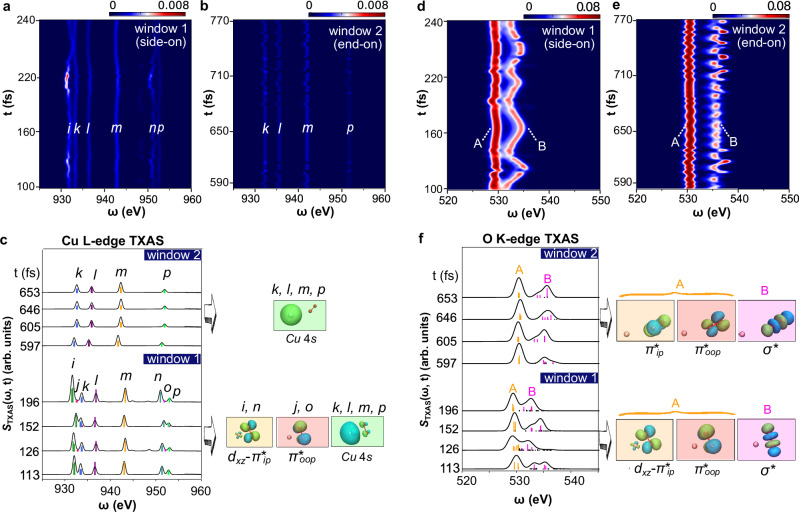
TXAS spectra computed from two windows in the BOMD trajectory. **a**–**c** Cu L_2,3_-edge and (**d**–**f**) O K-edge TXAS spectral evolution from BOMD trajectory analysis. Cu L_2,3_-edge spectra for (**a**) window 1 (side-on, 97--242 fs) and (**b**) window 2 (end-on, 581--774 fs). **c** Corresponding one-dimensional (1D) spectral cuts, with individual stick transitions labeled (labels are consistent throughout). Particle spin-orbit NTOs for labeled peaks are summarized to the right. **e** O K-edge spectra for the same two windows. **f** Corresponding 1D cuts, with major stick transitions within peaks A/B are color-coded by final-state orbital character. Particle NTOs are displayed to the right. In (**c**) and (**f**), orbital categories are distinguished by shaded colors.

We analyzed two distinct time windows for both edges: window 1 (97–242 fs, side-on) and window 2 (581–774 fs, end-on). At the Cu L-edge (Fig. 6a-c), window 1 exhibits a double-peak structure (*i* and *k*) in the L_3_ region, spanning 931.5–933.6 eV. As the Cu–O distances in this window fluctuate between 1.8 and 2.2 Å, a range that fortuitously overlaps with the equilibrium distances of the static models **2a** and **2b**, the spectra within this window can be viewed as a dynamic modulation of the spectral signatures of **2a** and **2b**. SO-NTO analysis reveals these features originate from excitations to *d*_*xz*_–${\pi }_{{{{\rm{ip}}}}}^{* }$, ${\pi }_{{{{\rm{oop}}}}}^{* }$, and Cu 4*s* orbitals. Conversely, window 2 (end-on) simplifies to a single feature (*k*) in the L_3_ region spanning a narrower energy range of 931.0–932.1 eV. SO-NTO analysis indicates that the spectral intensity is dominated solely by Cu 2*p* → Cu 4*s* transitions, a signature of the asymmetric end-on geometry with single-oxygen coordination. The two windows exhibit markedly distinct spectral features, underscoring the high sensitivity of the Cu L-edge to the side-on versus end-on structural dichotomy.

The O K-edge (Fig. 6d-f) demonstrates even greater diagnostic power for discriminating between side-on and end-on geometries. The spectral intensity is also notably stronger than at the Cu L-edge. In window 1, the spectra are characterized by two distinct peaks, A and B, analogous to those observed for the static models **2a** and **2b** (see Fig. 3). The intense peak A centered within a narrow range of 527.8–530.1 eV corresponds to hybridized *d*_*xz*_–${\pi }_{{{{\rm{ip}}}}}^{* }$ and ${\pi }_{{{{\rm{oop}}}}}^{* }$ transitions. While the medium peak B oscillates substantially within a wide range of 530.2–536.1 eV. This oscillation directly correlates with the dynamic exchange of the Cu–O1 and Cu–O2 bond lengths, as peak B originates from O 1*s* → O–O σ^*^ transitions within the dioxygen moiety, making it a sensitive real-time probe of the Cu–O distance. In window 2, the spectra also display two features, peaks A and B, but with altered electronic origins. Peak A derives from O 1$s\to {\pi }_{{{{\rm{ip}}}}}^{* }$ and ${\pi }_{{{{\rm{oop}}}}}^{* }$ transitions, with diminished Cu 3*d* contribution. Peak B exhibits milder oscillations in intensity and energy, consistent with the geometric stabilization of the end-on configuration where only the O1 atom maintains a persistent bond to the copper center.

Collectively, this BOMD analysis validates that the distinct spectral fingerprints identified from static geometries remain robust under dynamic conditions. It crucially highlights the O K-edge, specifically the O–O σ^*^ feature, as a superior real-time structural probe for tracking metal-ligand bond fluctuations–complementing and clarifying the information from the more complex Cu L-edge.

## Conclusions and outlook

We have demonstrated a dual-edge computational spectroscopy framework that tracks coupled electron-nuclear dynamics in the paradigmatic ${{{{\rm{CuO}}}}}_{2}^{+}$ system. This framework strategically integrates static “snapshots along the PESs” with efficiency and practicability to obtain the statistical average and room-temperature BOMD simulations to see the stochastic events, bridging the gap between ideal reaction coordinates and stochastic structural fluctuations inherent to real systems, and providing a comprehensive picture to understand the reaction dynamics. By simultaneously simulating Cu *L*_2,3_-edge and O K-edge spectra, we decoded the electronic evolution from Cu(I):O_2_ to Cu(II):${{{{\rm{O}}}}}_{2}^{\bullet -}$ during the side-on binding process. Our analysis reveals that this oxidation state transition is driven by competing electron transfer mechanisms–specifically, a dominant MLET and a secondary LMET process.

The BOMD simulations provide a dynamic validation of our static models, highlighting a clear structural-spectral dichotomy: the side-on geometry is characterized by a diagnostic double-peak structure at the Cu *L*_3_-edge, while the thermodynamically preferred gas-phase end-on configuration yields a simplified spectrum dominated by Cu 2*p* → 4*s* transitions. Crucially, the O K-edge emerges as the superior structural reporter; the wide-range oscillation of the O–O σ^*^ feature directly tracks Cu–O bond fluctuations in real-time, providing a clearer diagnostic than the more complex metal-centered multiplets.

The synergistic use of ligand K-edge data to decode intricate metal L-edge features establishes a general protocol for interpreting ultrafast X-ray experiments. This atomistic tracking of electron flow is immediately applicable to a broad range of localized electronic systems, from single-atom catalysts (SACs) to metalloenzyme active sites in oxygen reduction and evolution reactions (ORR/OER). Looking forward, the integration of advanced methods like density matrix renormalization group (DMRG) with periodic embedding schemes will extend this dual-edge capability to complex functional materials, offering a clear pathway for simulating the next generation of time-resolved X-ray experiments.

## Methods

### Static approach

The bare ${{{{\rm{CuO}}}}}_{2}^{+}$ structure (**2a**) was derived from the crystal structure of **1**^64^ by ligand truncation. Gas-phase geometric relaxations were performed at singlet, triplet, and quintet states using DFT with the B3LYP functional in GAUSSIAN 09^65^, employing the 6-31G* basis set for O and the modified LanL2DZ basis set with optimized 4p functions^66^ and the LanL2DZ pseudo potential for Cu^67^. Both **2a** and the optimized structure **2b** were confirmed to have triplet ground states (Supplementary Note 1). Vibrational frequencies were computed at both geometries. Intermediate structures along the binding coordinate were generated by linear interpolation of Cartesian coordinates between **2a** and **2b**, without further geometry optimization. The transition-state nature of **2b** was verified by internal reaction coordinate (IRC) calculations (Supplementary Fig. S1).

Cu L_2,3_-edge XAS spectra were simulated using the OPENMOLCAS package^68,69^ via state-averaged RASSCF^70,71^ and multi-state RASPT2^59^ methods, with analysis mainly based on the RASPT2 results. For all ${{{{\rm{CuO}}}}}_{2}^{+}$ structures, we set *n*_v_ = 5 for valence and *n*_c_ = 40 for core-excited states, with eight active orbitals (Cu 3*d* and O2) in RAS2 (Supplementary Fig. S5) and three Cu 2*p* orbitals in RAS1. The choice of this active space and other parameters was carefully validated (Supplementary Note 3B). Scalar relativistic effects were included using a second-order Douglas-Kroll-Hess Hamiltonian and the ANO-RCC-VDZP basis set^72^. The core hole was treated via the highly excited states method^73^. To accelerate calculations, the Cholesky decomposition^74^ was applied to approximate two-electron integrals. A level shift of 1.0 a.u. was used for RASSCF convergence, and an imaginary shift of 0.3 a.u. was applied to mitigate intruder states during RASPT2 analyses. Transition dipole moments were calculated using the RAS state-interaction (RASSI) approach^75,76^. Additionally, RASPT2 calculations for **1** were performed with optimal parameters^30^ of *n*_v_ = 5, *n*_c_ = 18, and *n*_A_ = 6.

At the O 1*s* edge, spectra were computed by RASPT2 with identical parameters to the Cu L-edge calculations (*n*_v_ = 5, *n*_c_ = 40, *n*_A_ = 8), modifying only RAS1 to contain the Boys-localized^77,78^ O2 1*s* orbital. Validation was performed using TDDFT/TDA in Q-CHEM^79^ with the *ω*B97M-V functional^80^ and mixed basis sets (def2-QZVP for O2; def2-TZVP for O1/Cu)^81,82^, restricting excitations to the localized O2 1*s* orbital to maintain consistency with the RASPT2 treatment. Different basis sets were chosen only to meet the specific requirements of each methodological stage, and these choices do not compromise the accuracy or conclusions of our work (Supplementary Note 4).

All spectra were broadened using a Gaussian lineshape with a half-width at half-maximum (HWHM) of 0.8 eV^30^. Energy shifts of *δ*_1_ = − 3.9 eV for **1** (aligned to experiment^51^) and *δ*_2_ = − 5.9 eV for **2b** (matched to Cu(I) references^55,56^) were applied, with *δ*_2_ subsequently applied consistently for **2a** and all interpolated structures. SO-NTOs (Cu edge) and NTOs (O edge) were computed for major transitions.

### BOMD approach

BOMD simulation was performed at the RASSCF level in OPENMOLCAS starting from the **2a** geometry and its converged wavefunction as the initial guess. Parameters were set identical to static calculations. An NVT ensemble at 300 K was propagated for 1.45 ps (3000 steps, Δ*t* = 20 a.u.) using the velocity Verlet integrator^83^ and a Nosé–Hoover chain thermostat. The converged valence electronic wavefunction was archived at every step to provide a robust initial guess for subsequent core state calculations, ensuring stability and efficiency.

XAS were subsequently computed for two consecutive windows of snapshot geometries extracted from the trajectory with dominant side-on (window 1, 97–242 fs) and end-on (window 2, 581–774 fs) configurations. Spectra were computed every 5 steps, yielding 61 (81) snapshots for window 1 (2). For each snapshot, the archived valence wavefunction served as the starting point for the RASPT2 calculation of core-excited states at either the Cu 2*p* or O 1*s* edges.

To properly account for the dynamically evolving symmetry-breaking, we modified the active space for the O K-edge: while the static symmetric models used a single pre-localized O 1*s* orbital in RAS1, the dynamical framework included both O1 and O2 1*s* orbitals. This adaptation allows for the simultaneous description of core holes localized on either oxygen site, which is essential for capturing the spectral signature of an asymmetric ligand environment. NTO analysis confirms the initial core-hole states span configurations localized on O1, O2, and their coherent superpositions (Supplementary Fig. S6). All graphics were generated by combining Multiwfn^84^ and VMD^85^ (isovalue=0.05).

## Supplementary information

Transparent Peer Review file

Supplementary Information

Description of Additional Supplementary Files

Supplementary Movie 1

## Data Availability

The data that support the findings of this study, including Cartesian coordinates (**1**, **2a**, **2b**, and BOMD trajectory) and all simulated spectral data, are openly available in Zenodo at [10.5281/zenodo.18334837], with additional data available from the corresponding author upon reasonable request.

## References

[1] Jørgensen, C. K. *Oxidation Numbers and Oxidation States* (Springer: New York, 1969).

[2] Karen, P. Oxidation state, a long-standing issue!. *Angew. Chem. Int. Ed.* **54**, 4716–4726 (2015). 10.1002/anie.201407561 PMC4506524 25757151

[3] Jiang, L., Levchenko, S. V. & Rappe, A. M. Rigorous definition of oxidation states of ions in solids. *Phys. Rev. Lett.* **108**, 166403 (2012). 22680739 10.1103/PhysRevLett.108.166403

[4] Walsh, A., Sokol, A. A., Buckeridge, J., Scanlon, D. O. & Catlow, C. R. A. Oxidation states and ionicity. *Nat. Mater.* **17**, 958–964 (2018). 30275565 10.1038/s41563-018-0165-7

[5] Saiful, I. et al. Ligand rotation induced oxidation state change and spin appearance of the Bis(Phthalocyaninato)Cerium (CePc_2_) molecule on the Au(111) surface. *J. Phys. Chem. C.* **126**, 17152–17163 (2022).

[6] Domcke, W. & Yarkony, D. *Conical Intersections: Theory, Computation and Experiment* Vol. 17 (World Scientific, 2011).

[7] Falke, S. M. et al. Coherent ultrafast charge transfer in an organic photovoltaic blend. *Science* **344**, 1001–1005 (2014). 24876491 10.1126/science.1249771

[8] Chergui, M. Ultrafast photophysics of transition metal complexes. *Acc. Chem. Res.* **48**, 801–808 (2015). 25646968 10.1021/ar500358q

[9] Emma, P. et al. First lasing and operation of an ångstrom-wavelength free-electron laser. *Nat. Photonics* **4**, 641–647 (2010).

[10] Duris, J. et al. Tunable isolated attosecond x-ray pulses with gigawatt peak power from a free-electron laser. *Nat. Photonics* **14**, 30–36 (2020).

[11] Seddon, E. A. et al. Short-wavelength free-electron laser sources and science: a review. *Rep. Prog. Phys.* **80**, 115901 (2017). 29059048 10.1088/1361-6633/aa7cca

[12] Pellegrini, C., Marinelli, A. & Reiche, S. The physics of x-ray free-electron lasers. *Rev. Mod. Phys.* **88**, 15006 (2016).

[13] Johnson, A. S. et al. High-flux soft x-ray harmonic generation from ionization-shaped few-cycle laser pulses. *Sci. Adv.* **4**, eaar3761 (2018). 29756033 10.1126/sciadv.aar3761 PMC5947981

[14] Popmintchev, D. et al. Near- and extended-edge x-ray-absorption fine-structure spectroscopy using ultrafast coherent high-order harmonic supercontinua. *Phys. Rev. Lett.* **120**, 93002 (2018). 10.1103/PhysRevLett.120.093002 29547333

[15] Schmidt, C. et al. High-order harmonic source spanning up to the oxygen k-edge based on filamentation pulse compression. *Opt. Express* **26**, 11834 (2018). 29716100 10.1364/OE.26.011834

[16] Teichmann, S. M., Silva, F., Cousin, S. L., Hemmer, M. & Biegert, J. 0.5-keV soft x-ray attosecond continua. *Nat. Commun.* **7**, 11493 (2016). 27167525 10.1038/ncomms11493 PMC4865833

[17] Ishii, N. et al. Carrier-envelope phase-dependent high harmonic generation in the water window using few-cycle infrared pulses. *Nat. Commun.* **5**, 3331 (2014). 24535006 10.1038/ncomms4331 PMC3929802

[18] Popmintchev, T. et al. Bright coherent ultrahigh harmonics in the kev x-ray regime from mid-infrared femtosecond lasers. *Science* **336**, 1287–1291 (2012). 22679093 10.1126/science.1218497

[19] Huse, N. et al. Femtosecond soft x-ray spectroscopy of solvated transition-metal complexes: deciphering the interplay of electronic and structural dynamics. *J. Phys. Chem. Lett.* **2**, 880–884 (2011). 26295622 10.1021/jz200168m

[20] Mayer, D. et al. Following excited-state chemical shifts in molecular ultrafast x-ray photoelectron spectroscopy. *Nat. Commun.* **13**, 198 (2022). 35017539 10.1038/s41467-021-27908-y PMC8752854

[21] Wernet, P. et al. Orbital-specific mapping of the ligand exchange dynamics of Fe(CO)_5_ in solution. *Nature* **520**, 78–81 (2015). 25832405 10.1038/nature14296

[22] Hua, W. MCNOX: a code for computing and interpreting ultrafast nonlinear x-ray spectra of molecules at the multiconfigurational level. *Comput. Phys. Commun.* **296**, 109016 (2024).

[23] Hua, W., Mukamel, S. & Luo, Y. Transient x-ray absorption spectral fingerprints of the S_1_ dark state in Uracil. *J. Phys. Chem. Lett.* **10**, 7172–7178 (2019). 31625754 10.1021/acs.jpclett.9b02692

[24] List, N. H., Dempwolff, A. L., Dreuw, A., Norman, P. & Martínez, T. J. Probing competing relaxation pathways in malonaldehyde with transient x-ray absorption spectroscopy. *Chem. Sci.* **11**, 4180–4193 (2020). 34122881 10.1039/d0sc00840k PMC8152795

[25] Ge, G., Zhang, J.-R., Wang, S.-Y., Wei, M. & Hua, W. A QM/MM study on the x-ray spectra of organic proton transfer crystals of isonicotinamides. *J. Phys. Chem. C.* **126**, 15849–15863 (2022).

[26] Ge, G. et al. Mapping hydrogen positions along the proton transfer pathway in an organic crystal by computational x-ray spectra. *J. Phys. Chem. Lett.* **15**, 6051–6061 (2024). 38819966 10.1021/acs.jpclett.4c01133

[27] Solomon, E. I. et al. Copper active sites in biology. *Chem. Rev.* **114**, 3659–3853 (2014). 24588098 10.1021/cr400327t PMC4040215

[28] Kasper, J. M., Stetina, T. F., Jenkins, A. J. & Li, X. Ab initio methods for L-edge x-ray absorption spectroscopy. *Chem. Phys. Rev.* **1**, 11304 (2020).

[29] Lundberg, M. & Delcey, M. G. Multiconfigurational approach to x-ray spectroscopy of transition metal complexes. in *Transition Metals in Coordination Environments* (Broclawik, E., Borowski, T. & Radoń, M. eds.) vol. 29, 185–217 (Springer International Publishing, Cham, 2019).

[30] Wang, S.-Y., Zhang, J.-R., Guo, M. & Hua, W. Interpreting the Cu–O_2_ antibonding nature in two Cu–O_2_ complexes from Cu L-edge X-Ray Absorption Spectra. *Inorg. Chem.* **62**, 17115–17125 (2023). 37828769 10.1021/acs.inorgchem.3c01896

[31] Zobel, J. P. & González, L. The quest to simulate excited-state dynamics of transition metal complexes. *JACS Au* **1**, 1116–1140 (2021). 34467353 10.1021/jacsau.1c00252 PMC8397362

[32] Rozgonyi, T., Vankó, G. & Pápai, M. Branching mechanism of photoswitching in an Fe(II) polypyridyl complex explained by full singlet-triplet-quintet dynamics. *Commun. Chem.* **6**, 7 (2023). 36697805 10.1038/s42004-022-00796-z PMC9829715

[33] Ghodrati, N. et al. Identification of metal-centered excited states in cr (iii) complexes with time-resolved l-edge x-ray spectroscopy. *Chem. Sci.* **16**, 6307–6316 (2025). 40078607 10.1039/d4sc07625g PMC11895842

[34] Lim, H. et al. Excited state covalency, dynamics, and photochemistry of square planar Ni-thiolate complexes revealed by ultrafast x-ray absorption. *J. Am. Chem. Soc.* **147**, 7496–7506 (2025). 39993950 10.1021/jacs.4c16212

[35] Barlow, K. et al. Tracking nuclear motion in single-molecule magnets using femtosecond X-ray absorption spectroscopy. *Nat. Commun.* **15**, 4043 (2024). 38744877 10.1038/s41467-024-48411-0 PMC11094174

[36] Beck, M. The multiconfiguration time-dependent Hartree (MCTDH) method: a highly efficient algorithm for propagating wavepackets. *Phys. Rep.* **324**, 1–105 (2000).

[37] Penfold, T. J., Pápai, M., Rozgonyi, T., Møller, K. B. & Vankó, G. Probing spin–vibronic dynamics using femtosecond X-ray spectroscopy. *Faraday Discuss.* **194**, 731–746 (2016). 27711829 10.1039/c6fd00070c

[38] Katayama, T. et al. Tracking multiple components of a nuclear wavepacket in photoexcited Cu(I)-phenanthroline complex using ultrafast X-ray spectroscopy. *Nat. Commun.* **10**, 3606 (2019). 31399565 10.1038/s41467-019-11499-w PMC6689108

[39] Kulathila, R., Merkler, K. A., Merkler, D. J. & Kulathila, R. Enzymatic formation of C-terminal amides. *Nat. Prod. Rep.* **16**, 145–154 (1999). 10331284 10.1039/a801346b

[40] Prigge, S. T., Mains, R. E., Eipper, B. A. & Amzel, L. M. New insights into copper monooxygenases and peptide amidation: structure, mechanism and function. *Cell. Mol. Life Sci.* **57**, 1236–1259 (2000). 11028916 10.1007/PL00000763 PMC11146793

[41] Elwell, C. E. et al. Copper–oxygen complexes revisited: structures, spectroscopy, and reactivity. *Chem. Rev.* **117**, 2059–2107 (2017). 28103018 10.1021/acs.chemrev.6b00636 PMC5963733

[42] De Tovar, J. et al. Copper–oxygen adducts: new trends in characterization and properties towards C–H activation. *Chem. Sci.* **15**, 10308–10349 (2024). 38994420 10.1039/d4sc01762e PMC11234856

[43] Mirica, L. M., Ottenwaelder, X. & Stack, T. D. P. Structure and spectroscopy of copper-dioxygen complexes. *Chem. Rev.* **104**, 1013–1046 (2004). 14871148 10.1021/cr020632z

[44] Canton, S. E. et al. Visualizing the non-equilibrium dynamics of photoinduced intramolecular electron transfer with femtosecond x-ray pulses. *Nat. Commun.* **6**, 6359 (2015). 25727920 10.1038/ncomms7359 PMC4366532

[45] Felicíssimo, V. C., Guimarães, F. F., Gel’mukhanov, F., Cesar, A. & A&#ring;gren, H. The principles of infrared-x-ray pump-probe spectroscopy. Applications on proton transfer in core-ionized water dimers. *J. Chem. Phys.* **122**, 094319 (2005). 15836140 10.1063/1.1860312

[46] Gavrila, G. et al. Time-resolved x-ray absorption spectroscopy of infrared-laser-induced temperature jumps in liquid water. *Appl. Phys. A* **96**, 11–18 (2009).

[47] Liu, J.-C., Savchenko, V., Kimberg, V., Odelius, M. & Gel’mukhanov, F. Polarization-sensitive IR-pump–x-ray-probe spectroscopy. *Phys. Rev. A* **103**, 022829 (2021).

[48] Ross, A. D. et al. Measurement of coherent vibrational dynamics with X-ray Transient Absorption Spectroscopy simultaneously at the Carbon K- and Chlorine L2,3- edges. *Commun. Phys.* **7**, 304 (2024). 39281307 10.1038/s42005-024-01794-4 PMC11399099

[49] Lätsch, L. et al. Tracking coordination environment and reaction intermediates in homogeneous and heterogeneous epoxidation catalysts via Ti L_2,3_ -edge near-edge x-ray absorption fine structures. *J. Am. Chem. Soc.* **146**, 7456–7466 (2024). 38447178 10.1021/jacs.3c12831

[50] Aboelella, N. W. et al. Snapshots of dioxygen activation by copper: the structure of a 1:1 Cu/O_2_ adduct and its use in syntheses of asymmetric Bis(*μ*-oxo) complexes. *J. Am. Chem. Soc.* **124**, 10660–10661 (2002). 12207513 10.1021/ja027164v

[51] Sarangi, R. et al. X-ray absorption edge spectroscopy and computational studies on LCuO_2_ species: superoxide-Cu^II^ versus peroxide-Cu^III^ Bonding. *J. Am. Chem. Soc.* **128**, 8286–8296 (2006). 16787093 10.1021/ja0615223 PMC2556900

[52] Seyeda, H. & Jansen, M. A novel access to ionic superoxides and the first accurate determination of the bond distance in . *J. Chem. Soc., Dalton Trans*. 875–876 10.1039/A800952J (1998).

[53] Tomson, N. C. et al. Re-evaluating the Cu K pre-edge XAS transition in complexes with covalent metal–ligand interactions. *Chem. Sci.* **6**, 2474–2487 (2015). 29308158 10.1039/c4sc03294b PMC5647745

[54] Chen, P., Root, D. E., Campochiaro, C., Fujisawa, K. & Solomon, E. I. Spectroscopic and electronic structure studies of the diamagnetic side-on Cu^II^-superoxo complex Cu(O_2_)[]: antiferromagnetic coupling versus covalent delocalization. *J. Am. Chem. Soc.* **125**, 466–474 (2003). 12517160 10.1021/ja020969i

[55] Sakata, K. & Amemiya, K. Time- and depth-resolved chemical state analysis of the surface-to-subsurface oxidation of Cu by x-ray absorption spectroscopy at near ambient pressure. *J. Phys. Chem. Lett.* **13**, 9573–9580 (2022). 36201653 10.1021/acs.jpclett.2c02641

[56] George, S. J., Lowery, M. D., Solomon, E. I. & Cramer, S. P. Copper L-edge spectral studies: a direct experimental probe of the ground-state covalency in the blue copper site in plastocyanin. *J. Am. Chem. Soc.* **115**, 2968–2969 (1993).

[57] Zhang, Y., Hua, W., Bennett, K. & Mukamel, S. Nonlinear spectroscopy of core and valence excitations using short x-ray pulses: simulation challenges. *Top. Curr. Chem.* **368**, 273–345 (2016). 25863816 10.1007/128_2014_618

[58] Feng, R., Yu, X. & Autschbach, J. Spin–orbit natural transition orbitals and spin-forbidden transitions. *J. Chem. Theory Comput.* **17**, 7531–7544 (2021). 34792327 10.1021/acs.jctc.1c00776

[59] Finley, J., Malmqvist, P. -Å, Roos, B. O. & Serrano-Andrés, L. The multi-state CASPT2 method. *Chem. Phys. Lett.* **288**, 299–306 (1998).

[60] Hirata, S. & Head-Gordon, M. Time-dependent density functional theory within the Tamm–Dancoff approximation. *Chem. Phys. Lett.* **314**, 291–299 (1999).

[61] Ruckman, M. W. et al. Interpreting the near edges of O_2_ and in alkali-metal superoxides. *Phys. Rev. Lett.* **67**, 2533–2536 (1991). 10044450 10.1103/PhysRevLett.67.2533

[62] Westre, T. E. et al. A multiplet analysis of Fe K-edge 1s → 3d pre-edge features of iron complexes. *J. Am. Chem. Soc.* **119**, 6297–6314 (1997).

[63] Hua, W. et al. Monitoring conical intersections in the ring opening of furan by attosecond stimulated X-ray Raman spectroscopy. *Struct. Dynam.* **3**, 023601 (2016). 10.1063/1.4933007 PMC4711522 26798832

[64] Aboelella, N. W. et al. Dioxygen activation at a single copper site: structure, bonding, and mechanism of formation of 1:1 Cu-O_2_ adducts. *J. Am. Chem. Soc.* **126**, 16896–16911 (2004). 15612729 10.1021/ja045678j

[65] Frisch, M. J. et al. *Gaussian 09, Revision D.01* (Gaussian, Inc.: Wallingford, CT, 2009).

[66] Couty, M. & Hall, M. B. Basis sets for transition metals: optimized outer *p* functions. *J. Comput. Chem.* **17**, 1359–1370 (1996). 25400155 10.1002/(SICI)1096-987X(199608)17:11<1359::AID-JCC9>3.0.CO;2-L

[67] Hay, P. J. & Wadt, W. R. Ab *initio* effective core potentials for molecular calculations. potentials for K to Au including the outermost core orbitals. *J. Chem. Phys.* **82**, 299–310 (1985).

[68] Fdez. Galván, I. et al. OpenMolcas: from source code to insight. *J. Chem. Theory Comput.* **15**, 5925–5964 (2019). 31509407 10.1021/acs.jctc.9b00532

[69] Aquilante, F. et al. Modern quantum chemistry with [open]molcas. *J. Chem. Phys.* **152**, 214117 (2020). 32505150 10.1063/5.0004835

[70] Malmqvist, P. A., Rendell, A. listair & Roos, B. O. The restricted active space self-consistent-field method, implemented with a split graph unitary group approach. *J. Phys. Chem.* **94**, 5477–5482 (1990).

[71] Delcey, M. G., Pedersen, T. B., Aquilante, F. & Lindh, R. Analytical gradients of the state-average complete active space self-consistent field method with density fitting. *J. Chem. Phys.* **143**, 44110 (2015). 10.1063/1.4927228 26233110

[72] Roos, B. O., Lindh, R., Malmqvist, P. -Å, Veryazov, V. & Widmark, P.-O. Main group atoms and dimers studied with a new relativistic ANO basis set. *J. Phys. Chem. A* **108**, 2851–2858 (2004).

[73] Delcey, M. G., Sørensen, L. K., Vacher, M., Couto, R. C. & Lundberg, M. Efficient calculations of a large number of highly excited states for multiconfigurational wavefunctions. *J. Comput. Chem.* **40**, 1789–1799 (2019). 30938847 10.1002/jcc.25832

[74] Boström, J. et al. Calibration of Cholesky auxiliary basis sets for multiconfigurational perturbation theory calculations of excitation energies. *J. Chem. Theory Comput.* **6**, 747–754 (2010). 26613305 10.1021/ct900612k

[75] Malmqvist, P. -Å & Roos, B. O. The CASSCF state interaction method. *Chem. Phys. Lett.* **155**, 189–194 (1989).

[76] Malmqvist, P. Å, Roos, B. O. & Schimmelpfennig, B. The restricted active space (RAS) state interaction approach with spin–orbit coupling. *Chem. Phys. Lett.* **357**, 230–240 (2002).

[77] Edmiston, C. & Ruedenberg, K. Localized atomic and molecular orbitals. *Rev. Mod. Phys.* **35**, 457–464 (1963).

[78] Boys, S. & Löwdin, P. Quantum theory of atoms, molecules, and the solid state (Academic Press, New York, 1966).

[79] Epifanovsky, E. et al. Software for the frontiers of quantum chemistry: an overview of developments in the Q-Chem 5 package. *J. Chem. Phys.* **155**, 84801 (2021). 10.1063/5.0055522 PMC9984241 34470363

[80] Mardirossian, N. & Head-Gordon, M. *ω*B97M-V: a combinatorially optimized, range-separated hybrid, meta-GGA density functional with VV10 nonlocal correlation. *J. Chem. Phys.* **144**, 214110 (2016). 27276948 10.1063/1.4952647

[81] Weigend, F., Furche, F. & Ahlrichs, R. Gaussian basis sets of quadruple zeta valence quality for atoms H–Kr. *J. Chem. Phys.* **119**, 12753–12762 (2003).

[82] Weigend, F. & Ahlrichs, R. Balanced basis sets of split valence, triple zeta valence and quadruple zeta valence quality for H to Rn: design and assessment of accuracy. *Phys. Chem. Chem. Phys.* **7**, 3297 (2005). 16240044 10.1039/b508541a

[83] Swope, W. C., Andersen, H. C., Berens, P. H. & Wilson, K. R. A computer simulation method for the calculation of equilibrium constants for the formation of physical clusters of molecules: Application to small water clusters. *J. Chem. Phys.* **76**, 637–649 (1982).

[84] Lu, T. & Chen, F. Multiwfn: a multifunctional wavefunction analyzer. *J. Comput. Chem.* **33**, 580–592 (2012). 22162017 10.1002/jcc.22885

[85] Humphrey, W., Dalke, A. & Schulten, K. VMD: visual molecular dynamics. *J. Mol. Graph.* **14**, 33–38 (1996). 8744570 10.1016/0263-7855(96)00018-5

