# Supplementary material for: Ultrafast metal-to-ligand electron transfer driven by bond shortening revealed through dual-edge computational X-ray spectroscopy
Source: Commun Chem. 2026 Apr 24;9:222. doi: 10.1038/s42004-026-02024-4 (PMC13315696; doi:10.1038/s42004-026-02024-4)
Supplement: Supplementary file 2 — Supplementary Information [file 42004_2026_2024_MOESM2_ESM.pdf]

Supplementary Information for: Ultrafast metal-to-ligand electron transfer driven by bond shortening revealed through dual-edge computational X-ray spectroscopy

Sheng-Yu Wang,[1,2] Jun-Rong Zhang,[2] Guoyan Ge,[2] and Weijie Hua[2]

[1]*School of Physics and Electronic Engineering, Research Institute of Optoelectronic Functional Materials, Jining University, Qufu, Shandong 273155, China*

[2]*MIIT Key Laboratory of Semiconductor Microstructure and Quantum Sensing, Department of Applied Physics, School of Physics, Nanjing University of Science and Technology, 210094 Nanjing, China*

(Dated: March 23, 2026)

Contents

Supplementary Notes S3

Note 1. Triplet Ground State Validation S3

Note 2. Supplementary Analyses S3
- A. Impact of Core Holes on DFT Energy Levels S3
- B. Active Orbitals and Their Occupation Number Changes S4
- C. Metal-to-Ligand Electron Transfer Analysis S4
- D. Vibrational Analysis for **2a** and **2b** S4
- E. Composition Analysis of Natural Transition Orbitals S6

Note 3. Supplementary Validations S6
- A. Structural Input Dependence S6
- B. Assessment of Multiconfigurational Parameters S7

Note 4. Note on Basis Set Selection S7

Supplementary Tables S9
- Table S1: DFT energies at optimized geometries . . . S9
- Table S2: Single-point energies across methods . . . S9
- Table S3: Active orbital occupations. . . . S9
- Table S4: NTO composition at **2a** . . . S10
- Table S5: NTO composition evolution . . . S10

Supplementary Figures S11
- Fig. S1: Gas-phase CuO_2^+ reaction pathway . . . S11
- Fig. S2: Ground state electronic structure . . . S12
- Fig. S3: Metal-to-ligand electron transfer evolution . . . S13
- Fig. S4: Core-hole effects on electronic structure . . . S14
- Fig. S5: Active space orbitals . . . S15
- Fig. S6: Complete NTO analysis for O K-edge TXAS . . . S15
- Fig. S7: DFT-computed vibrational modes of **2a** . . . S16
- Fig. S8: Structural dependence of Cu L-edge spectra . . . S16
- Fig. S9: Dynamic correlation effects on Cu L-edge XAS . . . S17
- Fig. S10: Basis set effects on Cu L-edge XAS . . . S18
- Fig. S11: Valence state averaging effects on Cu L-edge XAS . . . S19
- Fig. S12: Core-excited state averaging effects on Cu L-edge XAS . . . S20
- Fig. S13: Active space size effects on Cu L-edge XAS . . . S21

Supplementary References S22

Note on Organization: Supplementary Notes (1–4), Tables (S1–S5), and Figures (S1–S6) are numbered by first citation in the main text. Figures S7–S13 offer additional technical data and are referenced only within the Supplementary Notes.

Supplementary Notes

Note 1 Triplet Ground State Validation

The ground-state spin multiplicity of CuO_2^+ was determined by comparing the energies of the singlet, triplet, and quintet states. As summarized in Table S1, B3LYP calculations at the respective optimized geometries show that the triplet state is the most stable, with energies 0.44 eV lower than the singlet and 3.71 eV lower than the quintet.

To further validate this assignment, single-point energy calculations were performed using both B3LYP and RASPT2 methods at two key geometries: the triplet-optimized structure (**2b**) and the experimental structure (**2a**). The results, presented in Table S2, show consistent energy ordering across both methods. In all cases, the triplet state is energetically favored over the singlet, providing robust confirmation of the triplet ground state assignment.

Note 2 Supplementary Analyses

A Impact of Core Holes on DFT Energy Levels

To elucidate the electronic structure of CuO_2^+ and investigate core-hole effects, we computed energy level diagrams for three distinct states at structure **2a**: the ground electronic state, a full core-hole (FCH) state with an O 1s hole localized on O2 (see Fig. S4a), and an FCH state with a Cu 2p_y core hole. These calculations were performed using the Q-Chem[1] package with unrestricted DFT and the ωB97M-V functional[2]. A mixed basis set was employed, utilizing def2-QZVP[3] for O2 and def2-TZVP[4] for O1 and Cu to enhance the localization of degenerate O 1s orbitals. In all core-hole calculations, the hole was assigned to the β spin.

Figure S2 presents the ground-state energy level alongside MOs. The substantial HOMO–LUMO gaps (10.8 eV for α-spin, 10.5 eV for β-spin) signify a highly stable electronic configuration. The minimal spin polarization (0.3 eV difference between spin channels) indicates nearly degenerate orbital energy landscapes. This electronic structure is characteristic of a strongly bonded triplet ground state, wherein the two unpaired electrons occupy near-degenerate singly occupied molecular orbitals (SOMOs) 22α and 23α.

This description is consistent with multiconfigurational calculations (Section Note 2 B, Table S3), which show that both orbitals have occupations near unity. Furthermore, the three key frontier MOs–the Cu 3d_{xz}–O2 π^*_{ip} bond (23α, 22β), the O2 π^*_{oop} orbital (22α, 23β), and the Cu 3d_{xz}+O2 π^*_{ip} antibond (17α, 19β)–are located near the frontier in each spin channel and align with the active orbitals identified in the multiconfigurational analysis.

Figure S4 compares the ground-state energy levels with those of the O 1s and Cu 2p_y FCH states, revealing significant core-hole effects. The difference between the α and β HOMO-LUMO gaps differs by 0.3 eV in the ground state but increases to 3.0 eV for the O 1s core hole and 0.6 eV for the Cu 2p_y core hole. The more substantial effect of the O 1s

core hole is attributed to symmetry lowering from C_{2v} to C_s, while the symmetry of the Cu $2p_y$ state remains unchanged. This element-specific core-hole effect is further evidenced by irregularities in MO shapes (Fig. S4b), indicating a significant deviation in valence electron distribution symmetry due to the absence of a core electron.

In addition to affecting the HOMO-LUMO gap and MO symmetry, core holes can alter the order of MOs. For the β spin orbitals d_{xz}-π^*_{ip} and π^*_{oop}, the O $1s$ core hole causes an inversion in their energy levels, while the Cu $2p_y$ core hole preserves the ground-state order. This behavior may also be linked to the symmetry change. In both FCH states, the order of these orbitals aligns with trends observed in the XAS spectra at the O K-edge (Fig. 3) and Cu $L_{2,3}$-edge (Fig. 4).

B Active Orbitals and Their Occupation Number Changes

This section examines the changes in occupation numbers of active orbitals critical for understanding the electronic structure of CuO_2^+. The selection of active orbitals is guided by including Cu 3d and O_2 $2p$ orbitals, which accurately capture key interactions. For structure **2a**, we visualize eight active RAS2 orbitals at the converged state-averaged RASSCF wavefunction, as shown in Fig. S5a. These orbitals serve as the initial guess for subsequent calculations, including those for Cu $2p$ core-excited states (Fig. S5b). While most active orbitals effectively represent the valence state, noticeable changes in their occupation numbers are observed.

The RASSCF occupation numbers for the eight RAS2 orbitals (MOs 18-25) of $CuO2^+$ in its triplet ground state, evaluated at varying Cu-O distances, are detailed in Table S3. Among these, three critical orbitals are identified: MO 20 ($d_{xz} + \pi^*_{\mathrm{ip}}$), MO 22 ($\pi^*_{\mathrm{oop}}$), and MO 23 ($d_{xz} - \pi^*_{\mathrm{ip}}$). The partial occupation of approximately 1 for the antibonding orbitals (MOs 22 and 23) indicates significant static correlation. The persistent occupation of around 2.0 for the bonding orbital (MO 20) and approximately 1.0 for the antibonding orbital (MO 23) highlights a strongly correlated electron pair. This correlated pair is essential for the multi-configurational description of the Cu-O bond, thereby justifying the inclusion of both orbitals in the active space despite the integer occupation of MO 20.

C Metal-to-Ligand Electron Transfer Analysis

Figure S3 presents the natural population analysis (NPA) charges for the Cu and O2 units of CuO_2^+ computed at the RASPT2 level across a range of Cu–O bond lengths. The systematic evolution of these charges along the binding coordinate demonstrates a gradual MLET as the bond shortens. Quantitatively, the analysis indicates a net electron transfer of approximately $0.2e$ from structure **2b** ($q_{\mathrm{Cu}} = 1.0\ e$, $q_{\mathrm{O_2}} = -0.2\ e$) to **2a** ($q_{\mathrm{Cu}} = 1.2\ e$, $q_{\mathrm{O_2}} = -0.4\ e$).

D Vibrational Analysis for 2a and 2b

To provide a physically motivated reference for the proposed infrared-pump experiment, we computed the instantaneous vibrational frequencies at the fixed geometry of **2a** (the crystal-phase structure, $r_{\mathrm{Cu\text{-}O}} = 1.82$ Å, $r_{\mathrm{O\text{-}O}} = 1.39$ Å) and, for comparison, at the transition state geometry **2b** ($r_{\mathrm{Cu\text{-}O}} = 2.24$ Å, $r_{\mathrm{O\text{-}O}} = 1.23$ Å). Calculations were performed at the

B3LYP level of theory using the Gaussian 09 package,[5] with the 6-31G* basis set for oxygen and the modified LanL2DZ basis set (including optimized 4p functions[6]) and corresponding pseudopotential[7] for copper—the same level of theory used for geometry optimisations in this work. Frequencies were computed analytically at the fixed geometries without further relaxation.

Structure 2a. We emphasise that **2a** is *not* a stationary point on the gas-phase potential energy surface; therefore, a standard vibrational frequency analysis—which rigorously applies only at stationary points—would not yield physically meaningful frequencies in the strict harmonic oscillator sense. Nevertheless, the eigenvalues of the mass-weighted Hessian evaluated at any geometry provide a measure of the local curvature along each approximate normal mode direction. These directions, while not corresponding to true normal modes (which are strictly defined only at stationary points), still offer insight into the local shape of the potential energy surface and provide an estimate of the energy scale associated with small displacements from that geometry. In the context of designing an IR-pump experiment, this information can guide the selection of pump frequencies that may effectively couple to the relevant nuclear motions, under the assumption that the potential energy surface in the vicinity of the Franck-Condon region (here, the enzyme-constrained geometry **2a**) is approximately harmonic.

With this caveat in mind, the harmonic wavenumbers derived from the eigenvalues of the mass-weighted Hessian at **2a** are 513 cm^{-1}, 543 cm^{-1}, and 929 cm^{-1}. The corresponding approximate normal mode directions are visualised in Fig. S7 and can be characterised as follows. (1) The mode at 513 cm^{-1} corresponds primarily to the symmetric stretching of the two Cu–O bonds. (2) The mode at 543 cm^{-1} involves an anti-symmetric Cu–O stretching coupled with a bending motion of the O–Cu–O angle. (3) The highest frequency mode at 929 cm^{-1} is predominantly the O–O stretching vibration, which is red-shifted relative to the experimental values[8] of free O_2 ($\sim$1580 cm^{-1}) or O_2^+ ($\sim$1905 cm^{-1}) due to coordination to copper.

These values provide a useful estimate of the energy scales involved. In particular, the two lower-frequency modes (513 and 543 cm^{-1}) lie in the typical range for metal–ligand vibrations and could be targeted by mid-IR pump pulses to impulsively drive the system along the Cu–O symmetric stretching coordinate that we have identified as the key reaction coordinate for MLET. The 929 cm^{-1} mode, associated with O–O stretching, might also be relevant for probing the concomitant changes in the dioxygen moiety.

Structure 2b. For the transition state geometry **2b**, a standard vibrational frequency analysis is rigorously valid because **2b** is a stationary point on the gas-phase potential energy surface (a first-order saddle point). The calculation confirms its nature: one imaginary frequency is obtained at $214i$ cm^{-1}, corresponding to an antisymmetric stretching mode that tends to push the system toward the end-on configurations. The remaining two frequencies are real: 176 cm^{-1} (a symmetric mode that preserves the side-on geometry) and 1545 cm^{-1}. The frequency of 1545 cm^{-1} is very close to that of experimental free O_2 ($\sim$1580 cm^{-1}),[8] consistent with the Cu(I):O_2 characterisation of **2b** from XAS spectra and bond lengths.

The atomic displacement vectors of the imaginary mode (Fig. S1c) correspond precisely to the reaction coordinate revealed by the intrinsic reaction coordinate (IRC) calculation (Fig. S1a)—that is, the motion connecting the two equivalent end-on minima via the side-on transition state. This consistency verifies that **2b** is the genuine transition state for the gas-phase end-on $\rightleftharpoons$ end-on isomerization. The two real modes represent vibrations orthogonal to the reaction coordinate and are not expected to directly participate in the isomerization process.

We emphasise that the frequencies reported here are computed for single, non-stationary

(**2a**) or stationary (**2b**) geometries; in a full dynamical simulation, instantaneous frequencies would fluctuate as the molecule explores the potential energy surface. Nevertheless, they serve as a valuable starting point for designing time-resolved experiments aimed at triggering and observing the ground-state electron transfer process described in the main text.

E Composition Analysis of Natural Transition Orbitals

Table S4 presents the composition analysis for particle SO-NTOs in the Cu $L_{2,3}$-edge XAS spectrum at **2a**. The C-squared population analysis (SCPA) method, also known as the Ros-Schuit method[9], was employed with a threshold for the MO coefficient of $|C_i| > 0.5\%$. The main peaks at 931.8 eV (state 21) and 951.1 eV (state 146) arise from Cu $2p \rightarrow 3d$ transitions, exhibiting 21% Cu $3d$ character and 77% O $2p$ contributions. The weaker features at 933.6 eV (state 43), 936.6 eV (state 72), and 943.1 eV (state 114) are attributed to Cu $2p \rightarrow 4s$ transitions, which display dominant Cu $4s$ character (83.8–85.1%) and minimal O $2p$ contributions (4.8–6.1%).

The composition analysis was extended to include multiple structures between **2a** and **2b**. Table S5 illustrates the evolution of orbital contributions as Cu–O bond lengths increase. The Cu $3d$ character in the major transitions (peaks i and n) decreases monotonically from 21% to complete disappearance, while the O $2p$ contributions rise from 77% to 94%. In the satellite features (peaks k, l, m, and p), the Cu $4s$ character systematically increases from 84–85% to 88–92% with bond elongation. This progressive shift in orbital composition indicates a weakening of the metal-ligand interaction at longer bond distances, where the Cu $3d$–O $2p$ hybridization diminishes, allowing the more diffuse Cu $4s$ orbitals to play a relatively larger role in the excited states.

Note 3 Supplementary Validations

A Structural Input Dependence

Two experimental $LCuO_2$ structures with similar CuO_2 core geometries were reported: Reynolds *et al.*'s anilido-imine complex (1.82/1.83 Å Cu-O, 1.83/1.86 Å Cu-N) [10] and Aboelella *et al.*'s methyldiiminato analogue (uniform 1.82 Å Cu-O, 1.86 Å Cu-N) [11], both showing 1.39 Å O-O distances (Fig. S8). Notably, a subsequent XAS study by Sangari et al.[12] indexed both structures under the same compound designation.

While our prior study [13] used Reynolds' structure, we now employ Aboelella's symmetric variant to simplify the dependence on one varying structure parameter $r_{\text{Cu-O}}$. To validate, RASPT2 simulations of Cu $L_{2,3}$-edge XAS for both structures were performed, yielding largely similar spectra (Fig. S8). Minor intensity differences at 938 and 957 eV reveal spectral sensitivity to structural variations, while both simulated spectra reproduce the experimental data [12]. We therefore adopt Aboelella's $LCuO_2$ structure [11] (denoted **1**) as the starting geometry for CuO_2^+ (**2a**, with ligand L^- removed), as this choice does not affect our conclusions.

B Assessment of Multiconfigurational Parameters

To optimize our multiconfigurational approach for Cu $L_{2,3}$-edge spectra, we systematically evaluated: (1) dynamic correlation (RASPT2 vs. RASSCF), (2) basis sets, (3) state averaging (n_v=5–100, n_c=0–100), and (4) RAS2 active space size (n_A=2–8). Each test was conducted while keeping other parameters constant. Figures S9–S13 illustrate the following findings:

Minimal Sensitivity to Dynamic Correlation. RASPT2 and RASSCF yield nearly identical spectra (Fig. S9), differing only slightly in spectral profiles while maintaining nearly equal energy positions.

Basis Set Dependence. Although VDZP was ultimately selected, larger basis sets enhance absolute energy precision without significantly altering spectral shapes (Fig. S10).

Robustness to State Averaging. Spectra show negligible variation across the tested ranges of n_v (5–100, Fig. S11) and n_c (0–100, Fig. S12). Optimal values (n_v=5, n_c=40) ensure convergence.

Active Space Sensitivity. While n_A=6 and 8 produce qualitatively similar spectra (Fig. S13), the larger active space better resolves minor peaks. We have adopted n_A=6 for a balance of accuracy and cost.

These systematic validations confirm the excellent method robustness for simulating Cu L-edge spectra of CuO_2^+, despite the absence of experimental results thus far.

Note 4 Note on Basis Set Selection

Different basis sets were employed in this study. They were selected to meet the specific needs of each methodological stage. Validation focused primarily on the most sensitive part of the calculation, the Cu L-edge XAS simulated with RASPT2 (Note 3B), while the overall protocol is further validated by agreement with experimental benchmarks. Therefore, the varied basis set choices do not compromise the accuracy of our calculations or the robustness of our conclusions. The specific choices are summarized below:

Geometry Optimizations (Gaussian 09): 6-31G* and modified LanL2DZ. The Pople-style 6-31G* basis set was used for light atoms, while the modified LanL2DZ basis set and associated LanL2DZ pseudo potential were applied to copper. This hybrid combination is a well-established and computationally efficient standard in transition-metal chemistry, providing reliable equilibrium geometries at tractable cost.

RASPT2 Spectrum Simulations (OpenMolcas): ANO-RCC-VDZP. For the high-accuracy calculation of core-level (Cu L-edge and O K-edge) spectra, the ANO-RCC-VDZP basis set was employed. This set is specifically designed within the ANO framework for multiconfigurational methods and incorporates scalar relativistic corrections via the Douglas–Kroll–Hess Hamiltonian. Our systematic validation (Fig. S10) confirmed its suitability for the Cu L-edge, making it the optimal choice for correlated spectroscopy within the OpenMolcas ecosystem.

TDDFT Spectrum Simulations (Q-Chem): def2-TZVP and def2-QZVP. For comparative time-dependent density functional theory (TDDFT) calculations, the def2-TZVP and def2-QZVP basis sets from the Ahlrichs–Weigend series were used. The def2 suite is a general-purpose, highly optimized standard for molecular DFT and TDDFT, offering a systematic path to convergence and excellent performance in modern quantum chemistry packages such as Q-Chem.

These choices reflect established best practices in their respective domains and together provide a consistent, validated framework for the spectroscopic simulations presented in this work.

Supplementary Tables

TABLE S1: **DFT energies at optimized geometries**. Total energies (in a.u.) of CuO_2^+ at the optimized geometries for different spin states, computed using B3LYP. Relative energies (in eV, in parentheses) are given with respect to the triplet ground state (structure **2b**).

Singlet	Triplet	Quintet
-346.14637 (0.44)	-346.16264 (0.00)	-346.02638 (3.71)

TABLE S2: **Single-point energies across methods**. Total energies (in a.u.) of CuO_2^+ for the lowest singlet and triplet states at structures **2a** and **2b**, computed using RASPT2 and B3LYP. Relative energies (in eV) are shown in parentheses, referenced to the respective triplet state energy at each geometry.

CuO_2^+	Method	Singlet	Triplet
**2a**	RASPT2	-1803.37866 (0.66)	-1803.40307 (0.00)
	B3LYP	-346.10882 (0.44)	-346.12497 (0.00)
**2b**	RASPT2	-1803.39052 (0.92)	-1803.42433 (0.00)
	B3LYP	-346.10716 (1.51)	-346.16264 (0.00)

TABLE S3: **Active orbital occupations**. RASSCF occupation numbers for the eight RAS2 orbitals (MOs 18-25) of CuO_2^+ in its triplet ground state at varying Cu-O distances ($r_{\text{Cu-O}}$, Å). The RAS2 orbitals at structure **2a** ($r_{\text{Cu-O}} = 1.82$ Å) are visualized in Fig. S5a. Critical orbitals are highlighted in bold: MOs 20 ($d_{xz} + \pi^*_{\text{ip}}$), 22 ($\pi^*_{\text{oop}}$), and 23 ($d_{xz} - \pi^*_{\text{ip}}$).

$r_{\text{Cu-O}}$	18	19	**20**	21	**22**	**23**	24	25
1.82	1.95	1.92	1.99	1.95	1.05	1.04	0.02	0.08
1.89	1.95	1.93	1.99	1.95	1.05	1.04	0.02	0.07
1.96	1.95	1.93	2.00	1.95	1.05	1.04	0.02	0.07
2.03	1.95	1.94	2.00	1.95	1.05	1.04	0.02	0.06
2.10	1.95	1.94	2.00	1.95	1.04	1.04	0.02	0.06
2.17	1.96	1.95	2.00	1.95	1.04	1.04	0.02	0.05
2.24	1.96	1.96	2.00	1.95	1.04	1.03	0.01	0.05

TABLE S4: **NTO composition at 2a**. Composition analysis of particle SO-NTOs for structure **2a**, with state indexes corresponding to labeled peaks in the Cu $L_{2,3}$-edge XAS spectra (Fig. 2e). Peak energies (eV) and assignments are included.

SO-state	Energy	Composition (%)			Assignment
		Cu 3d	Cu 4s	O 2p	
21	931.8	21.4	–	77.4	Cu $3d_{xz} - \pi^*_{\mathrm{ip}}$
31	933.1	0.6	–	99.2	π^*_{oop}
43	933.6	0.6	83.8	6.1	Cu 4s
72	936.6	–	85.1	4.8	Cu 4s
83	937.1	5.4	7.1	83.2	Cu $3d - \pi_{\mathrm{ip}}$
114	943.1	–	85.1	4.8	Cu 4s
146	951.1	21.4	–	77.4	Cu $3d_{xz} - \pi^*_{\mathrm{ip}}$
151	952.6	0.6	–	99.2	π^*_{oop}
154	952.9	0.6	83.8	6.1	Cu 4s

TABLE S5: **NTO composition evolution**. Composition analysis (%) of particle SO-NTOs for selected CuO_2^+ structures between **2a** ($r_{\text{Cu-O}} = 1.82$ Å) and **2b** ($r_{\text{Cu-O}} = 2.24$ Å). Peaks correspond to features in Cu $L_{2,3}$-edge XAS spectra (Fig. 4a).

$r_{\text{Cu-O}}$	i			j			k			l		
	d	s	p	d	s	p	d	s	p	d	s	p
1.82	21↓	–	77↑	0.6↓	–	99↑	0.6↓	84↑	6↓	–	85↑	5↓
1.96	15	–	84	–	–	100	0.5	86	6	–	88	5
2.10	5	–	93	–	–	100	–	88	6	–	90	4
2.24	–	–	95	–	–	–	–	92	4	–	92	4

$r_{\text{Cu-O}}$	m			n			o			p		
	d	s	p	d	s	p	d	s	p	d	s	p
1.82	–	85↑	5↓	21↓	–	77↑	0.6↓	–	99↑	0.6↓	84↑	6↓
1.96	–	88	5	15	–	84	–	–	100	0.5	86	6
2.10	–	90	4	6.5	–	93	–	–	–	–	88	6
2.24	–	92	4	4.9	–	94	–	–	–	–	91	5

[a] Entries marked "–" indicate components <4.0%.
[b] Symbols ↑/↓ track component increases/decreases with Cu–O bond length.
[c] All values are percentages. d = Cu 3d, s = Cu 4s, p = O 2p orbitals.

Supplementary Figures

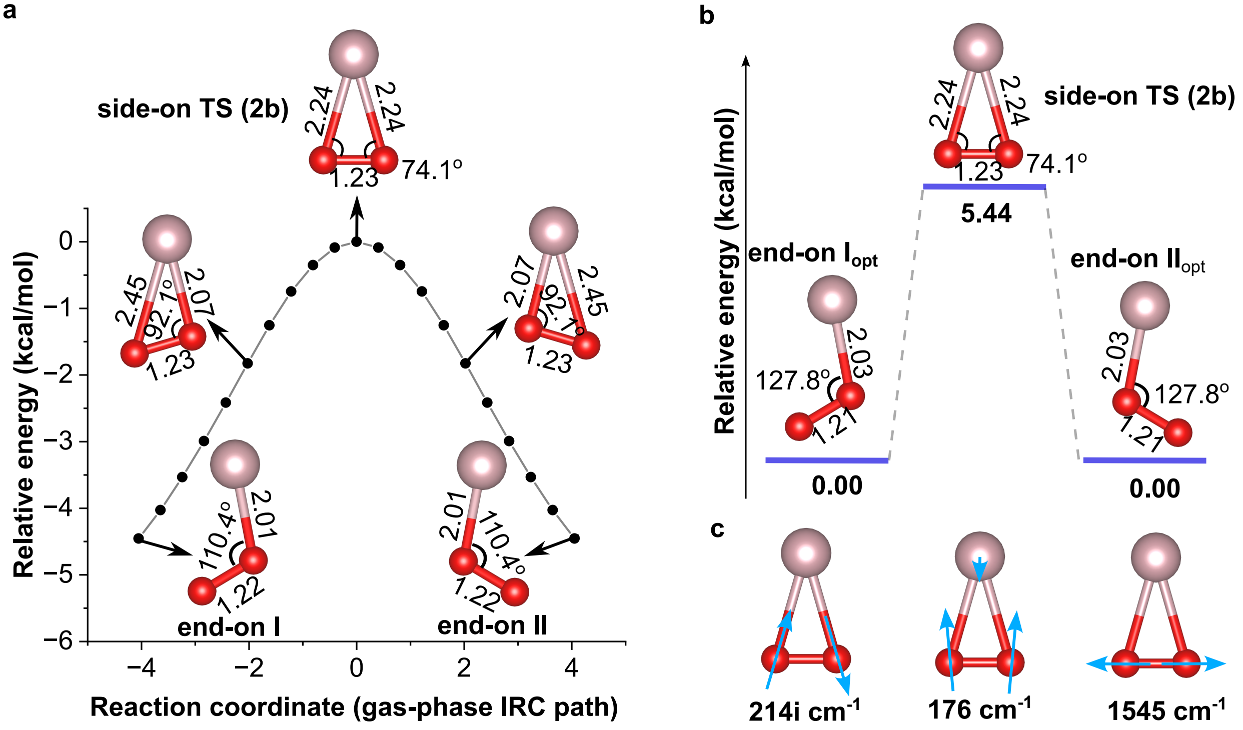

FIG. S1: **Characterization of the gas-phase transition state (TS) of CuO_2^+.** (a) Gas-phase IRC path for CuO_2^+. The side-on TS **(2b)** connects two unoptimized end-on geometries, labeled "end-on I" and "end-on II". (b) Corresponding energy level diagram. "end-on I_{opt}" and "end-on II_{opt}" denote the fully optimized geometries of the two end-on minima. Note that this gas-phase path is orthogonal to the enzyme-constrained side-on compression coordinate (**2b** → **2a**) studied in the main text. In (a) and (b), selected bond lengths (in Å) and angles (in °) are labeled. (c) DFT-computed vibrational modes of the side-on TS (**2b**). Arrows indicate vibrational modes. The imaginary frequency mode at $214i$ cm^{-1} is consistent with the IRC path shown in (a).

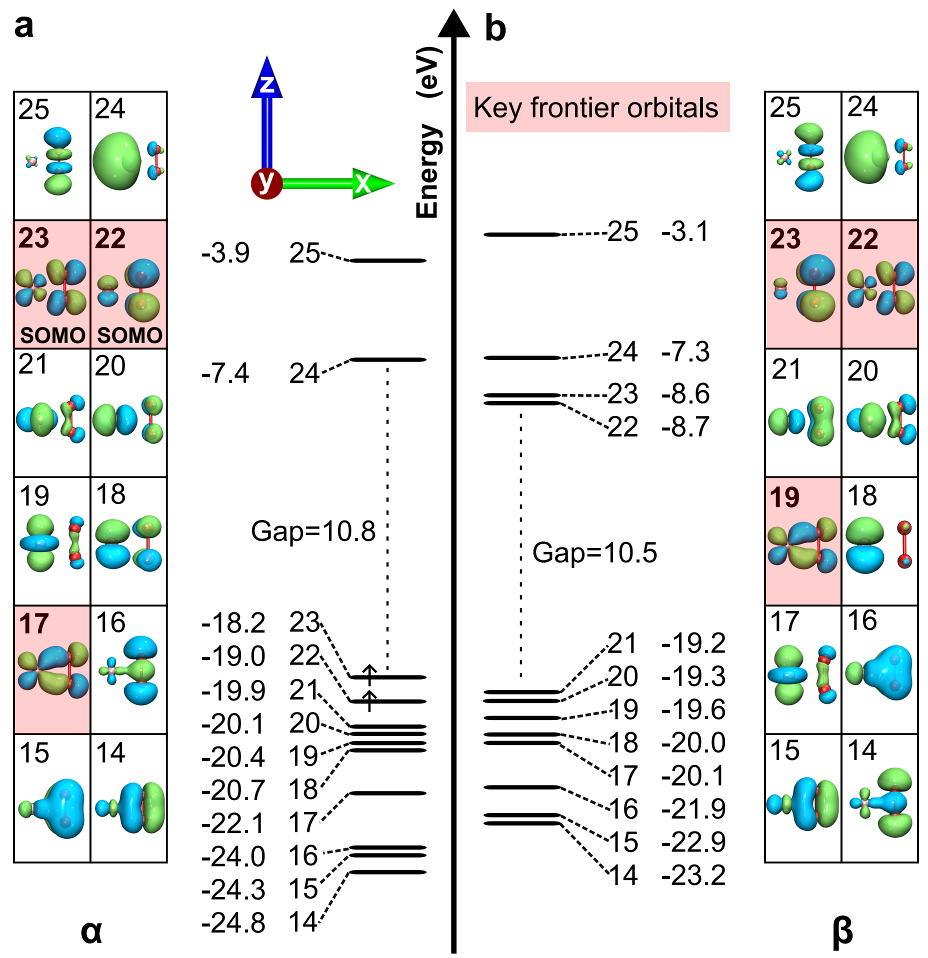

FIG. S2: **Ground state electronic structure.** Unrestricted DFT (ωB97M-V) computed molecular orbital energy level diagram for the triplet ground state of structure **2a**. Arrows indicate SOMOs. MOs are depicted adjacent to their corresponding energy levels with the three key frontier orbitals highlighted (bold, red) for each spin channel: Cu $3d_{xz}$–O_2 π^*_{ip} (23α, 22β), π^*_{oop} (22α, 23β), and Cu $3d_{xz}$+O_2 $\pi^*_{\mathrm{ip}}O_2$ (17α, 19β). The HOMO–LUMO gap for each spin is indicated (in eV).

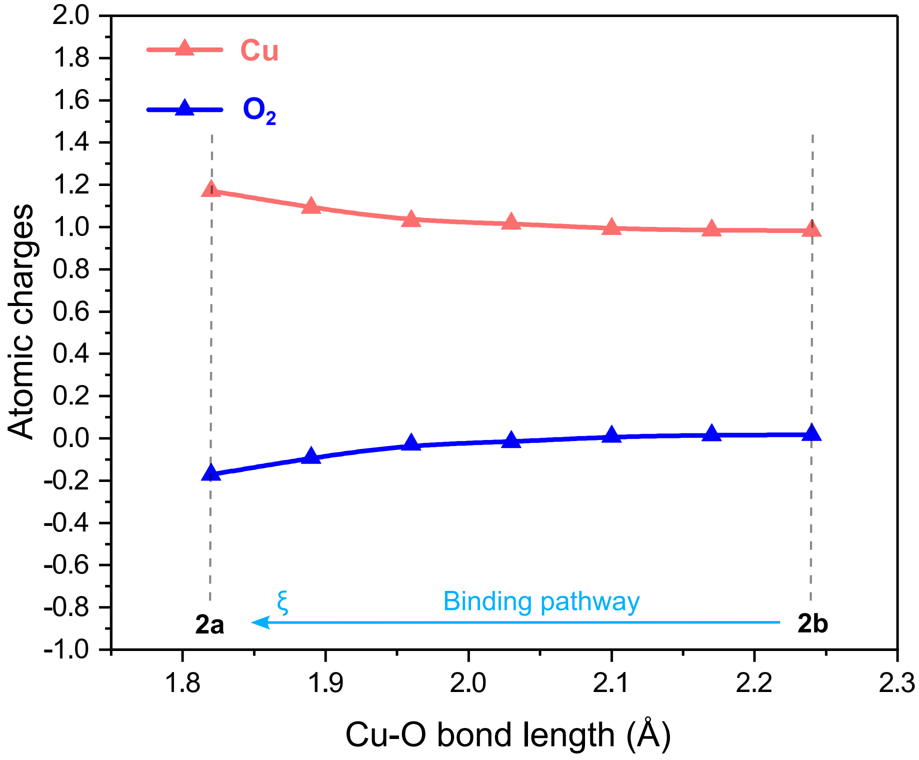

FIG. S3: **Metal-to-ligand electron transfer evolution.** Natural population analysis charges for Cu and O_2 fragments in CuO_2^+ as a function of Cu–O distance, computed at RASPT2 level. The systematic charge evolution from structure **2b** (2.24 Å) to **2a** (1.82 Å) indicates a net metal-to-ligand electron transfer of $0.2e$, consistent with the Cu(I) to Cu(II) transition assigned from XAS calculations.

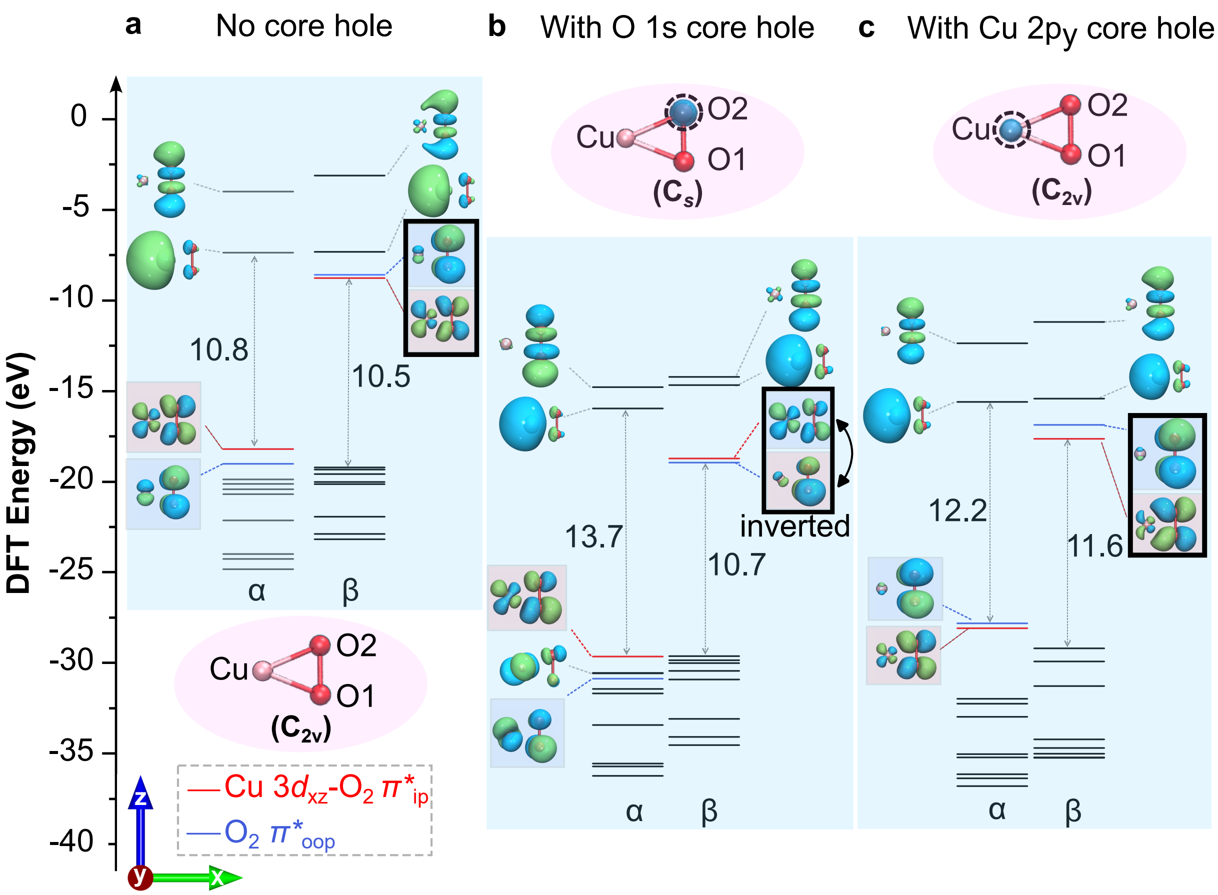

FIG. S4: **Core-hole effects on electronic structure.** Unrestricted DFT (ωB97M-V) computed molecular orbital energy level diagrams for structure **2a**: (a) ground electronic state (triplet), (b) FCH state with an O 1s core hole (quartet), and (c) FCH state with a Cu $2p_y$ core hole (quartet). Two critical orbitals are highlighted: Cu $3d_{xz}$–O_2 π^*_{ip} (red) and O_2 π^*_{oop} (blue). In each panel, molecular symmetry is labeled. An energy level inversion occurs in the β-spin channel (hosting the core hole) in (b). Several additional frontier MOs are also depicted.

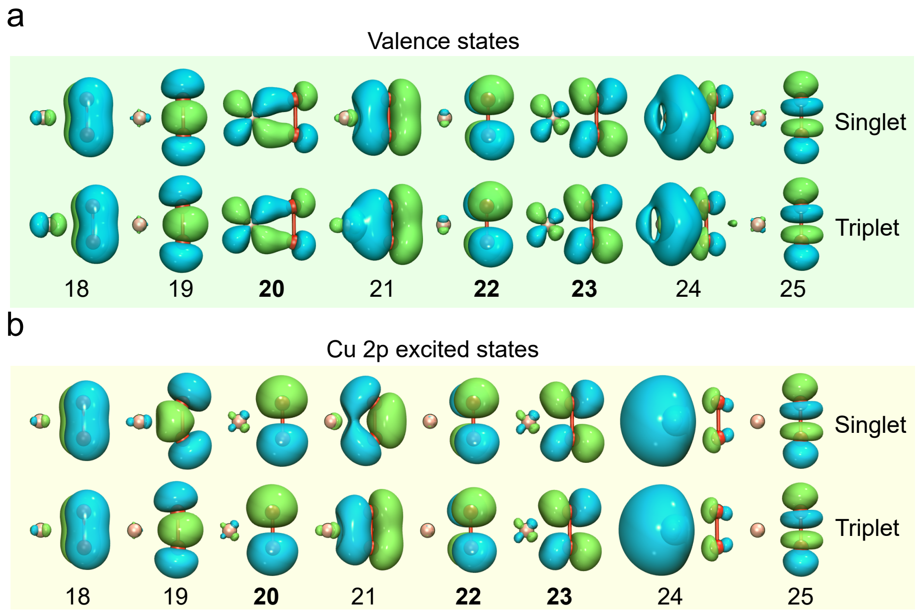

FIG. S5: **Active space orbitals.** Convergent RAS2 active orbitals of CuO_2^+ structure **2a** from state-averaged RASSCF calculations for (a) 5 valence and (b) 40 Cu 2p core-excited states. Critical orbitals are highlighted: MOs 20 ($d_{xz} + \pi^*_{\mathrm{ip}}$), 22 ($\pi^*_{\mathrm{oop}}$), and 23 ($d_{xz} - \pi^*_{\mathrm{ip}}$).

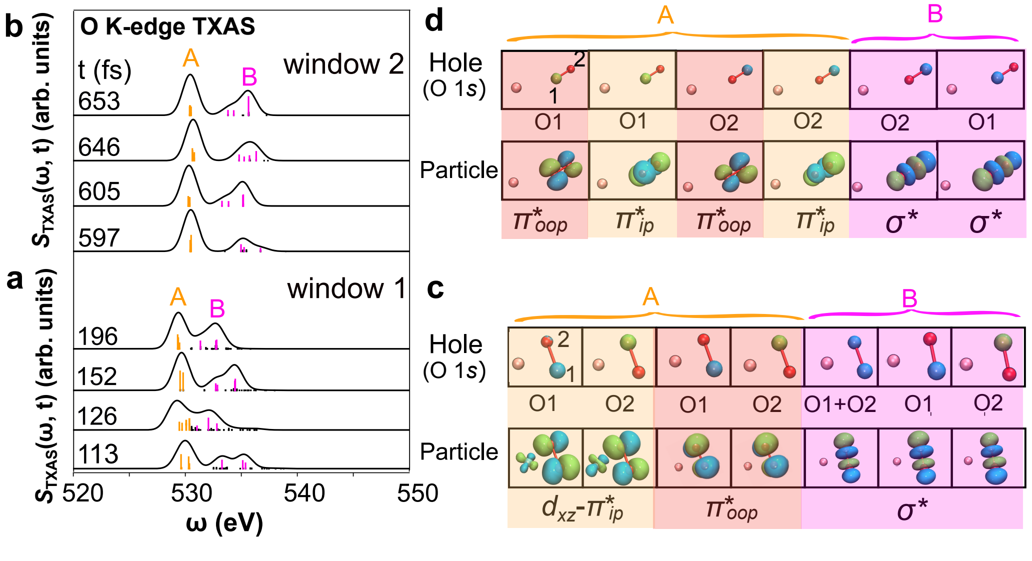

FIG. S6: **Complete NTO analysis for O K-edge TXAS.** (a, b) Simulated spectra for four representative BOMD snapshots in (a) window 1 (side-on, 97–242 fs) and (b) window 2 (end-on, 581–774 fs). (c, d) Corresponding complete set of NTOs (both particle and hole orbitals) for the dominant transitions. Each orbital category is shown in a distinct shaded color (consistent color scheme used throughout the manuscript). This figure provides the complementary hole-orbital analysis not included in Fig. 6f (which shows only particle NTOs). The hole orbital is either localized on a single oxygen (O1 or O2) or delocalized across both atoms within our computational protocol.

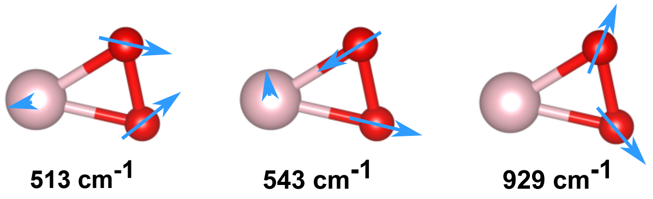

FIG. S7: **DFT-computed vibrational modes of 2a**. Arrows indicate vibrational modes.

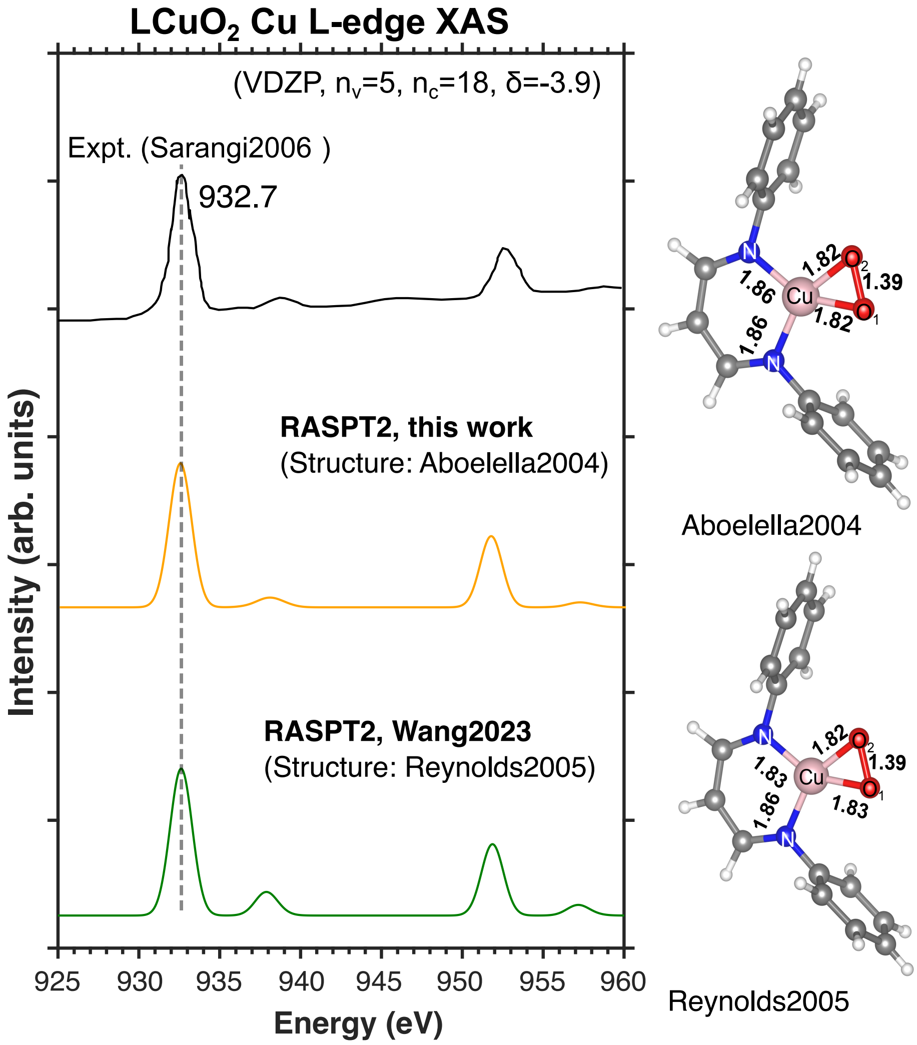

FIG. S8: **Structural dependence of Cu L-edge spectra.** Cu $L_{2,3}$-edge XAS spectra of $LCuO_2$ simulated for geometries from Reynolds et al.[10] and Aboelella et al.[11] crystal structures. RASPT2 calculations used consistent models and are compared with experimental data.[12] The spectrum for the Reynolds structure (green) is adapted from our previous work[13], while the Aboelella structure spectrum (orange) was computed in this work. Both structures yield similar spectra, validating the use of Aboelella's symmetric structure in the current study.

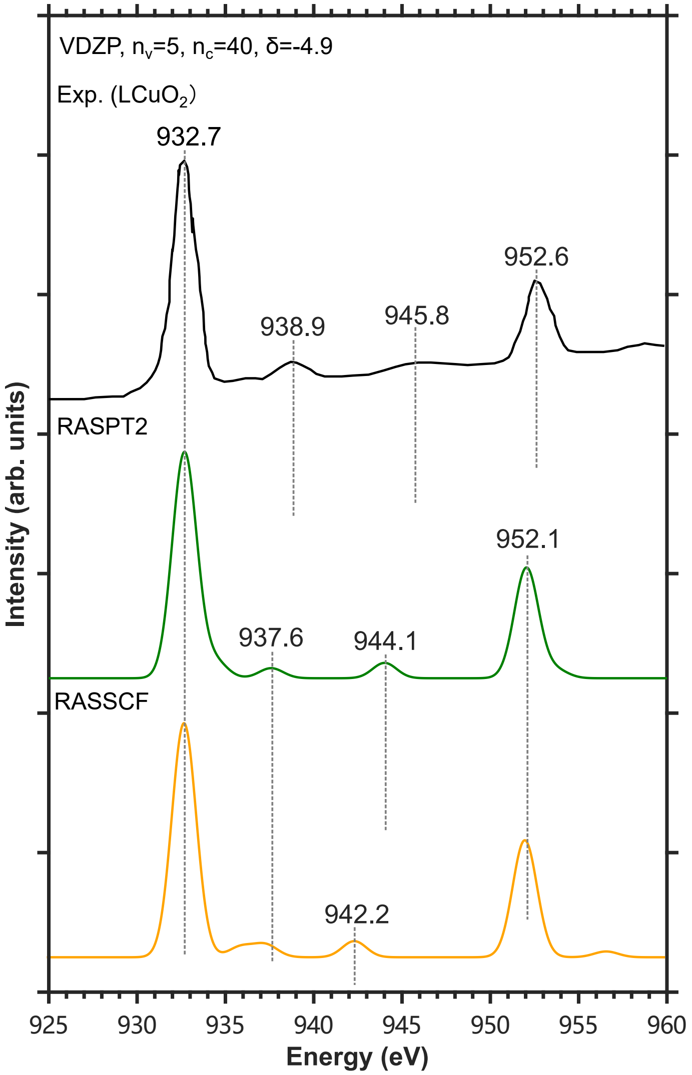

FIG. S9: **Dynamic correlation effects on Cu L-edge XAS.** Simulated Cu $L_{2,3}$-edge XAS spectra of CuO_2^+ structure **2a** computed using RASSCF and RASPT2 methods. Theoretical spectra are shifted by $\delta = -4.9$ eV to align with experimental data. Both methods yield nearly identical spectra, indicating minimal sensitivity to dynamic correlation.

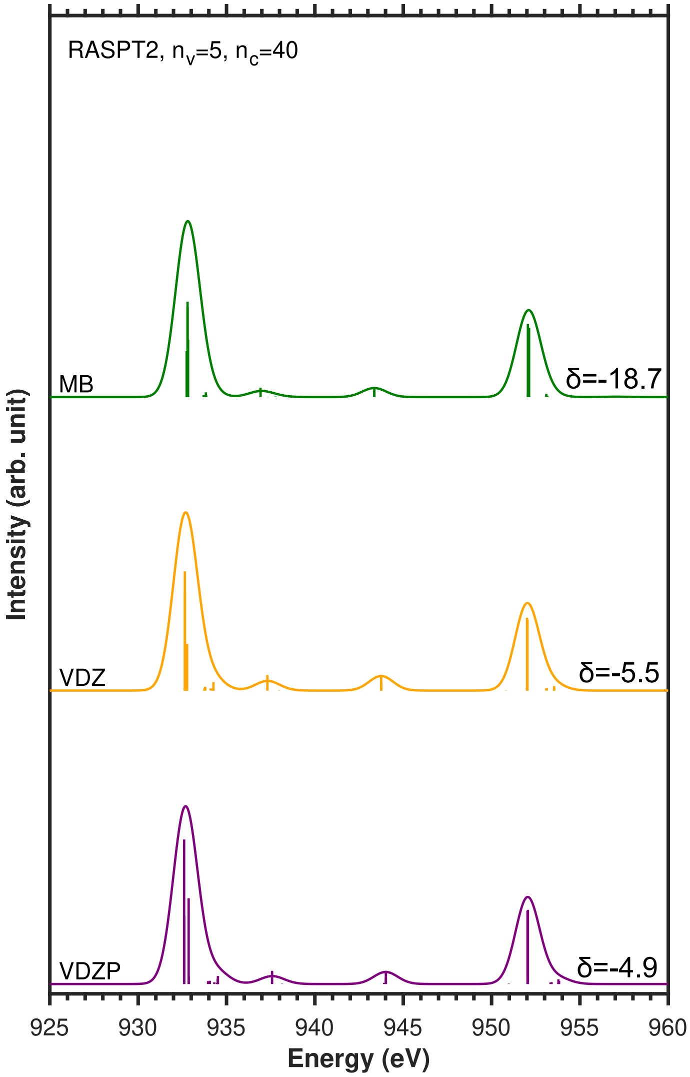

FIG. S10: **Basis set effects on Cu L-edge XAS.** Cu $L_{2,3}$-edge XAS spectra of CuO_2^+ structure **2a** computed with different ANO-RCC basis sets. Spectra show consistent shapes across basis sets, with VDZP selected for optimal balance of accuracy and computational cost.

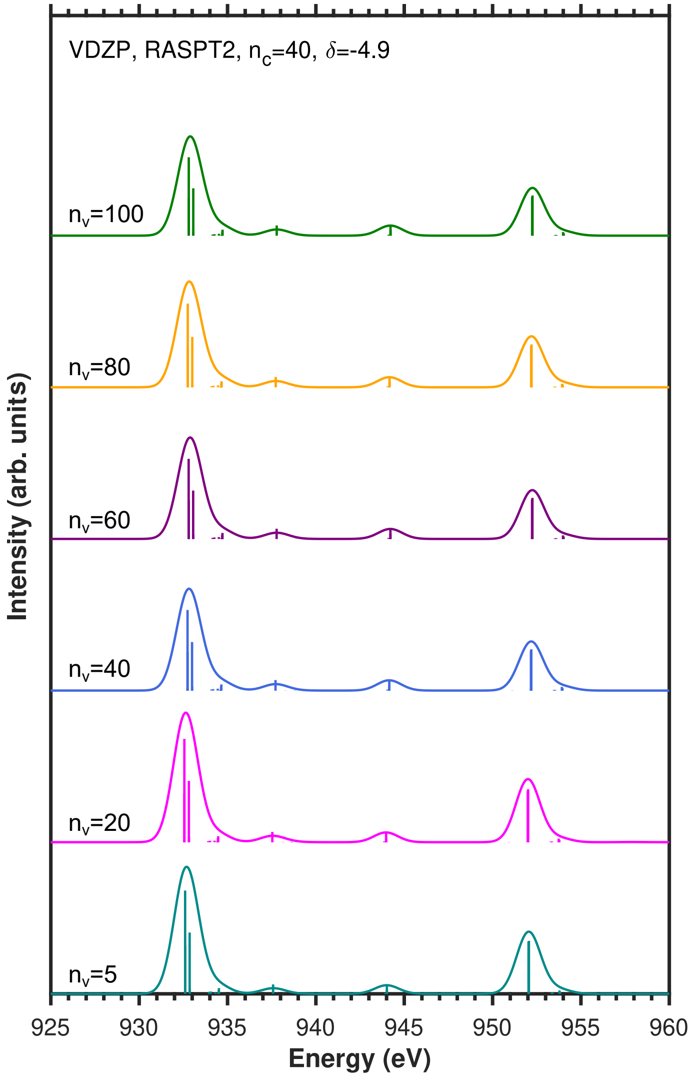

FIG. S11: **Valence state averaging effects on Cu L-edge XAS.** Cu $L_{2,3}$-edge XAS spectra of CuO_2^+ structure **2a** computed with different numbers of valence states (n_v). Spectra show negligible variation, confirming convergence with $n_v = 5$.

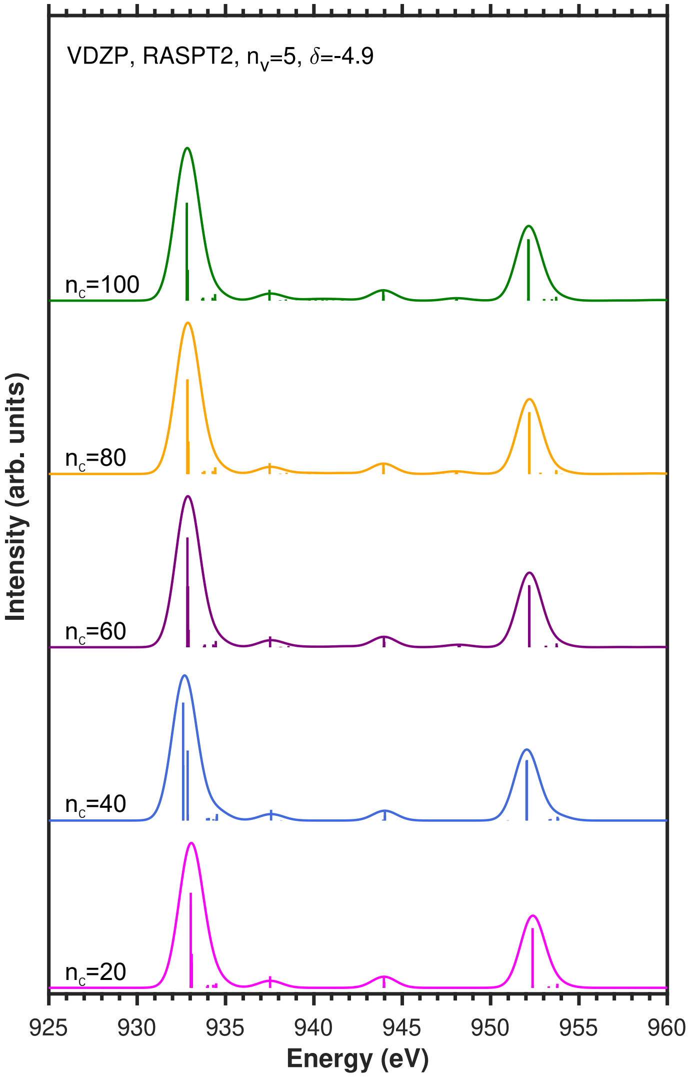

FIG. S12: **Core-excited state averaging effects on Cu L-edge XAS.** Cu $L_{2,3}$-edge XAS spectra of CuO_2^+ structure **2a** computed with different numbers of core-excited states (n_c). Spectral convergence is achieved with $n_c = 40$.

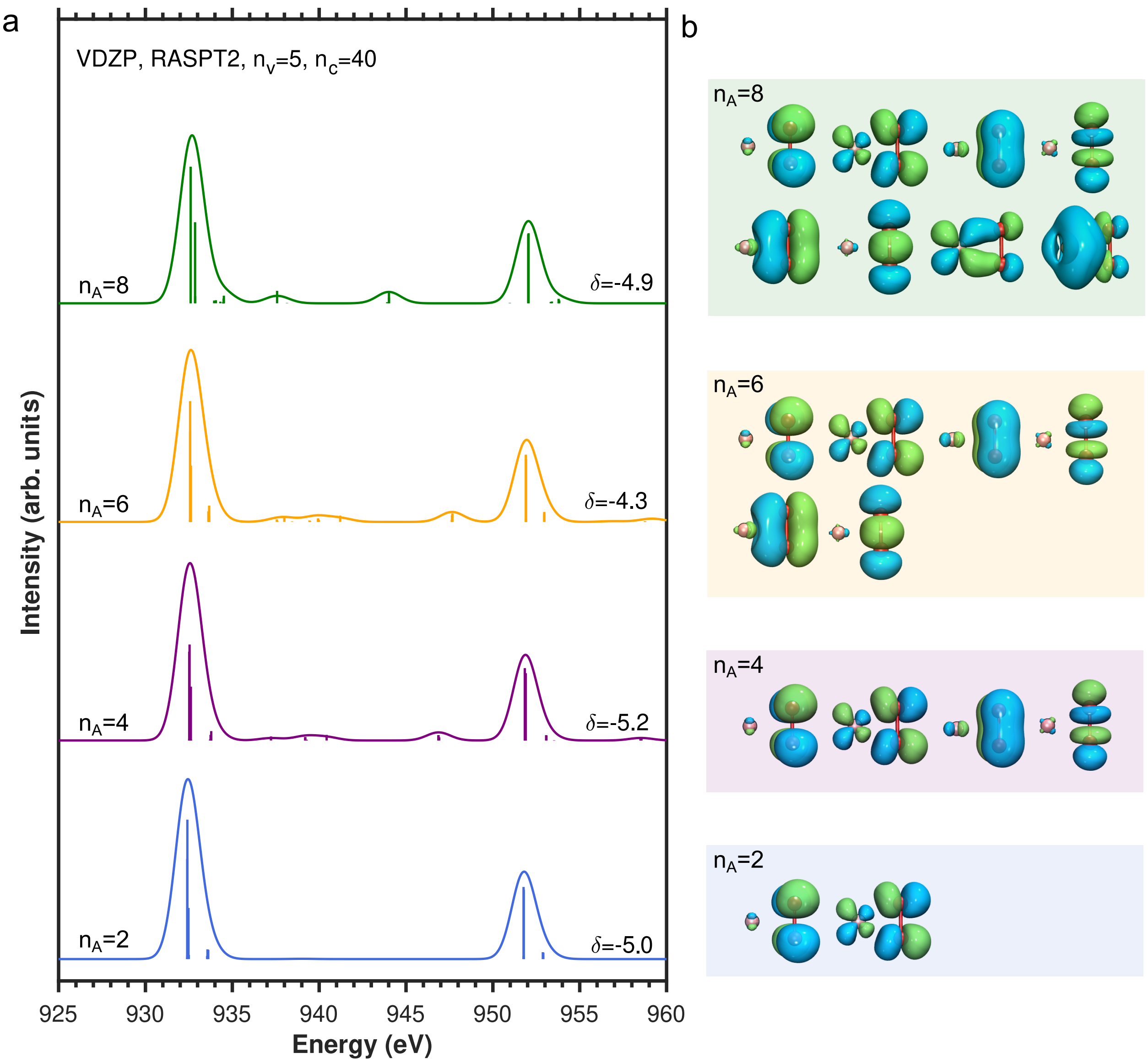

FIG. S13: **Active space size effects on Cu L-edge XAS.** (a) Cu $L_{2,3}$-edge XAS spectra of $CuO_2{}^+$ structure **2a** computed with different numbers of active orbitals (n_A). (b) Corresponding RAS2 active orbitals. Larger active spaces better resolve minor spectral features while maintaining qualitative agreement.

[1] Epifanovsky, E. *et al.* Software for the Frontiers of Quantum Chemistry: An Overview of Developments in the Q-chem 5 Package. *J. Chem. Phys.* **155**, 84801 (2021).

[2] Mardirossian, N. & Head-Gordon, M. ωB97M-V: A Combinatorially Optimized, Range-Separated Hybrid, Meta-GGA Density Functional with VV10 Nonlocal Correlation. *J. Chem. Phys.* **144**, 214110 (2016).

[3] Weigend, F., Furche, F. & Ahlrichs, R. Gaussian Basis Sets of Quadruple Zeta Valence Quality for Atoms H–Kr. *J. Chem. Phys.* **119**, 12753–12762 (2003).

[4] Weigend, F. & Ahlrichs, R. Balanced Basis Sets of Split Valence, Triple Zeta Valence and Quadruple Zeta Valence Quality for H to Rn: Design and Assessment of Accuracy. *Phys. Chem. Chem. Phys.* **7**, 3297 (2005).

[5] Frisch, M. J. *et al.* Gaussian 09, Revision D.01. Gaussian, Inc.: Wallingford, CT, 2009.

[6] Couty, M. & Hall, M. B. Basis Sets for Transition Metals: Optimized Outerp Functions. *J. Comput. Chem.* **17**, 1359–1370 (1996).

[7] Hay, P. J. & Wadt, W. R. Ab initio effective core potentials for molecular calculations. potentials for k to au including the outermost core orbitals. *J. Chem. Phys.* **82**, 299–310 (1985).

[8] Huber, K. P. & Herzberg, G. H. *Constants of Diatomic Molecules* (Van Nostrand-Reinhold, New York, 1979).

[9] Ros, P. & Schuit, G. C. A. Molecular Orbital Calculations on Copper Chloride Complexes. *Theor. Chim. Acta* **4**, 1–12 (1966).

[10] Reynolds, A. M., Gherman, B. F., Cramer, C. J. & Tolman, W. B. Characterization of a 1:1 Cu-O_2 Adduct Supported by an Anilido Imine Ligand. *Inorg. Chem.* **44**, 6989–6997 (2005).

[11] Aboelella, N. W. *et al.* Dioxygen Activation at a Single Copper Site: Structure, Bonding, and Mechanism of Formation of 1:1 Cu-O_2 Adducts. *J. Am. Chem. Soc.* **126**, 16896–16911 (2004).

[12] Sarangi, R. *et al.* X-Ray Absorption Edge Spectroscopy and Computational Studies on $LCuO_2$ Species: superoxide-Cu^{II} versus peroxide-Cu^{III} Bonding. *J. Am. Chem. Soc.* **128**, 8286–8296 (2006).

[13] Wang, S.-Y., Zhang, J.-R., Guo, M. & Hua, W. Interpreting the Cu–O_2 Antibonding Nature in Two Cu–O_2 Complexes from Cu L-edge X-Ray Absorption Spectra. *Inorg. Chem.* **62**, 17115–17125 (2023).
