# Supplementary material for: Ultrafast metal-to-ligand electron transfer driven by bond shortening revealed through dual-edge computational X-ray spectroscopy
Source: Commun Chem. 2026 Apr 24;9:222. doi: 10.1038/s42004-026-02024-4 (PMC13315696; doi:10.1038/s42004-026-02024-4)
Supplement: Supplementary file 3 — Description of Additional Supplementary Files [file 42004_2026_2024_MOESM3_ESM.docx]

**File:** Supplementary Movie 1

**Description:** Animation of structural dynamics during 0–400 fs.

The other supplementary data files (originally Supplementary Data 1, 2, 3, 4, 5A, 5B, 6A, 6B, 6D, 6E) are available in Zenodo (see Data Availability Statement).
