# Supplementary material for: Ultrafast metal-to-ligand electron transfer driven by bond shortening revealed through dual-edge computational X-ray spectroscopy
Source: Commun Chem. 2026 Apr 24;9:222. doi: 10.1038/s42004-026-02024-4 (PMC13315696; doi:10.1038/s42004-026-02024-4)
Supplement: Supplementary file 1 — Transparent Peer Review file [file 42004_2026_2024_MOESM1_ESM.pdf]

nature portfolio

Peer Review File

Ultrafast metal-to-ligand electron transfer driven by bond shortening revealed through dual-edge computational X-ray spectroscopy

Corresponding Author: Professor Weijie Hua

This file contains all reviewer reports in order by version, followed by all author rebuttals in order by version.

Version 0:

Reviewer comments:

Reviewer #1

(Remarks to the Author)
Ultrafast metal-to-ligand charge transfer driven by bond shortening revealed with dual-edge computational X-ray spectroscopy,
Sheng-Yu Wang, Jun-Rong Zhang, Guoyan Ge, Weijie Hua

In this paper, the authors introduce a dual-edge computational X-ray spectroscopy approach to track how electrons move during ultrafast chemical reactions in transition metal complexes. The work focuses on the $CuO2+$ system to understand how changes in geometry drive metal-to-ligand charge transfer. Using computed Cu L2,3 and O K-edge X-ray absorption spectra along the Cu-O bond using
multiconfigurational RASPT2 and TDDFT methods, the study shows that symmetric Cu–O bond shortening triggers a fast conversion from Cu(I):O2 to Cu(II):O2-. The dual-edge analysis uses the simpler ligand-centered O K-edge to interpret the more complex metal-centered L-edge, providing a clear picture of how electrons flow during bond rearrangement. The authors suggest that this is a general way to interpret ultrafast X-ray data and can be used to follow redox dynamics in catalysts, metalloenzymes, and energy conversion systems to name a few.

The use of dual-edge XAS analysis is not new and has been widely applied in the X-ray literature to understand electronic structure changes driven by bond rearrangements. I encourage the authors to include additional references for the benefit of readers who may be less familiar with this area. However, this paper is fairly well written, and I will support its publication once the authors have addressed the following review comments.

1. Overall, the distinction between the binding coordinate and the reaction coordinate is not clear here. Additionally, the work refers to ultrafast processes several times while presenting only spectra calculated for static structures, which is misleading.

2. The authors should refine their discussion and specify that they are referring to the field of soft X-ray spectroscopy here (50-1200 eV).

2. Line 22: The authors should refer either to transient X-ray spectroscopy, which provides insights into dynamics, or to modern X-ray spectroscopy, which provides information on structural details and oxidation states. Modern spectroscopies are not necessarily time-resolved.

3. Line 34-35: The authors should consider rewording their sentence to state that an accurate framework should accurately capture the dynamics as well as predicting the spectral signatures. However, no dynamics were actually simulated in this paper.

4. Line 80: It seems that the Cu L-edge would be more sensitive to the oxidation state than the O K-edge. What additional insight is gained from analyzing the O edge? Perhaps this could be connected to potential ligand interactions. It is also understandable that the O K-edge is computationally simpler to treat. The discussion could be refined further to clarify these points for a broader audience.

5. Line 86: It is clear that the authors are not modeling ultrafast dynamics but are instead interpolating between a few static structures. As mentioned earlier, this is misleading and should be clarified. The authors should also explain how the intermediate structures were generated. How is the reaction pathway defined? Is it simply an interpolation between two extreme values of one bond distance? If so, were the other structural parameters kept fixed or reoptimized at each point or did the authors compute a minimum energy path?

6. Line 107: Is the crystal structure of an entire enzyme an appropriate starting point for a structure estimate? I would expect that there would be structural differences by bonding to the ligand.

7. Line 114: "Our recent restricted active-space second order perturbation theory (RASPT2) study[33] testified for minor Cu-O2 distance change (~0.03 Å) and revealed sensitive influences on its Cu L-edge spectra." This sentence is unclear. Perhaps the authors could consider rewording it. Were the changes from experiment or theory?

8. Line 117-121: "However, extending across a broader configuration space for comprehensive characterization of the binding dynamics faces
more technical challenges, which require including more relevant active orbitals to achieve smooth PESs." This sentence is not clear and precise. The authors should clearly state the technical challenges.

9. Line 123: A comparison of the spectra with and without ligand for at least one structure would be interesting to justify the choice for removing the ligand.

10. Line 142: ΔETS = ET−ES instead of ΔEST ≡ET−ES

11. Line 196: "We assume the same shift for all CuO2+ structures." Is this approach valid for multireference methods? Is the same active space used for all calculations? The authors should clarify this point for the benefit of new readers.

12. Line 217: The definition of a state vs a molecular orbital is unclear. What do states 72, 144, or 151 refer to?

13. Line 222: It is not necessarily the spectral signatures that simplify but rather the contributions from different MOs? Perhaps the author could clarify this.

14. Figure 3: The values on the left in panels a and b refer to the stretching pathway. Units should be added to the Y-axis. The authors should also discuss why the higher-energy features are missing in the RAS calculations. This may be due to computational cost, but clarifying this point would be helpful for new readers.

15. Line 266: The justification for the core-hole calculations seems incomplete. The authors state "Both methods show a switch in the energy order of the two states (Supplementary Note 2, Fig. S2), with TDDFT/TDA predicting a larger splitting than RASPT2 at 2a. For peak B (O1s → σ ∗), RASPT2 reveals clearer (i.e., without the entanglement of numerous weak transitions)". However, the core-hole calculations are only performed at the DFT level of theory. It looks like the authors want to examine how the orbitals stabilize for the O 1s and Cu 2p core holes. The discussion should be clarified to make this point clear.

16. Line 315: As mentioned earlier, the authors do not present any ultrafast results, and there is no explicit time dependence in the calculations. This is misleading and should be clarified.

I encourage the authors to address the above points to strengthen the manuscript.

Reviewer #2

(Remarks to the Author)
The manuscript presents a DFT and RASPT2 computational study of electron flow and oxidation-state transitions in the CuO2+ system, using a dual-edge (O K-edge and Cu L2,3-edge) X-ray absorption spectroscopy (XAS) framework. The synergistic integration of ligand-centered (O K-edge) and metal-centered (Cu L2,3-edge) spectral simulations may help address the challenges in interpreting complex metal complex dynamics, leveraging the interpretability of O K-edge data to decode Cu L-edge features. Overall, this is an interesting topic, and the presented simulation is well done. There are some concerns about the system and the lack of dynamic simulations.
1. I am not entirely convinced that gas-phase CuO2+ is relevant to catalysis or biology. I would expect this molecule to always bind to a larger framework, which can have a very different structure (and dynamics) from its gas-phase geometry.
2. While the authors claimed that their method "established a dual-edge computational spectroscopy framework that directly tracks coupled electron-nuclear dynamics", there are no dynamics simulations in the manuscript. Considering the small size of the molecule, it should not be difficult to have a real-time dynamics motion to support this claim.
3. As the authors use different basis sets for DFT optimizations (6-31G*, LanL2DZ) and RASPT2/TDDFT spectral simulations (ANORCC-VDZP, def2 series), it is helpful to discuss the influence of basis sets on the results.
4. In Method, the manuscript mentions a "modified LanL2DZ" basis set for Cu but does not specify the nature of the modification or cite the source of the modified basis set.
5. In Figures 2a and 2c, why is there a difference between the last peaks (956.0 and 945.1 eV)?
6. In page 3, "The 0.2 eV relative shift between 2a and 2b matches the 0.3 eV difference between CuO and Cu2O references, further validating our OS assignments", the literature is not cited correctly.

Reviewer #3

(Remarks to the Author)
The authors investigate the electronic structure of Cu-O_2 complexes as a function of the Cu-O bond length. Despite stable molecular orbital occupations along the reaction coordinate, the bonding and antibonding orbital character evolves. As the complex approaches the gas-phase structure with a longer Cu-O bond, the bonding orbital gains Cu 3d character while losing O character, with opposite changes occurring in the antibonding orbital. Atomic charge analysis indicates a gain of 0.2 electrons for Cu in the gas-phase configuration. The comparison of RASPT2-level simulations of O K-edge and Cu L-edge X-ray absorption spectra against experimental data from materials with well-defined oxidation states confirms the transition from Cu(I):O_2 for the gas-phase structure to Cu(II):O_2^- for the crystal structure. A natural transition orbital analysis further elucidated the electronic structure changes, highlighting varying covalent mixing of Cu-O orbitals as a function of bond length. These changes are reflected in the intensity variations of spectral transitions.

This work is solid and thorough, offering a valuable framework to understand how coupled electronic and nuclear dynamics govern redox processes in transition-metal complexes; however, the article requires some revisions and clarifications before publication.

1.The claim of novelty regarding a dual-edge spectroscopy approach is somewhat overstated. Dual-edge strategies are not entirely new, and the authors might consider moderating this phrasing, for example in the abstract ('we introduce a dual-edge computational spectroscopy approach').

2.Regarding the discussion of snapshot-based approaches, the manuscript currently suggests they have been applied primarily to organic molecules and crystals. It would be helpful if the authors could also acknowledge that similar strategies have been used for transition-metal complexes, where geometries from molecular dynamics, surface hopping, or static potential energy scans are employed to compute transient K- or L-edge X-ray absorption spectra. Including a few references to such studies would provide broader context for readers.

3.I find the use of MLCT/LMCT terminology in this context very confusing. In photochemistry and coordination chemistry, MLCT denotes a vertical electronic excitation from a metal-based orbital to a ligand-based orbital, usually initiated by light. In the present system, such an excitation would correspond to a transition from the bonding to the antibonding orbitals. However, the manuscript uses MLCT/LMCT language to describe ground-state covalency changes that arise from Cu–O bond shortening. This reflects ground-state redistribution of electron density, not a true charge-transfer excitation. I suggest avoiding CT terminology in this article, as it may mislead readers and does not accurately capture the underlying electronic-structure evolution. (Page 3, line 166; Page 5, line 259, etc.)

4.I have a question regarding Figure 2a. The authors state that they apply a global energy shift of –5.9 eV to both the crystal and gas-phase calculations. Is the same shift also applied to the LCuO complex, or is this spectrum independently aligned to match its corresponding experimental data? I ask because LCuO is assigned as a Cu(III) species, yet its peak position appears intermediate between those of the other two structures. Energy positions are often taken as indicative of copper oxidation states, but this comparison suggests that peak energies do not directly correlate with formal oxidation state. It would be helpful if the authors could clarify the alignment procedure and comment on the implications for oxidation-state assignment.

5.Finally, I found the following sentence somewhat confusing:
"As the Cu–O bond shortens from 2.24 to 1.82 Å, the L_3 and L_2 main peaks (i and n) intensify due to enhanced Cu 3d character in the particle SO-NTOs (Fig. 4d). This reflects the transition from a fully filled $3d^{10}$ configuration at 2b to a partially filled $3d^9$ state at 2a, which enables more possible 2p → 3d transitions through MLCT."
Apart from the non-standard use of MLCT terminology pointed out before, the sentence appears to mix two distinct effects that contribute to peak intensity: (1) the increase in Cu 3d character in the orbitals, and (2) the change in orbital occupations. The occupation of the antibonding orbital (the target of the transition) does not actually change, as shown in Figure 1b. Therefore, the claim that the $3d^9$ configuration "enables more 2p → 3d transitions" is not accurate. It would be helpful if the authors could clarify this point and clearly separate the contributions from covalency and orbital occupation to the peak intensity.

Reviewer #4

(Remarks to the Author)
I co-reviewed this manuscript with one of the reviewers who provided the listed reports. This is part of the Communications Chemistry initiative to facilitate training in peer review and to provide appropriate recognition for Early Career Researchers who co-review manuscripts.

Version 1:

Reviewer comments:

Reviewer #1

(Remarks to the Author)
The authors have significantly improved the manuscript by including significant additional simulations and by strengthening their discussion of the results. Below are a few minor comments that the authors are encouraged to address to further improve and strengthen their manuscript.

In this study, the authors investigate the relationship between different structures and spectral changes at two different absorption edges. These changes are referred to as ‘time-resolved signals’ by the authors, especially with the inclusion of the BOMD simulations which directly maps spectral changes on a time axis. However, experimental observation of time-resolved spectral signatures on these ultrafast timescales, is generally only achieved by photoexcitation leading to observation of a system in the excited state. To avoid confusion here, we recommend clearly distinguishing these terms. It would be good to highlight that the catalysis reactions involving CuO2 molecules mainly take place in an electronic ground state as justification to keep this study purely in the ground state.

In this revised version of the manuscript the authors include additional BOMD simulations which give additional insights into the dynamic behavior of the spectral features. While this additional section is highly appreciated, the BOMD simulations seem to favor the end-on alignment of the molecule while the previous sections only discuss the side-on structure. The authors may want to split the simulations into two parts and only discuss the side-on structure in the main text, window 2 and the end-on structure could be moved to the SI. This can be justified by mentioning that in the context of a molecular framework or a protein the side-on structure will be preferred due to steric/environmental effects.

p.2 l.71 the authors mention that a mapping of surface hopping full dynamics is feasible for scattering signals but not for spectroscopy features. A short elaboration as to why, may be useful (I assume the authors are referring the scattering simply requiring the structural information not the electronic structure).

p.2 l.128: The authors mention that the O K-edge spectrum is more interpretable but it is unclear what makes this absorption edge more interpretable than the Cu L-edge. Perhaps the authors mean that it is well known that the metal L-edges have a richer structure (with more spectral features) compared to K-edges and typically require a more accurate electronic structure description. Maybe the authors can reword this sentence for clarity. In the same paragraph, perhaps the authors could also add a sentence along the lines "Importantly, both the Cu L2,3 edge and the O K-edge probe the same manifold of unoccupied electronic states, providing complementary element-specific access to the same underlying electronic structure.

p.11 l.561-563: For clarity, the authors should add that the intermediate (interpolated) structures were not optimized along a minimum energy path.

Fig.6 The spectra plotted in panels c and d are hard to read, maybe the scaling could be adjusted.

Reviewer #2

(Remarks to the Author)
In their revision, the authors mentioned that “the series of intermediate structures connecting the endpoints 2a and 2b were generated using a linear interpolation of the Cartesian coordinates to define a simplified binding coordinate dominated by the Cu–O bond length” and this is “a standard practice in photochemical pathway analysis”. I find it hard to picture a reaction that goes directly from reactants to products in such a linear way, regardless of the shape of the potential energy surface. This choice should be better explained or justified. Is this consistent with the BOMD simulation?

The authors have made efforts to include dynamic results. However, there are some points that need clarification regarding the BO molecular dynamics. Which electronic state is the BO dynamics running? I assume the electronic ground state. But if that’s the case, the electron transfer is an adiabatic process driven by thermal effects. How can such processes be probed by time-resolved spectroscopy, which apparently requires a pump defining time zero? The manuscript also emphasizes coupled electron-nuclear motion, but this concept is valid only for nonadiabatic processes with strong vibronic coupling.

Reviewer #3

(Remarks to the Author)
[Editorial Note: This Reviewer did not provide any further comments for the authors]

Reviewer #4

(Remarks to the Author)
I co-reviewed this manuscript with one of the reviewers who provided the listed reports. This is part of the Communications Chemistry initiative to facilitate training in peer review and to provide appropriate recognition for Early Career Researchers who co-review manuscripts.

Version 2:

Reviewer comments:

Reviewer #1

(Remarks to the Author)
I am satisfied with the extensive revisions that the authors have made to strengthen their manuscript. This is a strong paper and can be accepted.

Reviewer #2

(Remarks to the Author)
The authors have addressed my previous comments and I suggest this manuscript to be published.

Reviewer #4

(Remarks to the Author)
I co-reviewed this manuscript with one of the reviewers who provided the listed reports. This is part of the Communications Chemistry initiative to facilitate training in peer review and to provide appropriate recognition for Early Career Researchers who co-review manuscripts.

Open Access This Peer Review File is licensed under a Creative Commons Attribution 4.0 International License, which permits use, sharing, adaptation, distribution and reproduction in any medium or format, as long as you give appropriate credit to the original author(s) and the source, provide a link to the Creative Commons license, and indicate if changes were made.
In cases where reviewers are anonymous, credit should be given to 'Anonymous Referee' and the source.
The images or other third party material in this Peer Review File are included in the article’s Creative Commons license, unless indicated otherwise in a credit line to the material. If material is not included in the article’s Creative Commons license and your intended use is not permitted by statutory regulation or exceeds the permitted use, you will need to obtain permission directly from the copyright holder.
To view a copy of this license, visit https://creativecommons.org/licenses/by/4.0/

Reply to Referee #1:

General Comment:
In this paper, the authors introduce a dual-edge computational X-ray spectroscopy approach to track how electrons move during ultrafast chemical reactions in transition metal complexes. The work focuses on the CuO_2^+ system to understand how changes in geometry drive metal-to-ligand charge transfer. Using computed Cu $L_{2,3}$ and O K-edge X-ray absorption spectra along the Cu-O bond using multiconfigurational RASPT2 and TDDFT methods, the study shows that symmetric Cu-O bond shortening triggers a fast conversion from Cu(I):O_2 to Cu(II):O_2^-. The dual-edge analysis uses the simpler ligand-centered O K-edge to interpret the more complex metal-centered L-edge, providing a clear picture of how electrons flow during bond rearrangement. The authors suggest that this is a general way to interpret ultrafast X-ray data and can be used to follow redox dynamics in catalysts, metalloenzymes, and energy conversion systems to name a few.

The use of dual-edge XAS analysis is not new and has been widely applied in the X-ray literature to understand electronic structure changes driven by bond rearrangements. I encourage the authors to include additional references for the benefit of readers who may be less familiar with this area. However, this paper is fairly well written, and I will support its publication once the authors have addressed the following review comments.

Our General Reply: We sincerely thank you for your constructive and detailed feedback, and for your support of our work. We have carefully addressed each point below, and the corresponding revisions have been incorporated into the manuscript.

On the "dual-edge"
We fully acknowledge that the synergistic use of dual-edge X-ray absorption spectroscopy (XAS) is an established concept. In direct response, we have expanded the Introduction with key references that illustrate its power [Hua et al., *J. Phys. Chem. Lett.* **2019**, *10*, 7172; Lim et al., *J. Am. Chem. Soc.* **2025**, *147*, 7496; Ross et al., *Commun. Phys.* **2024**, *7*, 304; Lätsch et al., *J. Am. Chem. Soc.* **2024**, *146*, 7456] and have clarified our specific advance.

Our central contribution is not simply the use of two edges, but the **strategic use of the more interpretable O K-edge as a spectral key to decode the intricate, dynamically evolving Cu L-edge features** along a reaction coordinate. This approach directly addresses the well-known challenge of interpreting metal L-edge spectra and provides a robust, unambiguous analysis of coupled electronic and geometric changes.

The revised text now reads:

> *The Cu $L_{2,3}$-edge XAS is particularly sensitive for the $3d$ features of copper complexes since the Cu $2p \to 3d$ transitions are electric dipole allowed. Meanwhile, the oxygen K-edge XAS directly probes the O $1s \to 2p$ transitions. The synergistic power of such dual-edge (or multi-edge) X-ray spectroscopy has been well established for elucidating complex electronic and structural dynamics in various systems.[26, 37, 55, 56] Integrating this approach in our study will provide a complementary perspective, helping to characterize the oxidation state of copper in response to the transient electronic and structural dynamics. Building on this framework, the central advance of*

this work is the strategic use of the more interpretable O K-edge spectrum as a key to decode the intricate, dynamically evolving features of the Cu $L_{2,3}$-edge, thereby enabling a robust, real-time analysis of coupled electron transfer and structural dynamics.

1. Overall, the distinction between the binding coordinate and the reaction coordinate is not clear here. Additionally, the work refers to ultrafast processes several times while presenting only spectra calculated for static structures, which is misleading.

Our Reply 1: Thank you for raising these important points about terminology and clarity.

1. Reaction coordinate ξ vs. copper-oxygen distance $r_{\mathrm{Cu-O}}$

To our knowledge, in theoretical chemistry, a reaction coordinate (ξ) is any *collective* variable parameterizing the pathway between reactant and product states. It can be defined in multiple ways, e.g., as an intrinsic reaction coordinate, a specific bond length, or an interpolation in Cartesian or internal coordinates. In this work, two related but distinct variables appear, which we clarify here:

- **Reaction coordinate ξ (binding coordinate)**
  In practice, we define ξ by **linearly interpolating the Cartesian coordinates** between the endpoint structures **2a** and **2b**. This practical approach, which is explicitly described in the Methods, generates a series of intermediate structures along a simplified pathway dominated by the Cu–O bond shortening. Linear interpolation of Cartesian coordinates is a standard and practical approach for generating intermediate structures to explore a reaction pathway [e.g., *ChemPhysChem* 2018, 10.1002/cptc.201800169], particularly when the dominant geometric change is well-defined.

  The procedure has now been explicitly added in the Methods section (end of 1st paragraph):

  > *"Intermediate structures along the binding coordinate were generated by linear interpolation of Cartesian coordinates between the endpoints* **2a** *and* **2b**.*"*

  In this manuscript, we use the terms "binding coordinate" and "binding pathway" interchangeably with "reaction coordinate," where "binding" emphasizes the specific process under study. Other coordinate choices exist, including interpolation via delocalized internal coordinates (DLC), redundant internal coordinates (RIC), natural internal coordinates (NIC), or linear synchronous transit (LST) [*Int. J. Quantum Chem.* 2020, 10.1002/qua.26390]. In our Cartesian interpolation, ξ is effectively dimensionless; for other choices it may carry units, e.g., Å amu$^{1/2}$ in natural internal coordinates [*J. Phys. Chem. A* 2011, 10.1021/jp201327w; *J. Phys. Chem. Lett.* 2019, 10.1021/acs.jpclett.9b02692].

- **copper-oxygen distance $r_{\text{Cu-O}}$**
  For each spectral curve, we label the corresponding geometry by its Cu–O bond length $r_{\mathrm{Cu-O}}$ (in Å). This is **not** the reaction coordinate itself, but a key geometric *descriptor* that varies monotonically along ξ. Spectra are labeled by $r_{\mathrm{Cu-O}}$, providing an intuitive and experimentally relevant measure of the structural evolution.

  We have **refined Figures 3 and 4** for improved clarity, including adjustments to the Y-axis labels and annotations within the figures to better illustrate the relationship between the reaction coordinate ξ and the Cu–O bond distance $r_{\text{Cu–O}}$.

(2). Static vs. ultrafast language

One theme of our group's research focuses on developing computational tools for interpreting ultrafast X-ray spectroscopy of chemical reactions, whether thermal or photochemical. A core methodological theme is the use of **snapshots along potential energy surfaces** to map spectral signatures as a function of a reaction coordinate, $S_{\mathsf{TXAS}}(\omega, \xi)$. (Hua, Mukamel, Luo, *J. Phys. Chem. Lett.* **2019**, *10*, 7172; Hua, *Comput. Phys. Commun.* **2024**, *296*, 109016)

We acknowledge that molecular dynamics (MD) simulations, either fully quantum or mixed quantum-classical, provide a more direct route to modeling explicit time-dependent evolution and can yield $S_{\mathsf{TXAS}}(\omega, \tau)$ directly. However, such approaches are often **computationally prohibitive** for the high-level electronic structure methods required for accurate core-level spectra, especially for transition-metal systems, where multiconfigurational treatments are essential.

The snapshot-based approach offers a **practical, economic** alternative: while it does not propagate explicit time-dependent wavepackets, it efficiently maps spectral evolution along chemically meaningful coordinates, providing **statistically-averaged trends** that can be directly correlated with time-resolved experiments. This methodology has been validated against XFEL data: the simulated dark-state signature in uracil O K-edge spectra (*J. Phys. Chem. Lett.* **2019**, *10*, 7172) matches the experimental thymine transient absorption spectrum (Wolf et al., *Nat. Commun.* **2017**, *8*, 29)(see Figure R1). The method has since been adopted by other groups, for example, in the work of Martínez and co-workers on malonaldehyde (List et al., *Chem. Sci.* **2020**, *11*, 4180).

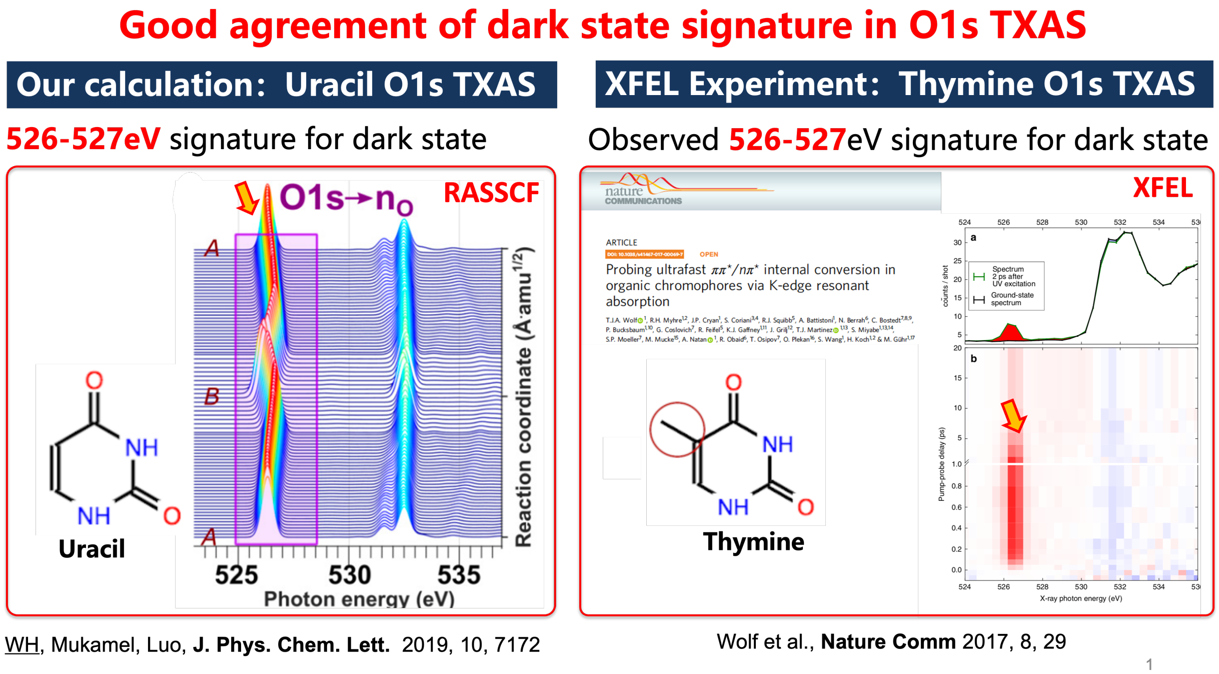

Figure R1: Benchmark of the static snapshot approach against ultrafast experiment. The RASSCF-calculated O K-edge TXAS spectrum of uracil's S_1 dark state matches the experimental thymine TXAS (XFEL), notably capturing the diagnostic feature at 526-527 eV.

The present work studies copper–dioxygen binding, a thermally driven process. The principal challenge here is not the dynamics but the accurate simulation of metal L-edge spectra, which require a multiconfigurational treatment. Our "snapshot" methodology is employed to map the spectral evolu-

tion along the binding coordinate, providing a foundational reference for interpreting future ultrafast measurements. We have carefully revised the text to remove the word "ultrafast" where it could be misinterpreted as describing explicit time-dependent simulations, while retaining it in contexts that refer to the ultimate application of the framework.

Thus, we view static and dynamical approaches as complementary strategies for interpreting ultrafast spectroscopy. In direct response to your suggestion and that of another referee, we have **performed additional Born–Oppenheimer molecular dynamics (BOMD) simulations to investigate the spectroscopic evolution** (with new text added in the last three subsections of the Results and Discussion). The 1.45 ps room-temperature BOMD trajectory samples a broader configurational space, capturing both side-on and end-on geometries, and validates that the electron-transfer process studied in the static approach (covering a much smaller configurational space along a constrained side-on coordinate) indeed occurs on an ultrafast timescale at ambient conditions. Further details are provided in our responses to the specific Comment 3 below.

2. The authors should refine their discussion and specify that they are referring to the field of soft X-ray spectroscopy here (50-1200 eV).

Our Reply 2: Thank you for pointing this out—you are correct. The O K-edge (520–550 eV) and Cu $L_{2,3}$-edge (925–960 eV) spectra simulated in this work both fall within the soft X-ray range. We have added the explicit qualifier "soft" to "X-ray spectroscopy" where appropriate (e.g., in the abstract and introduction).

2'. Line 22: The authors should refer either to transient X-ray spectroscopy, which provides insights into dynamics, or to modern X-ray spectroscopy, which provides information on structural details and oxidation states. Modern spectroscopies are not necessarily time-resolved.

Our Reply 2': We thank you for the scientific rigor. We have explicitly added 'time-resolved'.

3. Line 34-35: The authors should consider rewording their sentence to state that an accurate framework should accurately capture the dynamics as well as predicting the spectral signatures. However, no dynamics were actually simulated in this paper.

Our Reply 3: We sincerely thank the reviewer for raising this important point regarding the interpretation of "accurate framework." In direct response, we have now **performed new Born–Oppenheimer molecular dynamics (BOMD) simulations** and integrated the results throughout the revised manuscript. This includes **three new subsections** in the Results and Discussion:

- Tracking the energy and structural evolution along a BOMD trajectory
- Tracking the spectral evolution along a BOMD trajectory: Cu 2*p* edge
- Tracking the spectral evolution along a BOMD trajectory: O 1*s* edge

accompanied by new **Figures 5-7**.

We fully agree with the reviewer that a comprehensive theoretical framework should, ideally, both **capture the dynamics and predict the spectral signatures**. In this context, we view the static "snapshot" approach and the explicit BOMD approach as **complementary strategies**, each with distinct advantages for connecting structure to spectroscopy:

- The **static approach** (snapshots along potential energy curves) provides an **efficient, statistically well-defined mapping** of spectral evolution against a reaction coordinate, incorporating

thermal averaging at a practical computational cost.

- The **BOMD approach** provides the **explicit time dependence** needed to model specific stochastic events and nonequilibrium pathways, though achieving full statistical convergence for spectra requires extensive (and costly) trajectory sampling.

In this work, the single BOMD trajectory we performed serves to **probe specific dynamical events** (e.g., the side-on to end-on transition and bond oscillations) and to **validate that the key spectral signatures identified statically remain diagnostically meaningful under real-time fluctuations**. Even with this limited sampling, new insights emerged, such as distinct spectral markers for the side-on and end-on configurations. We have **refined the Introduction** to more clearly frame the complementary roles of these two computational strategies.

We believe the revised manuscript now offers a more complete and nuanced theoretical framework that addresses the reviewer's insightful comment.

4. Line 80: It seems that the Cu L-edge would be more sensitive to the oxidation state than the O K-edge. What additional insight is gained from analyzing the O edge? Perhaps this could be connected to potential ligand interactions. It is also understandable that the O K-edge is computationally simpler to treat. The discussion could be refined further to clarify these points for a broader audience.

Our Reply 4: We thank you for raising this important point. While the Cu L-edge, capturing the $3d$ electronic structure via $2p \to 3d$ transitions, is indeed more directly diagnostic of the copper oxidation state, the O K-edge provides complementary insight into the ligand (dioxygen) redox changes. By correlating the two edges, we can track the concurrent oxidation of copper and reduction of O_2, offering a complete picture of the electron-transfer event.

Experimental XAS measurements require theoretical simulations to help with interpretations. Technically, Cu L-edge spectra are more challenging to interpret than O K-edge spectra due to their complex multiplet structure, requiring detailed multiconfigurational methods (e.g., RASPT2) for reliable simulation. In contrast, O K-edge spectra are not only simpler in shape but also generally more accurate and computationally accessible–even with DFT/TDDFT one can often obtain reliable results. As demonstrated in this work, the interpretable and computationally tractable O K-edge can therefore serve as a "decoder" for the more complex Cu L-edge. We have expanded the discussion to clarify these points for a broader audience.

Furthermore, the newly added BOMD simulation shows that both edges provide sensitive spectral signatures for distinguishing side-on and end-on configurations, with the O K-edge proving to be particularly responsive to Cu–O distance fluctuations.

5. Line 86: It is clear that the authors are not modeling ultrafast dynamics but are instead interpolating between a few static structures. As mentioned earlier, this is misleading and should be clarified. The authors should also explain how the intermediate structures were generated. How is the reaction pathway defined? Is it simply an interpolation between two extreme values of one bond distance? If so, were the other structural parameters kept fixed or reoptimized at each point or did the authors compute a minimum energy path?

Our Reply 5: We thank you for these comments. As noted in our Reply 1, we employ a steady-state snapshot-based approach to map spectral evolution along a binding coordinate. This strategy avoids the

need for explicit ensemble averaging over molecular dynamics trajectories–a challenge that is particularly acute for transient X-ray spectra–and provides an economical, validated route to interpreting ultrafast experiments, as demonstrated in earlier work (e.g., Hua et al., *J. Phys. Chem. Lett.* **2019**, *10*, 7172) and adopted by others (e.g., Martínez and co-workers, *Chem. Sci.* **2020**, *11*, 4180).

Intermediate structures were generated by linear interpolation of Cartesian coordinates between the endpoints **2a** and **2b**, a standard practice in photochemical pathway analysis [De Vetta et al.,*ChemPhotoChem* 2018, 10.1002/cptc.201800169]. The procedure is detailed in the Methods:

> *"The series of intermediate structures connecting the endpoints* **2a** *and* **2b** *were generated using a linear interpolation of the Cartesian coordinates to define a simplified binding coordinate dominated by the Cu–O bond length."*

No further geometry re-optimizations were performed at intermediate points, consistent with similar studies that map spectral evolution along an interpolated coordinate [see, e.g., Nachtigallová et al., J. Phys. Chem. A 2011, 10.1021/jp201327w; De Vetta et al., *ChemPhotoChem* 2018, 10.1002/cptc.201800169]. The chosen pathway (gas-phase **2b** → crystal-phase **2a**) spans a chemically meaningful range of Cu–O distances and focuses on the intrinsic response of the Cu–O_2 unit. A full minimum-energy path calculation, while feasible for two gas-phase structures, was not required for our goal of establishing a spectral map along a physically relevant structural coordinate.

The principal computational demand lies in ensuring *smooth* potential-energy curves and spectral profiles across the coordinate. This requires a consistent active space that remains appropriate for all geometries–a stricter condition than single-point calculations at isolated structures. Our approach meets this requirement and yields smooth spectral evolution, which is the central focus of the study.

Additionally, the newly added BOMD simulation provides explicit time resolution of the ultrafast dynamics (as detailed in our response to Comment 4), offering a complementary dynamical perspective to the static-path approach.

6. Line 107: Is the crystal structure of an entire enzyme an appropriate starting point for a structure estimate? I would expect that there would be structural differences by bonding to the ligand.

Our Reply 6: Thanks for your insightful comment. The crystal structure provides an experimentally characterized endpoint for the CuO_2^+ core (**2a**), which is then relaxed to its gas-phase minimum (**2b**). Structures **2b**→**2a** span a chemically relevant range of Cu–O distances (1.82–2.24 Å) that (1) captures substantial structural changes associated with metal–dioxygen binding, while (2) permitting a consistent active space across all geometries–a requirement for obtaining smooth potential energy surfaces and spectral evolution.

Our focus on the bare CuO_2^+ core serves two purposes: it isolates the intrinsic copper–dioxygen interaction from ligand perturbations, and it keeps the active space computationally tractable. We emphasize that CuO_2^+ and $LCuO_2$ represent two distinct electronic systems. The bare core is not intended to approximate $LCuO_2$; rather, it serves as our target system to systematically investigate copper–dioxygen binding.

7.Line 114: "Our recent restricted active-space second order perturbation theory (RASPT2) study[33] testified for minor Cu-O_2 distance change (∼0.03 Å) and revealed sensitive influences on

its Cu L-edge spectra." This sentence is unclear. Perhaps the authors could consider rewording it. Were the changes from experiment or theory?

Our Reply 7: Thank you for highlighting this unclear phrasing. In our previous RASPT2 study [*Inorg. Chem.* **2023**, *62*, 17115], we compared two ligand-bound $LCuO_2$ structures whose Cu–O_2 distances differed by only ∼0.03 Å, and we found that their *simulated* Cu L-edge spectra differed noticeably. The "changes" referred to in the sentence are therefore differences between two theoretical spectra obtained with consistent computational settings (RASPT2, consistent active space and initial guess).

To avoid confusion, we have revised the manuscript to read: "Our recent restricted active-space second-order perturbation theory (RASPT2) study[33] showed that even a minor Cu–O_2 distance change (∼0.03 Å) can sensitively influence the *simulated* Cu L-edge spectra."

We also note that in the earlier study, the presence of the ligand constrained us to a very narrow configurational range to maintain smooth spectral changes. In the present work, removing the ligand allows us to sample a much broader structural range while still preserving the smoothness required for a continuous spectral map.

8.Line 117-121: "However, extending across a broader configuration space for comprehensive characterization of the binding dynamics faces more technical challenges, which require including more relevant active orbitals to achieve smooth PESs." This sentence is not clear and precise. The authors should clearly state the technical challenges.

Our Reply 8: Thank you for requesting clarification. The technical challenges are twofold: (1) maintaining a consistent active space that remains balanced and appropriate across a broad range of geometries, and (2) ensuring smooth potential-energy surfaces (PESs) and spectral evolution. As the sampled configuration space expands, the active space must be enlarged to accommodate orbitals relevant to all structures, significantly increasing computational cost.

The central issue is obtaining *smooth* energy and spectral profiles–discontinuous or erratic changes would render the results unphysical. In our earlier ligand-bound study, smoothness constraints limited us to a narrow geometric window. By removing the ligand in the present work, we can sample a much wider structural range (e.g., Cu–O distances from 1.82 to 2.24 Å) while still preserving the smoothness required for a continuous spectral map.

The sentence has been refined as:

> *"However, extending across a broader configuration space for comprehensive characterization of the binding dynamics faces more technical challenges, principally the requirement to maintain a consistent and sufficiently large active space across all geometries in order to achieve smooth PESs and spectral evolution."*

9. Line 123: A comparison of the spectra with and without ligand for at least one structure would be interesting to justify the choice for removing the ligand.

Our Reply 9: Thank you for this suggestion. A direct comparison of the Cu L-edge spectra with and without ligand is provided in Figure 2a, where complex **1** ($LCuO_2$) contains the ligand and complexes **2a**/**2b** (CuO_2^+) represent the bare core.

We emphasize that these are two distinct systems. **1** was the subject of our previous study [*Inorg. Chem.* **2023**, *62*, 17115], where we performed the first peak-by-peak assignment of its Cu L-edge

spectrum, but could only examine a very narrow configurational range (due to the ligand's electronic complexity).

2 is the focus of the present work, where we intentionally remove the ligand to isolate the intrinsic Cu–O_2 interaction and sample a much broader structural coordinate (1.82–2.24Å). This shift in focus allows us to map smooth spectral evolution across an extended binding pathway—a task that is computationally prohibitive for the ligand-bound system.

10. Line 142: $\Delta E_{\mathrm{TS}} \equiv E_{\mathrm{T}} - E_{\mathrm{S}}$ instead of $\Delta E_{\mathrm{ST}} \equiv E_{\mathrm{T}} - E_{\mathrm{S}}$.

Our Reply 10: We thank you for catching this typo. Since the triplet is the ground state (see Tables S1–S2), the energy difference should be defined as $\Delta E_{\mathrm{ST}} = E_{\mathrm{S}} - E_{\mathrm{T}}$ to yield a positive value. The equation and associated numerical signs have been corrected accordingly.

11. Line 196: "We assume the same shift for all CuO_2^+ structures." Is this approach valid for multireference methods? Is the same active space used for all calculations? The authors should clarify this point for the benefit of new readers.

Our Reply 11: Yes, the same active space is used for all CuO_2^+ structures along the binding coordinate, which makes a uniform energy shift appropriate. In multiconfigurational methods, systematic errors in absolute core-excitation energies (due to, e.g., the frozen-core approximation and basis-set limitations) are largely state-independent and consistent across structures when the active space is kept fixed. Applying a single, global shift obtained by calibrating one reference spectrum (here, **2b** to the experimental Cu(I) energy) therefore preserves the physically meaningful *relative* energy changes across the series.

This calibration protocol has been validated in previous work: our O 1s TXAS calculations on uracil identified a fingerprint at 526–527 eV for its dark S_1 state [Hua *et al.*, *J. Phys. Chem. Lett.* **2019**], and an XFEL experiment on thymine (differing only by a –CH_3 group) observed a nearly identical feature [Wolf *et al.*, *Nat. Commun.* **2017**], validating our approach (see Figure R1).

12. Line 217: The definition of a state vs a molecular orbital is unclear. What do states 72, 144, or 151 refer to?

Our Reply 12: We thank you for this inquiry. The indices (e.g., 72, 144, 151) refer to **spin–orbit coupled eigenstates** in a multi-electron description, not to single-electron molecular orbitals. Each index uniquely labels a final excited state reached by a transition from the ground state (State 1). For instance, "state 72 at 936.6 eV" denotes the transition to the 72nd spin–orbit eigenstate.

For clarity, we have added a brief explanatory note in the text and provide an illustrative excerpt from the OpenMolcas output below, which shows how states are enumerated and how transition strengths are assigned:

```
*********************************************************************
* Spin-orbit section *
*********************************************************************
Total energies including SO-coupling:
::  SO-RASSI State 1 Total energy:  -1803.40308265
...
::  SO-RASSI State 72 Total energy:  -1768.76765341

++ Dipole transition strengths (SO states):
---------------------------------------
for osc.  strength at least 1.00000000E-05
```

```
From To Osc.  strength ...
--------------------------------------
1 21 1.05543333E-02 ...
1 22 4.96715091E-03 ...
1 25 6.26492651E-03 ...
1 26 5.44819532E-04 ...
...
```

13. Line 222: It is not necessarily the spectral signatures that simplify but rather the contributions from different MOs? Perhaps the author could clarify this.

Our Reply 13: We thank you for this inquiry. In this work, our spectral analysis for all Cu L-edge spectra is performed rigorously via spin–orbit natural transition orbitals (SO-NTOs) [Feng et al, J. Chem. Theory Comput. 2021, 10.1021/acs.jctc.1c00776], providing a single-electron picture that dissects each multi-electron transition peak-by-peak. The insets of Figure 2e and 2f list all orbital types responsible for the labeled stick transitions, covering the essential contributors.

It turned out there are some four types at **2a** and two types at **2b**. From the spectral shapes (and underlying numerical components), it is easy to recognize that the two types at **2b** are a subset of the four at **2a**. Strictly speaking, there are indeed different MOs because at the two structures the geometry differs, but they can be considered of the same type, speaking of the linear combination of atomic orbitals.

14. Figure 3: The values on the left in panels a and b refer to the stretching pathway. Units should be added to the Y-axis. The authors should also discuss why the higher-energy features are missing in the RAS calculations. This may be due to computational cost, but clarifying this point would be helpful for new readers.

Our Reply 14:

- **Figure update:** Following your suggestion, we have added the unit "Å" to the Cu–O distance labels near the Y-axis of Figure 3. Y-axis refers to the intensity with arbitrary units, which has now been added explicitly.
- **Missing high-energy features:** You are correct that computational cost is one factor. More importantly, because we employ state-averaged RASSCF (with equal weights for all states by default), including a very large number of high-energy states would reduce the variational accuracy for the lower-energy region that is our primary focus. Our current active space and state selection, therefore, represent a balance between spectral coverage and accuracy for the chemically relevant low-energy excitations. While techniques exist to prioritize low-energy states while still sampling higher-energy ones (e.g., weighted state averaging; see Hua et al., *Struct. Dyn.* **2016**, *3*, 023601), such tuning is beyond the scope of this work. We have added an explanatory sentence in Section 3.2:

> *For peak B (O*$1s \to \sigma^*$*), RASPT2 reveals clearer (i.e., without the entanglement of numerous weak transitions) evolution but lacks high-energy states due to limited state coverage;* **including more states in the RASPT2 calculation would require careful balancing of state weights.***[Struct. Dyn.* **2016***, 3, 023601)]* $\cdots$.

15. Line 266: The justification for the core-hole calculations seems incomplete. The authors state "Both methods show a switch in the energy order of the two states (Supplementary Note 2, Fig. S2), with TDDFT/TDA predicting a larger splitting than RASPT2 at **2a**. For peak B (O1$s \rightarrow \sigma^*$), RASPT2 reveals clearer (i.e., without the entanglement of numerous weak transitions)". However, the core-hole calculations are only performed at the DFT level of theory. It looks like the authors want to examine how the orbitals stabilize for the O 1s and Cu 2p core holes. The discussion should be clarified to make this point clear.

Our Reply 15: Thank you for your careful reading. Figure 3 presents O 1s spectra along the binding coordinate, comparing RASPT2 and TDDFT/TDA–the "both methods" mentioned in the text refer to these two approaches. You are correct that we sought to understand the physical origin of the state-energy reversal relative to the ground state, which is a core-hole effect. For clarity in that analysis, we performed a separate DFT-based core-hole calculation, detailed in Supplementary Note 2A (Fig. S2).

We acknowledge that simply referencing "(Supplementary Note 2, Fig. S2)" was too compact. The expanded text now reads:

> *Both methods exhibit a reversal in the energy ordering of the two states that constitute peak A. The physical origin of this reversal is core-hole relaxation, which we examine via DFT-based core-hole analysis (Supplementary Note 2A, Fig. S2). TDDFT/TDA yields a larger splitting than RASPT2 at* ***2a****. For peak B....*

16. Line 315: As mentioned earlier, the authors do not present any ultrafast results, and there is no explicit time dependence in the calculations. This is misleading and should be clarified.

Our Reply 16: Thank you for reiterating this point. As addressed in our response to Comment 1, our "snapshot" methodology maps spectral evolution along a reaction coordinate, $S(\omega, \xi)$, to provide a static reference for interpreting ultrafast experiments. It does not include explicit time dependence. We have revised the manuscript to avoid any wording that could imply real-time simulations, reserving "ultrafast" for contexts describing the framework's application to time-resolved data.

Furthermore, in direct response to the suggestions in Comments 1 and 3, we have performed BOMD simulations to provide explicit time-dependent insight. The 1.45 ps room-temperature trajectory validates that the electron-transfer process studied statically indeed occurs on an ultrafast timescale under ambient conditions.

Thank you again for your careful review. We believe these revisions and clarifications have significantly strengthened the manuscript, and we hope it now meets the high standards of *Communications Chemistry*.

Reply to Referee #2:

General Comments:
The manuscript presents a DFT and RASPT2 computational study of electron flow and oxidation-state transitions in the CuO_2^+ system, using a dual-edge (O K-edge and Cu $L_{2,3}$-edge) X-ray absorption spectroscopy (XAS) framework. The synergistic integration of ligand-centered (O K-edge) and metal-centered (Cu $L_{2,3}$-edge) spectral simulations may help address the challenges in interpreting complex metal complex dynamics, leveraging the interpretability of O K-edge data to decode Cu L-edge features. Overall, this is an interesting topic, and the presented simulation is well done. There are some concerns about the system and the lack of dynamic simulations.

Our General Reply: We sincerely thank you for your positive assessment of our topic and for finding our simulations well executed. Your constructive feedback has been invaluable in improving the clarity and rigor of our work. We have performed **additional Born-Oppenheimer molecular dynamics (BOMD) calculations** and simulated spectra along the trajectory. Below we address each of your comments.

1. I am not entirely convinced that gas-phase CuO_2^+ is relevant to catalysis or biology. I would expect this molecule to always bind to a larger framework, which can have a very different structure (and dynamics) from its gas-phase geometry.

Our Reply 1: We thank you for raising this important point regarding model system relevance. The gas-phase CuO_2^+ cation is indeed a minimal system, chosen specifically to isolate the **intrinsic** electronic and structural properties of the fundamental Cu–O_2 core common metalloenzymes (e.g., PHM, DβM) [37,38] and to many copper-oxygen catalysts[39-41] Our aim is to elucidate the intrinsic spectroscopic signatures of the core's geometric evolution without the modulation imposed by larger protein or ligand scaffolds, thereby providing a clean reference for interpreting more complex systems.

We acknowledge that realistic catalytic or biological environments involve additional coordinating ligands and solvation. However, capturing such complexity would introduce **additional degrees of freedom for the active space and dramatically increase the computational cost**—particularly for the demanding *multiple* multiconfigurational RASPT2 calculations required for Cu L-edge spectra over a configuration space.

The transferable insight from this study is the computational protocol itself: a dual-edge XAS approach that correlates ligand- and metal-centered spectra to track electron flow. This strategy can be systematically extended to larger, explicitly ligated systems in future work using QM/MM or larger active spaces, once the intrinsic behavior of the core is clearly established.

2. While the authors claimed that their method "established a dual-edge computational spectroscopy framework that directly tracks coupled electron-nuclear dynamics", there are no dynamics simulations in the manuscript. Considering the small size of the molecule, it should not be difficult to have a real-time dynamics motion to support this claim.

Our Reply 2: We thank you for this critical comment, which rightly points out the importance of explicitly including nuclear dynamics to fully support our methodological claim. In direct response to this and similar feedback, we have **significantly expanded the manuscript with new Born–Oppenheimer molecular dynamics (BOMD) simulations**.

We have added **three new subsections** to the Results and Discussion, presenting:

- Tracking the energy and structural evolution along a BOMD trajectory (new **Fig. 5**).
- Tracking the spectral evolution along a BOMD trajectory: Cu $2p$ edge (new **Fig. 6**).
- Tracking the spectral evolution along a BOMD trajectory: O $1s$ edge (new **Fig. 7**).

This new analysis directly addresses your point. While the static "snapshot" analysis along the reaction coordinate efficiently maps the statistically averaged spectral evolution, the BOMD simulations provide the **explicit, real-time nuclear dynamics** that validate and extend our framework. They demonstrate:

- The **direct observation** of structural transitions (e.g., side-on to end-on) and bond oscillations on the femtosecond timescale.
- That the **distinct spectral signatures** identified from static calculations remain robust and diagnostically meaningful under real-time geometric fluctuations.
- The **complementary power** of the dual-edge approach in a dynamic setting, where the O K-edge serves as a sensitive real-time probe for Cu–O bonding changes.

Thus, the revised manuscript now integrates both a static mapping *and* explicit real-time dynamics, which together provide a more complete and robust "computational spectroscopy framework that directly tracks coupled electron-nuclear dynamics," as initially claimed. We have also refined the **Introduction and Conclusions** to more precisely frame the complementary roles of these two computational strategies.

3. As the authors use different basis sets for DFT optimizations (6-31G*, LanL2DZ) and RASPT2/TDDFT spectral simulations (ANO-RCC-VDZP, def2 series), it is helpful to discuss the influence of basis sets on the results.

Our Reply 3: Thank you for this important comment on the basis set choices. We agree that assessing the influence of the basis set is a crucial part of any computational protocol. The core challenge of this work was the accurate simulation of the demanding Cu L-edge XAS, which requires a multiconfigurational (RASPT2) treatment. Therefore, our systematic basis set validation focused specifically on this task, as detailed in **Supplementary Note 3B and Figure S7**.

The selected combinations for other computational stages were chosen based on **established best practices for each task and software environment**, balancing accuracy and efficiency.

Specifically:

- **Geometry Optimizations** (GAUSSIAN 09)**: 6-31G* and modified LanL2DZ**

  We used 6-31G* for light atoms and modified LanL2DZ (with LanL2DZ pseudo potential) for copper. This hybrid combination is a well-established and efficient standard in transition-metal computational chemistry, providing reliable equilibrium geometries at a tractable cost.

- **RASPT2 Spectrum Simulations** (OPENMOLCAS)**: ANO-RCC-VDZP**

  For the high-accuracy calculation of core-level spectra, we employed the ANO-RCC-VDZP basis set. This set is specifically designed within the ANO framework for multiconfigurational methods and includes scalar relativistic corrections. Our validation (Fig. S7) confirmed its suitability for the Cu L-edge, making it the optimal choice for correlated spectroscopy of our system within the OPENMOLCAS platform.

- **TDDFT Spectrum Simulations** (Q-Chem)**: def2-TZVP and def2-QZVP**

  For comparative TDDFT calculations, we used the def2-TZVP and def2-QZVP basis sets. The def2 series is a general-purpose, highly optimized standard for molecular DFT and TDDFT, offering a systematic path to convergence and excellent performance within modern quantum chemistry packages like Q-Chem.

In summary, the basis sets were deliberately selected to match the requirements of each methodological step. Our primary validation effort was logically directed at the most sensitive part of the calculation–the Cu L-edge RASPT2 spectra. The final validation of our protocol is the agreement between the simulated spectra (by RASPT2 and TDDFT) and experiments, as discussed in the manuscript.

We have **added a Supplementary Note 4** ("Note on Basis Set Selection") in the Supporting Information to justify these choices. In the main text, this is briefly referenced in the **Methods section**:

> *Note that different basis sets were chosen only to meet the specific requirements of each methodological stage, and these choices do not compromise the accuracy or conclusions of our work (Supplementary Note 4).*

4. In Method, the manuscript mentions a "modified LanL2DZ" basis set for Cu but does not specify the nature of the modification or cite the source of the modified basis set.

Our Reply 4: We apologize for the omission. We have added the references and more detailed descriptions to the text (in the Methods section):

> *"the modified LanL2DZ basis set with optimized 4p functions [Ref1] and the LanL2DZ pseudo potential for Cu.[Ref2]"*
>
> *[Ref1] Couty, M.; Hall, M. B., J. Comput. Chem. 1996, 17, 1359-1370*
>
> *[Ref2] Hay, P. J.; Wadt, W. R., J. Chem. Phys. 1985, 82, 299-310*

5. In Figures 2a and 2c, why is there a difference between the last peaks (956.0 and 945.1 eV)?

Our Reply 5: Thank you for this question. The value 945.1 eV appears to be a typo; it should be 954.1 eV, corresponding to the last peak of the bottom (red) curve in Figure 2a (Cu(I) reference). To clarify:

- 956.0 eV: final peak in the experimental spectrum of Cu(I) plastocyanin.
- 954.1 eV: final peak in our RASPT2 spectrum of CuO_2^+ structure **2b**.

These values differ by 1.9 eV. This difference arises from several factors:

- **Different complexes, different ligand environments.**
  Although XAS probes the local copper electronic structure, the surrounding ligands affect peak positions. For example:
  - *Comparison 1 (L_3 edge):* Experimental Cu_2O shows peaks at 931.6 and 934.2 eV, while Cu(I) plastocyanin shows 931.4 and 935.4 eV. The first peaks differ by 0.2 eV; the second differ by 1.2 eV.
  - *Comparison 2 (L_2 edge):* Cu(I) plastocyanin peaks are at 951.2 and 956.0 eV; our **2b** peaks are at 951.2 and 954.1 eV. The first agree exactly (0.0 eV difference), the second differ by 1.9 eV.

The 1.2 eV discrepancy in the L_3 second peak is comparable to the 1.9 eV discrepancy here, indicating that ligand-specific effects are the primary cause. Notably, the **first peak in each edge is more consistent across complexes**, making it a more reliable oxidation-state descriptor. Our protocol, which uses the lowest L_3 peak for oxidation-state assignment, remains robust.

- **Inherent deviations in simulation and experiment.**
  Both experiment and theory involve calibration uncertainties. The exact agreement (0.0 eV) for the first L_2 peak validates the accuracy of our method for that feature.
- **Computational challenges of metal L-edge simulations.**
  Metal L-edge calculations are inherently more demanding than K-edge simulations. In our previous study [Wang et al., *Inorg. Chem.* 2023, 10.1021/acs.inorgchem.3c01896], experimental L_2 peaks for $LCuO_2$ were 952.6 and 958.8 eV, while RASPT2 gave 951.9 and 957.1 eV–differences of 0.7 and 1.7 eV, respectively. A 1–2 eV deviation is thus within the expected accuracy of RASPT2 for such spectra.

6. In page 3, "The 0.2 eV relative shift between 2a and 2b matches the 0.3 eV difference between CuO and Cu_2O references, further validating our OS assignments", the literature is not cited correctly.

Our Reply 6: We have added the appropriate citation [Sakata et al., *J. Phys. Chem. B* **2022**, *126*, 2164].

Thank you again for your careful review. We believe these revisions and clarifications have significantly strengthened the manuscript, and we hope it now meets the high standards of *Communications Chemistry*.

Reply to Referee #3:

General Comments:
The authors investigate the electronic structure of Cu-O_2 complexes as a function of the Cu-O bond length. Despite stable molecular orbital occupations along the reaction coordinate, the bonding and antibonding orbital character evolves. As the complex approaches the gas-phase structure with a longer Cu-O bond, the bonding orbital gains Cu 3d character while losing O character, with opposite changes occurring in the antibonding orbital. Atomic charge analysis indicates a gain of 0.2 electrons for Cu in the gas-phase configuration. The comparison of RASPT2-level simulations of O K-edge and Cu L-edge X-ray absorption spectra against experimental data from materials with well-defined oxidation states confirms the transition from Cu(I):O_2 for the gas-phase structure to Cu(II):O_2^- for the crystal structure. A natural transition orbital analysis further elucidated the electronic structure changes, highlighting varying covalent mixing of Cu-O orbitals as a function of bond length. These changes are reflected in the intensity variations of spectral transitions.

This work is solid and thorough, offering a valuable framework to understand how coupled electronic and nuclear dynamics govern redox processes in transition-metal complexes; however, the article requires some revisions and clarifications before publication.

Our General Reply: We sincerely thank you for your positive assessment of our work and your constructive suggestions to strengthen the manuscript. We have carefully considered all points and have revised the text accordingly. Below, we address your specific comments.

1. The claim of novelty regarding a dual-edge spectroscopy approach is somewhat overstated. Dual-edge strategies are not entirely new, and the authors might consider moderating this phrasing, for example in the abstract ('we introduce a dual-edge computational spectroscopy approach').

Our Reply 1: Thank you for your thoughtful comment regarding the novelty of the dual-edge approach. We fully acknowledge that the synergistic use of dual-edge X-ray absorption spectroscopy (XAS) is an established concept. In direct response, we have expanded the Introduction with key references that illustrate its power [Hua et al., *J. Phys. Chem. Lett.* **2019**, *10*, 7172; Lim et al., *J. Am. Chem. Soc.* **2025**, *147*, 7496; Ross et al., *Commun. Phys.* **2024**, *7*, 304; Lätsch et al., *J. Am. Chem. Soc.* **2024**, *146*, 7456] and have clarified our specific advance.

Our central contribution is not simply the use of two edges, but the **strategic use of the more interpretable O K-edge as a spectral key to decode the intricate, dynamically evolving Cu L-edge features** along a reaction coordinate. This approach directly addresses the well-known challenge of interpreting metal L-edge spectra and provides a robust, unambiguous analysis of coupled electronic and geometric changes.

The revised text now reads:

> *The Cu $L_{2,3}$-edge XAS is particularly sensitive for the $3d$ features of copper complexes since the Cu $2p \to 3d$ transitions are electric dipole allowed. Meanwhile, the oxygen K-edge XAS directly probes the O $1s \to 2p$ transitions. The synergistic power of such dual-edge (or multi-edge) X-ray spectroscopy has been well established for elucidating complex electronic and structural dynamics in various systems.[26, 37, 55, 56] Integrating this approach in our study will provide a complementary perspective, helping to characterize the oxidation state of copper in response to the transient electronic and structural dynamics. Building on this framework, the central advance of this work is the strategic use of the more interpretable O K-edge spectrum as a key to decode the*

> *intricate, dynamically evolving features of the Cu $L_{2,3}$-edge, thereby enabling a robust, real-time analysis of coupled electron transfer and structural dynamics.*

2. Regarding the discussion of snapshot-based approaches, the manuscript currently suggests they have been applied primarily to organic molecules and crystals. It would be helpful if the authors could also acknowledge that similar strategies have been used for transition-metal complexes, where geometries from molecular dynamics, surface hopping, or static potential energy scans are employed to compute transient K- or L-edge X-ray absorption spectra. Including a few references to such studies would provide broader context for readers.

Our Reply 2: Thank you for your thoughtful comment. We agree that providing a more balanced discussion regarding snapshot-based approaches for transition-metal complexes would enhance the Introduction. We have revised the relevant part of the text to explicitly acknowledge the application of such methods to metal K- and L-edge X-ray absorption spectra, and we have included illustrative references to support this point.

At the same time, we wish to offer a clarifying note: to the best of our knowledge, while methods employing geometries from molecular dynamics or potential energy surface scans are indeed applied to K-edge spectra, their use for L-edge spectra remains more limited, reflecting the significant computational challenges associated with L-edge simulations, as outlined in our introduction. Furthermore, it is important to note that even for K-edge studies, many applications continue to rely on calculations at one or a few representative geometries, though advances such as MCTDH coupled with vibronic Hamiltonians have also been applied in select cases.

The revised section now reads:

> *"For transition metal L-edges, $\cdots$ The complexity stems not only from performing molecular dynamics simulations at the required electronic structure level but also from simulating the corresponding high-fidelity X-ray spectra, constituting a dual computational challenge. This intrinsic trade-off between general spectroscopic simulation accuracy and dynamical feasibility in transition metal complexes has been highlighted in a recent methodological review.[34]*
>
> *While full-dimensional, non-adiabatic dynamics simulations (such as trajectory surface hopping) have been successfully coupled with X-ray scattering signals to elucidate ultrafast spin-crossover mechanisms in Fe(II) complexes,[35] the analogous task of simulating continuous, peak-resolved L-edge X-ray spectra along dynamical trajectories remains an open challenge. For this reason, most studies of time-resolved metal L-edge X-ray spectra have focused on only one or a few discrete, representative geometries.[23, 36] This approach of analyzing discrete geometries has also been the predominant strategy for interpreting time-resolved metal K-edge spectra.[37] Nevertheless, we note that for the metal K-edge spectra, the interpretation of transient data has been advanced by methods that compute spectra for multiple static geometries displaced along predefined normal modes,[38] as well as by methods like the multi-configurational time-dependent Hartree (MCTDH) approach[39] coupled with vibronic Hamiltonians (and with either finite difference[40] or TDDFT[41] for spectral simulations). However, to the best of our knowledge, a continuous, peak-resolved analysis that tracks the evolution of electronic transitions and their underlying orbital character across a sequence of geometric snapshots remains unavailable, even for metal K-edge spectra."*

3. I find the use of MLCT/LMCT terminology in this context very confusing. In photochemistry and coordination chemistry, MLCT denotes a vertical electronic excitation from a metal-based

orbital to a ligand-based orbital, usually initiated by light. In the present system, such an excitation would correspond to a transition from the bonding to the antibonding orbitals. However, the manuscript uses MLCT/LMCT language to describe ground-state covalency changes that arise from Cu-O bond shortening. This reflects ground-state redistribution of electron density, not a true charge-transfer excitation. I suggest avoiding CT terminology in this article, as it may mislead readers and does not accurately capture the underlying electronic-structure evolution. (Page 3, line 166; Page 5, line 259, etc.)

Our Reply 3: We thank you for this important clarification regarding terminology. You are absolutely correct: in photochemistry and coordination chemistry, **Metal-to-ligand charge transfer (MLCT)** denotes a vertical **electronic excitation** from a metal-based orbital to a ligand-based orbital, whereas **Metal-to-ligand electron transfer (MLET)** describes the **ground-state** redistribution of electron density that results from covalent orbital mixing. Our original use of "MLCT/LMCT" to describe ground-state covalency changes was therefore inaccurate and potentially misleading.

Following your suggestion, we have updated our title to be
"Ultrafast metal-to-ligand **electron** transfer driven by bond shortening revealed with dual-edge computational X-ray spectroscopy",
and **removed all "MLCT/LMCT" terminology** from the manuscript where it referred to ground-state electronic evolution. In its place, we now use precise, literature-consistent terms **MLET/LMET**. For example:

> *"This orbital evolution reveals competing charge transfer processes: (1) Metal-to-ligand ET (MLET) through* $d_{xz} + \pi^*_{ip}$ *stabilization; (2) Ligand-to-metal ET (LMET) through* $d_{xz} - \pi^*_{ip}$ *destabilization. " (end of subsection "Ground-state chemical and electronic structures")*

To frame this distinction clearly for the reader, we have also revised the Introduction to explicitly separate the concepts. The revised text now reads:

> *"In transition metal complexes, charge transfer phenomena—encompassing both ground-state electron redistribution and the corresponding optical excitations (e.g., metal-to-ligand and ligand-to-metal charge transfer, MLCT/LMCT)—are highly pronounced due to accessible redox-active orbitals, making these systems ideal for probing such fundamental dynamics. " (1st paragraph, Introduction)*

This introductory phrasing establishes that our work focuses on the ground-state redistribution component, which we subsequently quantify as MLET/LMET in the Results and Discussion section.

Our usage of MLET aligns directly with prior literature on ground-state redox processes, for example:

- "**Metal-to-Ligand Electron Transfer** in Diiminopyridine Complexes of Mn-Zn. A Theoretical Study", Budzelaar et al., *Inorg. Chem.* **2001**, *40*, 4649
- "Steric control in the **metal–ligand electron transfer** of iminopyridine–ytterbocene complexes", Trifonov et al., *Dalton Trans.* **2018**, *47*, 1566).

We appreciate your guidance in improving the precision and clarity of our presentation.

4. I have a question regarding Figure 2a. The authors state that they apply a global energy shift of -5.9 eV to both the crystal and gas-phase calculations. Is the same shift also applied to the LCuO complex, or is this spectrum independently aligned to match its corresponding experimental data?

I ask because LCuO is assigned as a Cu(III) species, yet its peak position appears intermediate between those of the other two structures. Energy positions are often taken as indicative of copper oxidation states, but this comparison suggests that peak energies do not directly correlate with formal oxidation state. It would be helpful if the authors could clarify the alignment procedure and comment on the implications for oxidation-state assignment.

Our Reply 4: Thank you for this insightful question regarding energy calibration. You correctly note that absolute core-excitation energies from multiconfigurational methods like RASPT2 require a system- and edge-specific global shift, δ, to align with experiment. This shift accounts for limitations such as the frozen-core approximation and basis-set incompleteness. Following established protocol, we determine δ by calibrating one reference spectrum per complex to its experimental counterpart, then apply it uniformly to all calculated structures of that complex.

Specifically:

- For the **CuO_2^+** series (Fig. 2, structures **2a–2b** and intermediates), $\delta = -5.9$ eV was obtained by aligning the calculated Cu(I)-like spectrum (**2b**) to the experimental Cu(I) reference energy (931.5 eV), averaged from values of Cu(I) plastocyanin in *J. Am. Chem. Soc.* **1993**, *115*, 2968–2969 (931.4 eV) and value of Cu_2O *J. Phys. Chem. Lett.* **2022**, *13*, 9573–9580 (931.6 eV).
- For the **$LCuO_2$** complex (**1**), $\delta = -3.9$ eV was determined from alignment to the experimental spectrum reported in *J. Am. Chem. Soc.* **2006**, *128*, 8286–8296, consistent with our previous study (Wang et al., *Inorg. Chem.* **2023**, *62*, 17115), confirming a Cu(III) oxidation state.

The 2.0 eV difference between the two shifts reflects the different electronic screening introduced by the ligand in $LCuO_2$.

This calibration approach has been validated in previous transient XAS studies [e.g., Hua et al., *J. Phys. Chem. Lett.* **2019**, *10*, 7172] and ensures that relative energy changes within each series are physically meaningful (predicted dark state feature of 526-527 eV in this way for uracil matched well with thymine XFEL experiment, see Figure R2).

The intermediate peak position of $LCuO_2$, despite its formal Cu(III) assignment, underscores that absolute edge energy is not a standalone metric for oxidation state; it must be interpreted together with spectral shape, intensity, and complementary O K-edge signatures.

5. Finally, I found the following sentence somewhat confusing: "As the Cu-O bond shortens from 2.24 to 1.82 Å, the L_3 and L_2 main peaks (i and n) intensify due to enhanced Cu 3d character in the particle SO-NTOs (Fig. 4d). This reflects the transition from a fully filled $3d^{10}$ configuration at 2b to a partially filled $3d^9$ state at 2a, which enables more possible Cu $2p\rightarrow 3d$ transitions through MLCT." Apart from the non-standard use of MLCT terminology pointed out before, the sentence appears to mix two distinct effects that contribute to peak intensity: (1) the increase in Cu 3d character in the orbitals, and (2) the change in orbital occupations. The occupation of the antibonding orbital (the target of the transition) does not actually change, as shown in Figure 1b. Therefore, the claim that the $3d^9$ configuration "enables more Cu $2p\rightarrow 3d$ transitions" is not accurate. It would be helpful if the authors could clarify this point and clearly separate the contributions from covalency and orbital occupation to the peak intensity.

Our Reply 5: Thank you for this careful reading. We agree that the original sentence was unclear in

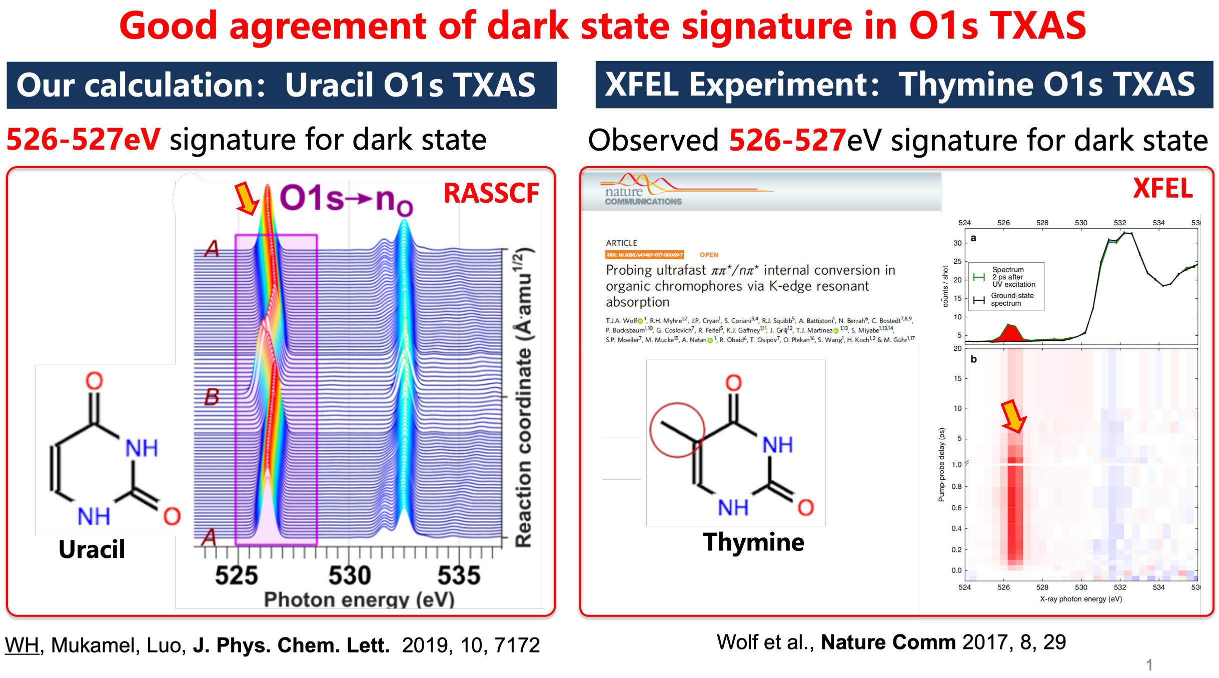

Figure R2: Performance of RASSCF (uracil) validated by XFEL experiment (thymine).

conflating two different descriptions of electronic structure and contained incorrect terminology.

The notation "$3d^{10}$" or "$3d^9$" corresponds to the formal d-electron count in the **atomic orbital picture**, characteristic of Cu(I) and Cu(II) oxidation states, respectively. Meanwhile, Figure 1c shows the **molecular orbital occupations** of the bonding (σ) and antibonding (σ^*) orbitals, which remain stable at 2 and 1 electrons, respectively, throughout the geometric change. These are complementary descriptions: the molecular orbital picture describes the delocalized bonding framework, while the atomic orbital picture tracks the formal oxidation state change through the local electron count.

We have revised the text to clarify this distinction and have removed the misleading "MLCT" reference. The corrected passage now reads:

> *"As the Cu–O bond shortens from 2.24 to 1.82 Å, the L_3 and L_2 main peaks (i and n) intensify. In the molecular orbital picture, this enhancement arises from increased Cu $3d$ character in the particle SO-NTOs (Fig. 4d), which strengthens the $2p \rightarrow 3d$ transition dipoles. This description aligns with the atomic orbital view: the system evolves from a Cu(I) $3d^{10}$ configuration at* ***2b*** *toward a Cu(II) $3d^9$ configuration at* ***2a****. The transition from a fully filled to a partially filled $3d$ shell enhances excitation intensity because the $3d^9$ configuration allows the excited electron to occupy an orbital vacancy, creating a stable $3d^{10}$ final state with favorable electron correlation$\cdots$"*

Thank you again for your careful review. We believe these revisions and clarifications have significantly strengthened the manuscript, and we hope it now meets the high standards of *Communications Chemistry*.

Reply to Referee #4:

Remarks:
I co-reviewed this manuscript with one of the reviewers who provided the listed reports. This is part of the Communications Chemistry initiative to facilitate training in peer review and to provide appropriate recognition for Early Career Researchers who co-review manuscripts.

Our Reply: We sincerely thank you for your time and effort in reviewing our manuscript.

Reply to Referee #1:

General Comment:
The authors have significantly improved the manuscript by including significant additional simulations and by strengthening their discussion of the results. Below are a few minor comments that the authors are encouraged to address to further improve and strengthen their manuscript.

Our General Reply: We thank you for your positive assessment and thoughtful suggestions. Your comments have helped us further improve the manuscript. Below we address each point.

1. In this study, the authors investigate the relationship between different structures and spectral changes at two different absorption edges. These changes are referred to as 'time-resolved signals' by the authors, especially with the inclusion of the BOMD simulations which directly maps spectral changes on a time axis. However, experimental observation of time-resolved spectral signatures on these ultrafast timescales, is generally only achieved by photoexcitation leading to observation of a system in the excited state. To avoid confusion here, we recommend clearly distinguishing these terms. It would be good to highlight that the catalysis reactions involving CuO2 molecules mainly take place in an electronic ground state as justification to keep this study purely in the ground state.

Our Reply 1: We thank you for this important observation. You correctly note that time-resolved signals are often associated with photoexcitation into excited states. We agree that we must be precise in distinguishing our framework from conventional UV-pump experiments. To address this, we have made several substantial revisions throughout the manuscript.

1. Clarifying the pump modality: IR pump vs. UV pump

In pump-probe transient X-ray absorption spectroscopy (TXAS), the choice of pump determines the nature of the dynamics under investigation:

- **Ultraviolet-visible (UV-Vis) pump:** Promotes the system to valence-excited states, initiating photochemical reactions and excited-state dynamics. This is the most common approach in the literature.
- **Infrared (IR) pump:** Resonantly excites vibrational coherences while leaving the system on the **ground electronic state**. This enables the study of thermally driven processes, such as the structurally-driven electron transfer that is the focus of our work.
- **Terahertz (THz) pump:** Similarly accesses ground-state dynamics, typically coupling to low-frequency lattice or collective vibrations (used for solids).

In our study, the electron transfer in CuO_2^+ is a thermally driven process occurring on the ground-state potential energy surface. We therefore propose an **IR pump–X-ray probe** experimental setup, where the IR pulse initializes vibrational coherence and sets time zero, and the subsequent X-ray probe tracks the coupled structural and electronic evolution. This framework is fundamentally distinct from UV-pump experiments that access excited states, and we have taken care to make this distinction explicit.

We have made corresponding changes to the manuscript as follows:

1. **Introduction refined:** We have substantially revised the Introduction to emphasize that our work focuses on ground-state dynamics. On page 2, left column, we now state:

   > *"Given that thermally driven electron transfer in CuO_2^+ occurs on the ground state, we propose an IR pump–X-ray probe setup (Fig. 1f), where an IR pulse initiates vibrational coherences on the PES. While the X-ray probe inherently involves transitions to core-excited manifolds, the observed spectral evolution acts as a sensitive mapping of the underlying ground-state orbital reorganization and covalent mixing (Fig. 1b–e)."*

2. **New figure panel:** We have added **Figure 1f**, which schematically illustrates the proposed IR pump–X-ray probe experiment, making our intent visually clear to the reader.

3. **Vibrational analysis:** To support the feasibility of the proposed IR-pump scheme, we have performed **additional calculations** of the vibrational frequencies for structures **2a** and **2b**. These results are now provided in the newly added **Supplementary Note 2D**, along with **Supplementary Figures S1 and S7**. The vibrational modes and their frequencies are discussed, providing guidance for potential experimental realizations.

   It is important to note that these frequency calculations led to a significant revision of our structural assignment. In the original version, we described **2b** as a minimum-energy structure. However, the vibrational analysis now reveals that **2b** possesses one imaginary frequency, confirming it as a **transition state** on the gas-phase potential energy surface, connecting two equivalent end-on minima. This finding, which we have incorporated throughout the revised manuscript, does not alter our scientific conclusions but rather strengthens them: it reinforces that the symmetric side-on geometry (**2a**) requires environmental constraints (such as those in an enzyme active site) for stabilization, while the isolated cation favors an end-on configuration. The interpolated pathway between **2b** and **2a** therefore represents a meaningful model for the enzyme-constrained binding process, precisely as we intend to study.

2. Clarifying the dual approach to dynamics: static vs. dynamical frameworks

You also raised a valuable point about our use of the term "time-resolved" in the context of both static PES scans and BOMD simulations. We have refined the Introduction to better articulate how these two strategies serve complementary roles in addressing the same dynamics problem.

- The **static approach** (spectral snapshots along a reaction coordinate) is computationally economical and practical. It provides a well-defined mapping of the statistically averaged spectral evolution, offering an overview of how spectra transform as the system moves along a key coordinate.
- The **dynamical approach** (BOMD simulations) is computationally demanding and difficult to converge for statistical averages, but it excels at capturing stochastic events, transient configurations, and the explicit time-dependence of spectral fluctuations. It reveals what the static average obscures.

Both approaches are valid strategies for understanding time-resolved signals, and we have substantially revised the Introduction (pages 2–3) to better explain this complementary philosophy. We hope this clarifies that our use of "time-resolved" encompasses both the statistically averaged view (static snapshots) and the explicit time-domain view (BOMD), each providing essential and distinct insights into the underlying dynamics.

2. In this revised version of the manuscript the authors include additional BOMD simulations which give additional insights into the dynamic behavior of the spectral features. While this additional section is highly appreciated, the BOMD simulations seem to favor the end-on alignment of the molecule while the previous sections only discuss the side-on structure. The authors may want to split the simulations into two parts and only discuss the side-on structure in the main text, window 2 and the end-on structure could be moved to the SI. This can be justified by mentioning that in the context of a molecular framework or a protein the side-on structure will be preferred due to steric/environmental effects.

Our Reply 2: We thank you for this thoughtful suggestion and for your appreciation of the additional BOMD simulations. You raise a valid point about focus, and we have carefully considered whether to relegate the end-on analysis to the Supporting Information. After thorough deliberation, we believe that including **both the side-on and end-on windows in the main text** provides essential scientific value that would be diminished by splitting them. We have, however, strengthened the narrative to better justify this inclusion.

1. Why both windows belong in the main text

The BOMD trajectory reveals a spontaneous transition from a side-on to an end-on configuration, which is itself a key scientific finding. This dynamic behavior demonstrates that:

1. The symmetric side-on geometry (**2a**) is metastable in the gas phase, consistent with our revised assignment of **2b** as a transition state (as discussed in our response to your first comment). The system naturally evolves toward the thermodynamically preferred end-on configuration when not constrained by an environment.
2. The stark contrast between the spectral signatures of the two windows validates the extraordinary sensitivity of both the Cu L-edge and O K-edge to the coordination geometry. The side-on window exhibits a characteristic double-peak structure at the Cu L_3-edge and pronounced oscillations in the O K-edge σ^* feature, while the end-on window shows a simplified spectrum dominated by Cu $2p \rightarrow 4s$ transitions and a stabilized O–O σ^* feature.
3. This comparison directly supports a central conclusion of our work: the O K-edge, particularly the oscillatory behavior of the σ^* feature, serves as a superior real-time probe of Cu–O bond fluctuations. This diagnostic power is most clearly demonstrated by contrasting window 1 with window 2.

2. Strengthening the contextual narrative

We agree with you that the side-on geometry is the biologically relevant motif, stabilized by steric constraints in enzyme active sites such as those found in PHM and DβM. We have therefore strengthened the framing around this point. In the revised manuscript (pages 7-8), we now explicitly state:

> *"A marked decrease in total energy after ∼400 fs correlates with an overall elongation of the Cu–O bonds, signaling a structural transition from a predominantly side-on to the thermodynamically preferred gas-phase end-on configuration... This observed evolution carries an important implication: the symmetric side-on geometry, while central to enzymatic function in constrained environments such as the active sites of copper oxygenases, is metastable for the isolated cation. Its spontaneous interconversion to the end-on configuration yields distinct spectroscopic signatures that we now examine in detail."*

This framing accomplishes two goals: (1) it acknowledges that the end-on form is the gas-phase ground state, and (2) it reinforces why the side-on form—despite being metastable in vacuum—is the relevant species for enzymatic contexts, precisely because environmental constraints would prevent the transition we observe in the unconstrained BOMD.

3. Connection to Reply 1

This narrative also connects directly to our response to your first comment. The fact that **2b** is a transition state connecting two end-on minima, and that our BOMD spontaneously evolves away from the side-on geometry, consistently tells the same story: the symmetric side-on CuO_2^+ motif requires environmental stabilization. By presenting both windows, we provide the complete picture—the spectroscopically rich but metastable side-on form and the simpler but thermodynamically preferred end-on form—allowing readers to appreciate why enzymatic environments are necessary to harness the reactive potential of the side-on configuration.

4. Improving figure clarity

Finally, as noted in our response to Comment 6, we have consolidated and enlarged the BOMD spectral figures (now Figure 6) to improve readability. Especially, the 1D cuts of two windows are now in one panel. We believe the enhanced presentation makes the comparison between the two windows clearer and more accessible to the reader.

We hope this explanation convinces you that retaining both windows in the main text strengthens the manuscript's scientific message. We are, of course, open to further suggestions if you remain concerned about focus.

3. p.2 l.71 the authors mention that a mapping of surface hopping full dynamics is feasible for scattering signals but not for spectroscopy features. A short elaboration as to why, may be useful (I assume the authors are referring the scattering simply requiring the structural information not the electronic structure).

Our Reply 3: We thank you for this observation. You are right that in the context of trajectory-based non-adiabatic dynamics (e.g., surface hopping), simulating X-ray scattering signals is computationally straightforward because the signal is primarily determined by the nuclear pair distribution functions (i.e., geometric coordinates).

In contrast, simulating metal L-edge X-ray absorption spectroscopy demands a high-level description of the local electronic structure for every snapshot. Accurate L-edge simulations require multiconfigurational methods to properly treat static and dynamic correlation, multiplet effects, and spin-orbit coupling (SOC). Performing such expensive electronic structure calculations for the thousands of geometries generated in surface hopping trajectories remains a massive computational bottleneck—far exceeding the cost of extracting structural factors from coordinates for scattering simulations.

Following your suggestion, we have added a brief elaboration in the **Introduction (top of Page 2, left column)** to explicitly state the reason for this challenge:

> *Indeed, while non-adiabatic dynamics (e.g., surface hopping) have been coupled with X-ray scattering to elucidate ultrafast spin-crossover,[cite] the analogous task for L-edge spectra remains an open challenge. Accurately simulating continuous, peak-resolved L-edge spectra along trajectories requires treating SO coupling and multiplet effects across a vast ensemble of electronic states–a*

computational cost far exceeding that for scattering signals, which are primarily determined by nuclear pair distribution functions.

4. p.2 l.128: The authors mention that the O K-edge spectrum is more interpretable but it is unclear what makes this absorption edge more interpretable than the Cu L-edge. Perhaps the authors mean that it is well known that the metal L-edges have a richer structure (with more spectral features) compared to K-edges and typically require a more accurate electronic structure description. Maybe the authors can reword this sentence for clarity. In the same paragraph, perhaps the authors could also add a sentence along the lines "Importantly, both the Cu $L_{2,3}$ edge and the O K-edge probe the same manifold of unoccupied electronic states, providing complementary element-specific access to the same underlying electronic structure.

Our Reply 4: We thank you for this insightful comment regarding the interpretability of different X-ray edges.

You are correct that spectral interpretability is closely tied to the quality and complexity of the required theoretical simulations. As noted in the Introduction (paragraph 4), Cu L-edge calculations are substantially more demanding than O K-edge calculations due to spin–orbit coupling, multiplet effects, and relativistic contributions. The relevant text reads:

> *However, extending this rigor to transition metal L-edges is substantially more demanding, as accurate treatment requires accounting for spin-orbit (SO) coupling, multiplet effects, and dynamic correlation.[citations]···*

These effects necessitate multiconfigurational methods such as RASPT2 for the Cu L-edge. In contrast, for the O K-edge, the simpler and computationally more efficient TDDFT method can usually provide accurate spectra (Figure 3a-b). This difference in theoretical complexity is what we mean by "more interpretable."

Following your suggestion, we have checked that the complementary roles of both edges are already stated in the same paragraph, and are now refined as

> *This dual-edge framework leverages the metal L-edge's sensitivity to $3d$ features and the ligand K-edge's direct probe of ligand environments. The synergistic power of such dual-edge (or multi-edge) X-ray spectroscopy has been well established for elucidating complex electronic and structural dynamics.[citations] The central advance of this work is the strategic use of the more interpretable O K-edge as a key to decode the intricate features of the Cu $L_{2,3}$-edge.*

However, we respectfully note that the Cu $L_{2,3}$-edge and O K-edge do not probe the same manifold of unoccupied states, owing to their different selection rules (Cu $2p \to 3d$ vs. O $1s \to 2p$). This is evident from the final-state orbital characters shown in the revised Figure 6c (right) and 6f (right). To improve clarity and compactness, we have now merged the original Figures 6 and 7 into a single **new Figure 6**.

5. p.11 l.561-563: For clarity, the authors should add that the intermediate (interpolated) structures were not optimized along a minimum energy path.

Our Reply 5: We have added explicitly in the Methods. The text now reads

> *Intermediate structures along the binding coordinate were generated by linear interpolation of*

Cartesian coordinates between the endpoints **2a** *and* **2b, without further geometry optimization.**

6. Fig.6 The spectra plotted in panels c and d are hard to read, maybe the scaling could be adjusted.

Our Reply 6: Thank you for this helpful suggestion. To improve readability and compactness, we have merged the original Figures 6 and 7 into a single **new Figure 6**. The original Fig. 6c–d are now combined into a single panel (new Fig. 6c). We adjusted the scaling for better visual clarity, moved text labels outside the plot area to avoid crowding, and removed redundant labels. The revised figure is now significantly clearer and more effectively conveys the spectral evolution at both edges.

Thank you again for your careful review. We believe these revisions and clarifications have significantly strengthened the manuscript, and we hope it now meets the high standards of *Communications Chemistry*.

Reply to Referee #2:

1. In their revision, the authors mentioned that "the series of intermediate structures connecting the endpoints 2a and 2b were generated using a linear interpolation of the Cartesian coordinates to define a simplified binding coordinate dominated by the Cu–O bond length" and this is "a standard practice in photochemical pathway analysis". I find it hard to picture a reaction that goes directly from reactants to products in such a linear way, regardless of the shape of the potential energy surface. This choice should be better explained or justified. Is this consistent with the BOMD simulation?

Our Reply 1: We thank you for your critical comments, which help us clarify the methodological framework of our study. To address them systematically, we structure our response from the general methodological philosophy to the specific computational details.

1. Born–Oppenheimer molecular dynamics (BOMD)

Molecular dynamics simulations offer direct access to ultrafast structural dynamics. Depending on whether thermal or photochemical processes are involved, either BOMD or nonadiabatic molecular dynamics (NAMD) should be employed. Spectra computed along the resulting trajectories can, in principle, yield time-resolved spectroscopic signatures.

However, simulating transient spectroscopy with trajectory-based methods poses a fundamental challenge in **statistical averaging**. Unlike steady-state spectroscopy, transient spectroscopy requires a large ensemble of trajectories to adequately sample the relevant nuclear configurations. For nonadiabatic dynamics, this corresponds to constructing a **compact** representation of the evolving nuclear wavepacket; for BOMD, it reflects the need for **compact** sampling of the classical phase space distribution. Moreover, **longer simulation time leads to greater trajectory divergence, further increasing the number of trajectories required to achieve convergence**.

For these reasons, we do not attempt to compute a fully converged transient spectrum from BOMD in this work. With a single trajectory, we can observe **individual dynamical events** but not statistically converged spectra. In fact, even a single trajectory remains computationally demanding: spectral calculations at a single snapshot require approximately **6 hours** per absorption edge. Other researchers have likewise limited their investigations to **one or a few** trajectories to capture mechanistic events, while addressing statistical sampling through complementary static approaches. For instance, Prof. Todd Martínez at Stanford University and co-workers adopted such a strategy (where the application is photochemistry, but the philosophy is similar):

- N. List et al., T. Martínez, *Chem. Sci.* **11**, 4180 (2020). DOI: 10.1039/d0sc00840k

2. Static approach

Static approaches are designed to overcome the statistical limitations inherent in dynamical methods. The potential energy surfaces (PESs) already encode the relevant statistical information, making this strategy particularly effective when a dominant reaction coordinate can be identified—whether a single structural parameter or a collective variable.

3. Complementary roles of BOMD and static approaches in this work

Following your thoughtful suggestion from the previous revision, our current work integrates both

approaches, offering broader chemical insights. They provide **complementary** perspectives on the problem, as detailed in Table R1. A direct one-to-one comparison is not feasible, as it would require a large ensemble of trajectories spanning the same configurational space covered by the static approach.

Table R1: Complementary roles of static and BOMD approaches in this work

**Approach**	**Purpose**	**Statistical Convergence**	**Configuration space**
Static approach	**Statistical average**: provides the ensemble-averaged spectral trend as a function of the reaction coordinate	Yes (by construction: each point represents a well-defined geometry; the coordinate itself is the averaging parameter)	side-on, symmetric
BOMD	**Event detection**: captures the spectral signatures of specific, rare, or stochastic events (e.g., side-on/end-on transition, asymmetric fluctuations)	No (deliberately single-trajectory; designed to reveal, not to average)	side-on and end-on, asymmetric

Consistency in the overlapping region. In the configuration space where the two approaches overlap—the side-on region—they show excellent agreement. The Cu–O bond lengths in BOMD Window 1 oscillate between approximately 1.8 Å and 2.2 Å(Figure 5c), closely matching the range sampled in our static interpolation (1.82–2.24 Å). This consistency motivated our selection of Window 1 for subsequent spectral analysis.

The spectral assignments are also generally consistent between the two methods:

- **Consistent peak labels** (i, j, k, l, ...) are used for the Cu L-edge in Figure 4a and Figure 6c.
- **Consistent peak labels** (A, B) are used for the O K-edge in Figure 3a-b and Figure 6f.

Divergence beyond the symmetric manifold. The BOMD trajectory extends beyond this overlapping region, spontaneously transitioning to an end-on geometry not represented in the symmetric interpolation. This divergence does not indicate inconsistency; rather, it reveals the system's natural tendency to escape the symmetric manifold in the absence of environmental constraints—a dynamical feature that the static approach, by design, does not capture. The resulting intensity differences between the two methods arise from the BOMD's access to *asymmetrical* structures versus the static interpolation's restriction to *symmetrical* ones. With a sufficiently large ensemble of trajectories, the contributions from the two inequivalent oxygen atoms would likely become more balanced, enabling a more rigorous dynamical comparison. However, such an ensemble calculation remains prohibitively expensive.

4. Revised assignment of 2b as a transition state

Inspired by comments from you and Referee 1, we performed **new vibrational frequency calculations**.

These calculations led to a significant revision of our structural assignment: while we originally described **2b** as a minimum-energy structure, the vibrational analysis now reveals that **2b** possesses one imaginary frequency, confirming it as a **transition state** on the gas-phase potential energy surface, connecting two equivalent end-on minima.

Importantly, this finding does not alter our scientific conclusions but rather strengthens them. It reinforces that the symmetric side-on geometry (**2a**) requires environmental constraints (such as those in an enzyme active site) for stabilization, while the isolated cation favors an end-on configuration. The interpolated pathway between **2b** and **2a** therefore remains a meaningful model for the enzyme-constrained binding process that is the focus of our study.

5. Structural interpolation: justification and validation

We appreciate the opportunity to clarify this point. Our statement that interpolation is "a standard practice in photochemical pathway analysis" refers specifically to connecting stationary points via structural interpolation to generate a pathway for spectral analysis—not to asserting that the actual dynamics follows a linear path.

Methodological context. Structural interpolation is widely used in static studies of chemical reactions to connect special points such as minima, transition states, and conical intersections. The chosen reaction coordinate can be a specific structural parameter or a collective variable. The generated pathways can be constructed using methods such as linear interpolated internal coordinates (LIIC); see, for example:

- D. Nachtigallová et al., H. Lischka, *J. Phys. Chem. A* **115**, 5247 (2011). DOI: 10.1021/jp201327w
- W. Hua, S. Mukamel, Y. Luo, *J. Phys. Chem. Lett.* **10**, 7172 (2019). DOI: 10.1021/acs.jpclett.9b02692

In this work, given the relative simplicity of the molecular structure, we performed interpolation directly in Cartesian coordinates, which yields smooth structural evolution. This approach is also documented in the literature, for example:

- M. De Vetta, L. González, I. Corral, *ChemPhotoChem* **3**, 727 (2019). DOI: 10.1002/cptc.201800169

Validation of the interpolated path. Several lines of evidence support the validity of our interpolated path for spectral analysis:

- The ground-state PEC presented in Figure 4c shows a smooth profile without abrupt changes.
- The O K-edge (Figure 3a-b) and Cu L-edge (Figure 4a) spectra evolve smoothly along the path.
- The natural transition orbitals (NTOs) also evolve smoothly (Figures 3c-d, 4b).

Limitations and alternative approaches. A more sophisticated approach would involve computing the minimum energy path (MEP) by optimizing geometries with frozen coordinates along the interpolation. This would slightly modify the PECs in Figure 4c and the intermediate spectra, but would not alter our conclusions.

We opted not to compute the MEP because of the challenges associated with core-excited states. Obtaining smooth PECs for core-excited states is the most demanding aspect of our static approach, requiring careful grid tuning and iterative adjustments. Directly interpolated structures offer greater computational efficiency for this purpose.

We emphasize that structures **2a** and **2b** represent only a small region of the full configurational space.

Nevertheless, this system serves as a prototype for investigating how oxidation state changes couple with geometric distortions. The simplicity of the ground-state PES provides a clear background against which we can focus on the more challenging aspects of core excitations, particularly the Cu $2p$ edge.

We hope this detailed response addresses your concerns and clarifies the methodological choices and their justification. We are grateful for this insightful feedback, which has helped us strengthen the manuscript.

2.The authors have made efforts to include dynamic results. However, there are some points that need clarification regarding the BO molecular dynamics. Which electronic state is the BO dynamics running? I assume the electronic ground state. But if that's the case, the electron transfer is an adiabatic process driven by thermal effects. How can such processes be probed by time-resolved spectroscopy, which apparently requires a pump defining time zero? The manuscript also emphasizes coupled electron-nuclear motion, but this concept is valid only for nonadiabatic processes with strong vibronic coupling.

Our Reply 2: We thank you for this insightful comment, which touches on several important conceptual distinctions. We address each point systematically below.

1. Ground-state BOMD for thermal reactions versus NAMD for photochemical reactions

You are absolutely correct: the BOMD simulation is performed on the **ground electronic state**. We are modeling a thermal reaction, where the system remains on the ground-state potential energy surface throughout the dynamics.

For photochemical reactions, the simulation would indeed require nonadiabatic molecular dynamics (NAMD) to account for transitions between multiple valence-state PESs. An early implementation applied to ultrafast X-ray spectra simulations, coauthored by one of us (W.H.), can be found in:

- W. Hua *et al.*, S. Mukamel, *Struct. Dyn.* **3**, 023601 (2016). DOI: 10.1063/1.4933007

Both BOMD and NAMD are trajectory-based methods, used for thermal and photochemical reactions, respectively.

2. Establishing time zero: IR pump–X-ray probe

You raise an important question about how time zero can be defined for a thermal reaction. This is achieved through an **infrared (IR) pump–X-ray probe** scheme: a resonant IR pulse initiates vibrational coherences on the ground-state PES, defining time zero, while subsequent X-ray pulses track the ensuing structural and electronic evolution.

This approach is conceptually similar to our recent work on proton transfer in organic crystals, where we proposed an IR/THz pump–X-ray probe scheme to initiate ground-state dynamics:

- G. Ge *et al.*, W. Hua, *J. Phys. Chem. Lett.* **15**, 6051 (2024). DOI: 10.1021/acs.jpclett.4c01133

Discussions toward experimental realization are underway with Prof. K. Ueda at the Shanghai soft X-ray free-electron laser facility (SXFEL).

To directly address your comment, we have made the following revisions to the manuscript:

- **Introduction refined:** We now explicitly distinguish IR-pump (ground-state) from UV-pump (excited-state) experiments:

While ultraviolet-visible pumps initiate dynamics on excited electronic states,[citations] infrared (IR) pumps resonantly excite vibrational coherences on the electronic ground state.[citations] Given that thermally driven ET in CuO_2^+ occurs on the ground state, we propose an IR pump–X-ray probe setup (Fig. 1f), where an IR pulse initiates vibrational coherences on the PES. While the X-ray probe inherently involves transitions to core-excited manifolds, the observed spectral evolution acts as a sensitive mapping of the underlying ground-state orbital reorganization and covalent mixing (Fig. 1b–e).

- **New figure panel:** Added **Figure 1f** with the caption:

  "(f) IR pump–X-ray probe setup proposed to detect the binding: a resonant IR pulse initiates vibrational coherences ($t = 0$) on the ground-state PES, while subsequent X-ray pulses track the transient electronic and structural reorganization."

- **Vibrational analysis:** Performed **new frequency calculations** for **2a** and **2b** to identify suitable IR pump frequencies; results are provided in **Supplementary Note 2D, Figures S1 and S7**.

3. Dynamics versus static approaches: the challenge of statistical convergence

We would like to emphasize that achieving statistical convergence for time-resolved spectroscopy remains a fundamental challenge for any dynamical simulation approach, whether in photochemistry or thermochemistry.

In **photochemical studies** utilizing NAMD, the initial laser-excited wavepacket rapidly disperses as it explores multiple excited-state PESs. Similarly, in **thermal reactions** simulated via BOMD, the system undergoes extensive thermal fluctuations as it moves along the ground-state PES.

In both cases, as the simulation time increases, the evolving system explores an ever-expanding volume of configuration space. This structural spreading leads to an increasingly diffuse distribution of nuclear geometries. To maintain a statistically robust representation of this dispersed ensemble and to accurately resolve the transient spectral features of short-lived species, a proportionally larger number of trajectories is required for averaging. Consequently, the computational cost to reach spectral convergence grows significantly with propagation time, often making dynamical simulations prohibitively expensive compared to snapshot-based sampling methods.

To overcome this difficulty, we have developed a unified **"static-for-dynamics"** framework (the "snapshots along PESs" method) that leverages the statistical information already encoded in the potential energy surfaces. This approach is valid for both thermal and photochemical reactions, as summarized in Table R2.

Table R2: Comparison of dynamical frameworks and experimental contexts for thermal and photochemical reactions.

Reaction Type	Dynamics Basis	PESs Involved	Ultrafast Experiment
Thermal reaction	BOMD	Ground state	IR/THz pump–X-ray probe
Photochemical reaction	NAMD	Multiple valence states	UV pump–X-ray probe

4. "Coupled electron-nuclear motion" versus "nonadiabatic coupling"

We appreciate your rigorous distinction between these terms. You are correct that in the adiabatic framework of BOMD, the system remains on a single potential energy surface, and thus "nonadiabatic

coupling" (involving transitions between multiple electronic states) is not the mechanism at play.

In our study, the focus is on how the oxidation state—an electronic property—changes during structural evolution. The context of "coupled electron-nuclear motion" refers to this scenario: the electron distribution and nuclear geometry are interdependent, evolving together in response to thermal fluctuations. As we state in the Introduction, "structural changes-induced OS changes" and "oxidation-induced structural changes" are two sides of the same coin—they are coupled, even in the absence of nonadiabatic transitions.

From an experimental perspective, X-ray absorption spectroscopy is uniquely sensitive to this coupling. Since core-level energies and transition intensities are functions of both the local geometry and the effective charge (oxidation state), the transient signals directly map the nuclear dynamics onto the electronic energy landscape.

Regarding the X-ray probe itself, we follow the standard "probe-before-destroy" principle common in XFEL and HHG experiments. The core-excitation event captures the instantaneous electronic and structural information of the evolving ground-state ensemble at a specific time delay. As the experiment can be conducted using a gas-phase jet or fresh-sample replenishment, the subsequent destruction or ionization of the specific molecule by the X-ray pulse does not affect the physical picture of the pre-probing thermal dynamics.

We have significantly refined the **Introduction** to explicitly state that our study remains within the adiabatic regime on the **ground electronic state**, and we have removed any language that might confuse this point.

Thank you again for your careful review. We believe these revisions and clarifications have significantly strengthened the manuscript, and we hope it now meets the high standards of *Communications Chemistry*.

Reply to Referee #3:

[Editorial Note: This Reviewer did not provide any further comments for the authors]

Reply to Referee #4:

I co-reviewed this manuscript with one of the reviewers who provided the listed reports. This is part of the Communications Chemistry initiative to facilitate training in peer review and to provide appropriate recognition for Early Career Researchers who co-review manuscripts.

Our Reply: We sincerely thank you for your time and effort in reviewing our manuscript.
